# Flavonoid carbamate hybrids: design, synthesis, and evaluation as multi-target enzyme inhibitors for Alzheimer's disease†

**DOI:** 10.1039/d5ra02267c

**Published:** 2025-05-20

**Authors:** The-Huan Tran, Dai-Nhat-Huy Doan, Thi-Cam-Nhung Cao, Thai-Son Tran, Thanh-Dao Tran

**Affiliations:** a Faculty of Pharmacy, University of Medicine and Pharmacy at Ho Chi Minh City Ho Chi Minh 700000 Vietnam daott@ump.edu.vn; b Faculty of Pharmacy, University of Medicine and Pharmacy, Hue University Hue 530000 Vietnam

## Abstract

Alzheimer's disease is characterized by cholinergic dysfunction and neuroinflammation, with acetylcholinesterase and monoacylglycerol lipase emerging as important therapeutic targets. In this study, a series of novel flavonoid carbamate derivatives were synthesized from chrysin and kaempferol, and their structures were confirmed *via* NMR and HRMS spectroscopy. The inhibitory activities of these compounds were evaluated against acetylcholinesterase and monoacylglycerol lipase using *in vitro* enzymatic assays. Among them, C3 and C5 exhibited significant dual inhibition, with IC_50_ values of 22.86 μM and 46.65 μM for monoacylglycerol lipase, and 61.78 μM and 89.40 μM for acetylcholinesterase, respectively. Molecular docking studies revealed key binding interactions, while molecular dynamics simulations demonstrated their stability within the active sites of target enzymes. These findings highlight C3 and C5 as promising candidates for further investigation in the development of dual acetylcholinesterase/monoacylglycerol lipase inhibitors for Alzheimer's disease treatment.

## Abstract 2

Molecular hybridization of flavonoids with carbamate moieties led to novel compounds with potent AChE and MAGL inhibition, providing a promising multi-target strategy for Alzheimer's disease treatment.

## Graphical abstract

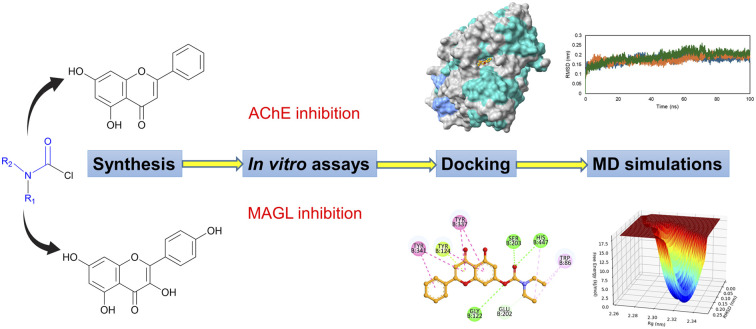

## 1. Introduction

Alzheimer's disease (AD) is a progressive neurodegenerative disorder that severely affects memory and cognition, with an increasing number of cases reported worldwide.^1^ The pathogenesis of AD is highly complex, involving multiple biochemical pathways such as β-amyloid plaque accumulation, mitochondrial dysfunction, neuroinflammation, oxidative stress, Tau hyperphosphorylation, and, notably, the impairment of the cholinergic neurotransmission system.^2–4^ Current treatments include cholinesterase inhibitors (galantamine, donepezil, rivastigmine) and the NMDA receptor antagonist memantine, which primarily focus on symptom management and provide modest improvements in cognition and function.^5,6^ Recently, monoclonal antibodies such as aducanumab and lecanemab have been approved, targeting β-amyloid plaques to slow disease progression, although their efficacy and safety continue to be evaluated.^7^ This highlights the urgent need for novel therapeutic strategies, particularly multi-target approaches, which simultaneously act on multiple pathological mechanisms to achieve greater efficacy.^8–11^

Among the key biological targets of AD, acetylcholinesterase (AChE) and monoacylglycerol lipase (MAGL) have emerged as two enzymes directly associated with disease pathogenesis. AChE is responsible for hydrolyzing acetylcholine, a neurotransmitter crucial for memory and learning.^12^ The overactivity of AChE leads to acetylcholine depletion, contributing to cognitive decline in AD.^13^ Therefore, AChE inhibition is an essential therapeutic strategy to maintain acetylcholine levels in the brain.^14^ Meanwhile, MAGL is the primary enzyme that degrades 2-arachidonoylglycerol (2-AG), an endocannabinoid with neuroprotective and anti-inflammatory effects.^15,16^ Inhibiting MAGL enhances 2-AG levels, reduces neuroinflammation, and mitigates β-amyloid accumulation, a major factor in AD pathogenesis.^17,18^

In enzyme inhibitor design, the carbamate group plays a crucial role by carbamoylating the enzyme's active site, prolonging its inhibitory effect and enhancing therapeutic efficacy.^19^ This has been demonstrated in drugs such as rivastigmine, an AChE inhibitor containing a carbamate moiety, and JZL-184, a MAGL inhibitor with a similar structure.^20,21^ The incorporation of the carbamate group in AChE and MAGL inhibitor design may open new avenues for AD treatment. Additionally, flavonoids, a class of natural compounds found in plants, have been shown to exhibit various beneficial biological activities, including antioxidant, anti-inflammatory, and neuroprotective properties.^22,23^ Chrysin and kaempferol, two representative flavonoids, have demonstrated memory-enhancing effects, AChE inhibition, β-amyloid reduction, and neuronal protection.^24–26^

**Fig. 1:**
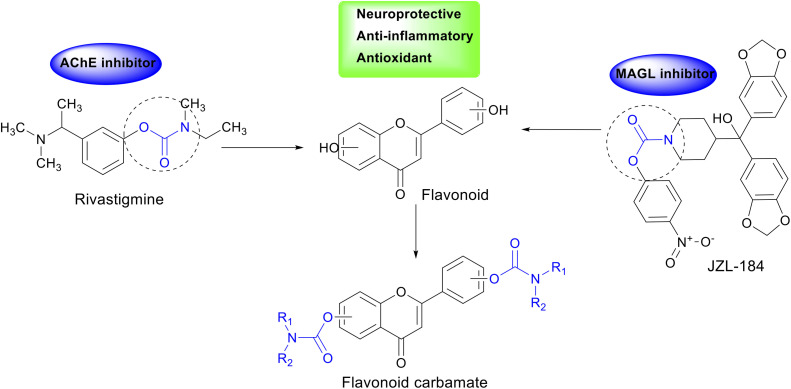
Design strategy for hybrid flavonoid carbamate derivatives.

Molecular hybridization, which combines pharmacophoric features from different chemical scaffolds, has emerged as a powerful tool in drug design and development.^27–29^ In this study, we designed and synthesized carbamate-hybrid derivatives of chrysin and kaempferol to integrate the neuroprotective properties of flavonoids with the potent enzyme inhibition mechanism of the carbamate moiety (Fig. 1). The newly synthesized hybrids will be evaluated for their *in vitro* AChE and MAGL inhibitory activities, alongside *in silico* studies to elucidate their interaction mechanisms with the target enzymes. The findings from this study may contribute to the development of potential multi-target therapeutic agents for AD, aiming for improved efficacy over current treatment options.

## 2. Results and discussion

### 2.1 Synthesis

**Scheme 1:**
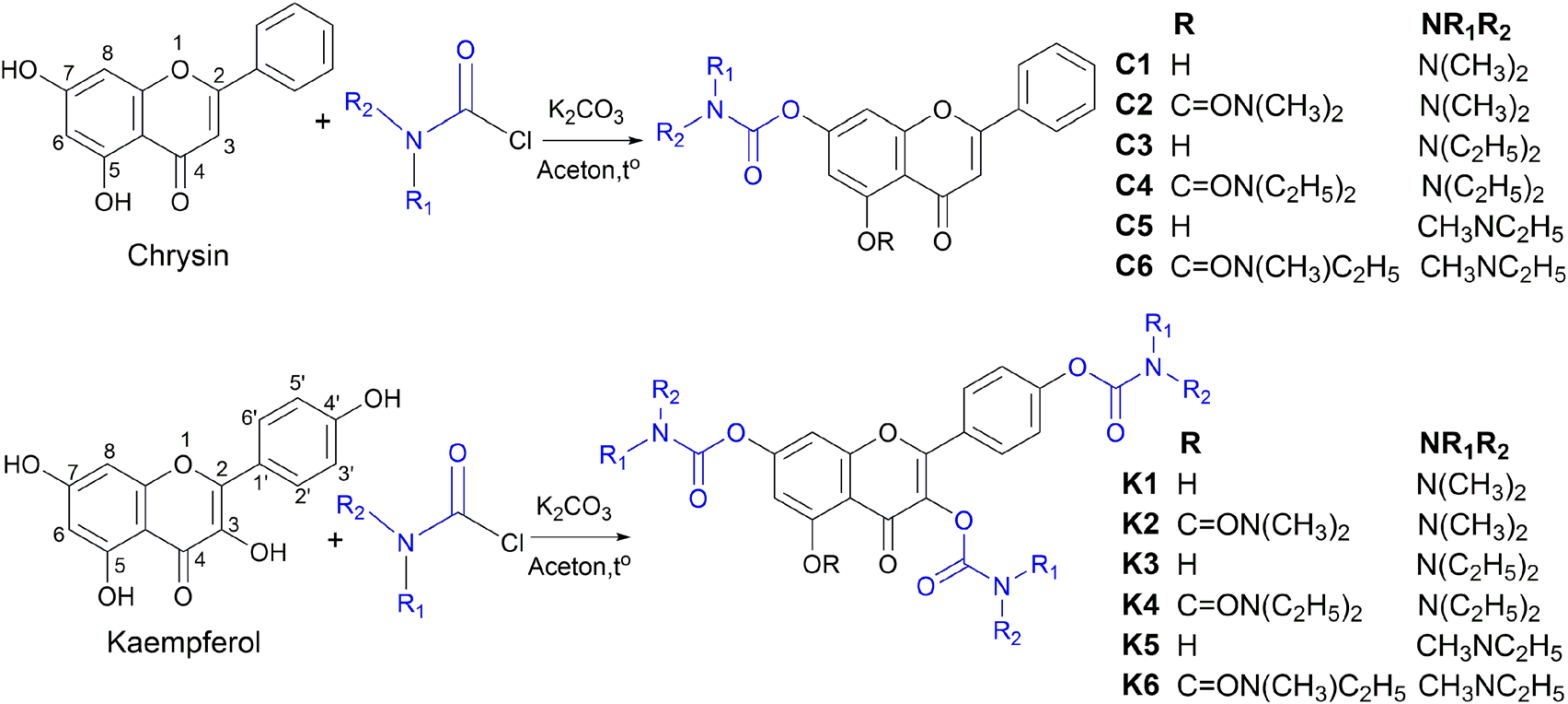
Synthesis of hybrid flavonoid carbamate derivatives from chrysin and kaempferol.

In this study, twelve flavonoid carbamate derivatives were successfully synthesized from two starting materials, chrysin (a flavone) and kaempferol (a flavonol), *via* reactions with various carbamoyl chloride reagents (Scheme 1). The reaction proceeds *via* a nucleophilic substitution mechanism, where the hydroxyl group of chrysin or kaempferol is deprotonated by K_2_CO_3_, forming a more nucleophilic alkoxide intermediate. This intermediate subsequently attacks the electrophilic carbonyl carbon of the carbamoyl chloride, leading to the formation of the desired carbamate derivative with the release of HCl as a byproduct. The synthesis yields of the compounds ranged from 53% to 79%, indicating a highly efficient synthetic approach.

The efficiency of the carbamoylation process in this study was influenced by both electronic and steric factors. The intramolecular hydrogen bond between the hydroxyl group at C_5_ and the carbonyl moiety at C_4_ significantly reduces the nucleophilicity of the hydroxyl group at C_5_, leading to lower reactivity compared to the free hydroxyl groups at other positions. This observation aligns with previously reported trends in flavonoid derivatization, where regioselectivity is dictated by both electronic effects and steric hindrance.^30,31^

The nature of the carbamoyl chloride reagent also played a crucial role in determining the reaction rate. Notably, *N*,*N*-dimethylcarbamoyl chloride exhibited the highest reactivity, leading to shorter reaction times, whereas *N*,*N*-diethylcarbamoyl chloride, with stronger electron-donating alkyl substituents on the nitrogen, exhibited the slowest reaction rate. This trend can be attributed to the electronic effects of the *N*-substituents, which modulate the electrophilicity of the carbonyl carbon, thereby affecting the overall reaction rate.

Ultraviolet (UV) spectral analysis in methanol revealed characteristic absorption peaks in the range of 209–341 nm for the synthesized compounds, reflecting an expansion of the conjugated system upon carbamate substitution. High-resolution mass spectrometry (HRMS) spectra provided definitive evidence of the molecular weights of the compounds, with minimal deviations from the calculated values, demonstrating high accuracy in molecular formula determination.

Nuclear magnetic resonance (NMR) spectral analysis provided clear evidence for the formation of the carbamate-substituted derivatives. In the ^1^H NMR spectra, the characteristic hydroxyl (OH) proton signals of the parent flavonoids at *δ* ∼ 10–12 ppm disappeared, indicating carbamate bond formation. The appearance of new signals at *δ* ∼ 3.0–3.5 ppm (NCH_3_) and *δ* ∼ 3.3–3.5 ppm (NCH_2_) confirmed the attachment of the carbamate substituent. Meanwhile, protons on the benzopyran ring appeared in the *δ* ∼ 6.5–8.1 ppm region as doublet or double-doublet signals, reflecting spin–spin coupling interactions between aromatic protons. In the ^13^C NMR spectra, the presence of the carbonyl (C=O) group was confirmed by characteristic signals at *δ* ∼ 170–182 ppm, while the flavonoid ring system exhibited signals in the *δ* ∼ 100–160 ppm range. The carbamate moiety was further confirmed by signals at *δ* ∼ 35–40 ppm (NCH_3_) and *δ* ∼ 40–45 ppm (NCH_2_). A comparison between the chrysin-derived compounds (C1–C6) and the kaempferol-derived compounds (K1–K6) revealed that the kaempferol derivatives contained additional hydroxyl groups, leading to the presence of more signals in the NMR spectra and influencing the chemical shifts of certain carbon signals in the flavonoid core.

### 2.2 Biological activity

The evaluation of AChE and MAGL inhibitory activity of the twelve flavonoid carbamate derivatives provided crucial insights into the influence of carbamate substitution on enzyme inhibition. Overall, some compounds exhibited notable inhibitory activity, particularly against MAGL, suggesting the potential development of novel inhibitors derived from natural flavonoids (Table 1).

**Table 1:** IC_50_ values of the compounds against AChE and MAGL

Comp.	IC_50_ ± SD (μM)	
	AChE	MAGL
C1	188.14 ± 16.21	72.19 ± 5.34
C2	>200	96.44 ± 1.54
C3	61.78 ± 1.42	22.86 ± 1.30
C4	>200	>200
C5	89.40 ± 5.66	46.65 ± 2.23
C6	>200	>200
K1	100.20 ± 7.85	126.27 ± 8.28
K2	>200	>200
K3	145.85 ± 10.44	61.62 ± 3.01
K4	>200	>200
K5	>200	60.15 ± 4.74
K6	>200	177.90 ± 9.12
Chrysin	156.57 ± 11.74	168.97 ± 11.40
Kaempferol	226.41 ± 17.92	188.97 ± 7.89
Rivastigmine	17.07 ± 0.78	NT^a^
JZL-184	NT^a^	0.057 ± 0.003

a NT: not tested.

Regarding AChE inhibition, although the synthesized derivatives did not exhibit activity as strong as the reference compounds, several compounds still showed moderate inhibition, particularly C3 (IC_50_ = 61.78 ± 1.42 μM), C5 (IC_50_ = 89.40 ± 5.66 μM), and K1 (IC_50_ = 100.20 ± 7.85 μM). This suggests that the introduction of the carbamoyl group into the flavone system may enhance enzyme affinity, highlighting a promising direction for further optimization to improve activity. More notably, some compounds displayed significant MAGL inhibition, with C3 (IC_50_ = 22.86 ± 1.30 μM), C5 (IC_50_ = 46.65 ± 2.23 μM), K5 (IC_50_ = 60.15 ± 4.74 μM), and K3 (IC_50_ = 61.62 ± 3.01 μM) emerging as the most promising candidates.

A clear trend was observed: fully carbamate-substituted derivatives (C2, C4, C6, K2, K4, K6) exhibited little to no activity against both enzymes (IC_50_ > 200 μM). This suggests that excessive substitution may hinder enzyme interactions, possibly due to steric hindrance or the necessity of the OH group for binding at the active site. Conversely, derivatives retaining a free hydroxyl group, such as C3, C5, K1, and K3, demonstrated significant activity, indicating that optimizing the position and type of substitution could substantially enhance enzyme inhibition.

The results indicate that some flavonoid carbamate derivatives exhibit dual inhibitory activity against both AChE and MAGL, particularly C3 and C5, highlighting their potential as dual inhibitors. The discovery of compounds targeting both biological enzymes represents a crucial approach, as it may optimize pharmacological efficacy while minimizing adverse effects compared to single-mechanism drugs.

### 2.3 Molecular docking study

The molecular docking study provided crucial insights into the interaction mechanisms between flavonoid carbamate derivatives and the two target enzymes, AChE and MAGL (Table 2 and S1†). For AChE, the compounds exhibited Δ*G*_B_ values ranging from −6.7 to −10.4 kcal mol^−1^, with most derivatives showing better binding affinity than the reference compound rivastigmine. Key observed interactions included hydrogen bonding with critical amino acids such as Ser203 and His447 in the active site, along with hydrophobic interactions involving Trp86, Tyr337, and Tyr341. These interactions play a vital role in AChE inhibition, enhancing inhibitory activity and stabilizing the inhibitor within the enzyme's active pocket.

**Table 2:** Binding affinities (Δ*G*_B_) of flavonoid carbamate derivatives to AChE and MAGL enzymes

Comp.	Δ*G*_B_ (kcal mol^−1^)		Comp.	Δ*G*_B_ (kcal mol^−1^)	
	AChE	MAGL		AChE	MAGL
C1	−9.2	−10.5	K3	−6.7	−8.3
C2	−10.4	−9.4	K4	−7.9	−7.7
C3	−9.4	−10.3	K5	−8.3	−10.2
C4	−10.4	−9.4	K6	−8	−7.7
C5	−9.4	−10.4	Chrysin	−9.9	−9.8
C6	−10.3	−9.3	Kaempferol	−9.9	−9.8
K1	−9.2	−9.7	Rivastigmine	−7.2	ND^a^
K2	−9	−8.2	JZL-184	ND^a^	−9.8

a ND: not docked.

For MAGL, the Δ*G*_B_ values ranged from −7.7 to −10.5 kcal mol^−1^, with many derivatives exhibiting lower binding energies compared to their parent flavonoids. High-affinity compounds typically formed hydrogen bonds with residues such as Ala51, Met123, and Leu241, as well as hydrophobic interactions with Ile179, Leu184, and Tyr194. Compared to the reference inhibitor JZL-184, some derivatives exhibited comparable or even better Δ*G*_B_ values, indicating promising MAGL inhibition potential.

**Fig. 2:**
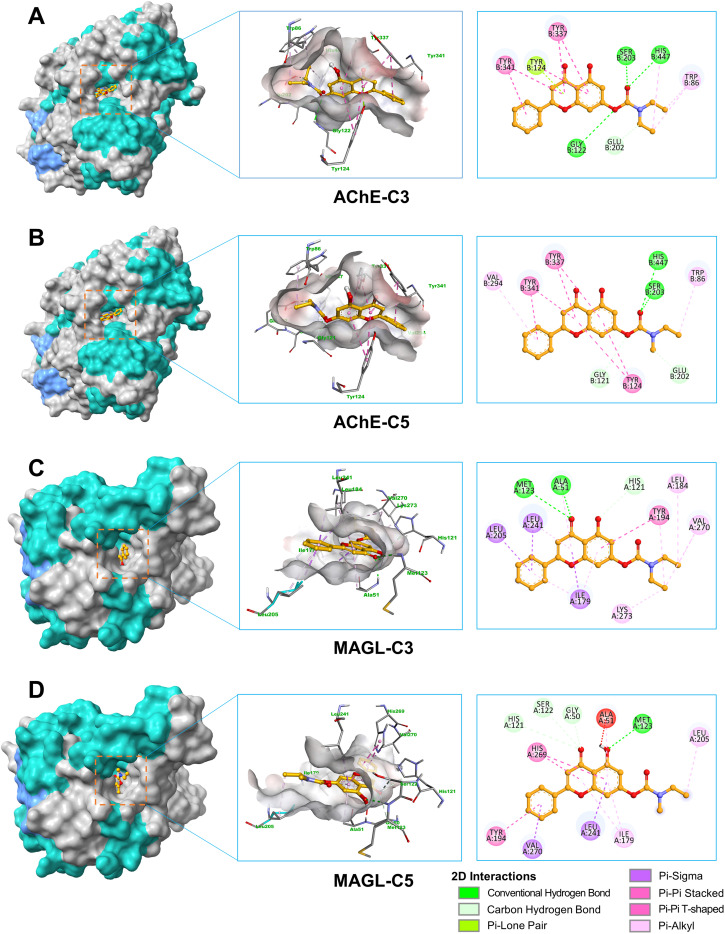
3D and 2D interactions of ligands with AChE and MAGL. (A) Interaction between AChE and C3. (B) Interaction between AChE and C5. (C) Interaction between MAGL and C3. (D) Interaction between MAGL and C5.

Among all tested compounds, C3 and C5 stood out due to their relatively low Δ*G*_B_ values for both enzymes, which correlated well with their strong inhibitory activity in *in vitro* assays, suggesting a strong agreement between docking and biological evaluation results. For AChE, both C3 and C5 exhibited Δ*G*_B_ values of −9.4 kcal mol^−1^, which were lower than that of rivastigmine, indicating high binding affinity. These two compounds formed hydrogen bonds with Ser203 and His447, two key catalytic triad residues essential for AChE function.^32^ Additionally, C3 established an extra hydrogen bond with Gly122. Furthermore, both compounds engaged in multiple hydrophobic interactions with Trp86, Tyr124, Tyr337, and Tyr341, contributing to the stabilization of the ligand-enzyme complex (Fig. 2A and B).

Regarding MAGL, C3 and C5 also demonstrated strong binding affinities, with Δ*G*_B_ values of −10.3 kcal mol^−1^ and −10.4 kcal mol^−1^, respectively, outperforming several other derivatives. Both compounds formed hydrogen bonds with Met123, along with hydrophobic interactions involving Ile179, Leu184, Tyr194, Leu205, Leu241, and Val270. Notably, C5 also exhibited a π–π hydrophobic interaction with His269, a key residue within the enzyme's catalytic triad (Fig. 2C and D).^33^ Overall, the carbamate group significantly contributed to the ligand-enzyme interactions, enhancing the stability of the ligand-enzyme complex through hydrogen bonds and hydrophobic interactions.

Some other derivatives, such as C4 and C6, showed low Δ*G*_B_ values but weak *in vitro* activity, indicating that strong binding affinity does not always translate to effective inhibition. This discrepancy may stem from factors like solubility, chemical stability, and ligand flexibility in solution. Docking provides static binding predictions, but real interactions are dynamic. Ligands must interact optimally with key catalytic residues, and unfavorable steric or electrostatic effects could weaken binding. Additionally, solvent exposure and induced fit effects may alter stability.

The observed correlation between docking studies and *in vitro* biological assays suggests that C3 and C5 have the potential to inhibit both AChE and MAGL simultaneously, aligning with the multi-target paradigm for AD treatment. The structures of these two compounds could be further optimized to improve selectivity and pharmacological efficacy. Based on these findings, C3 and C5 emerge as promising candidates for further investigation using molecular dynamics (MD) simulations, which would allow for the evaluation of ligand-enzyme complex stability under physiological conditions.

### 2.4 Molecular dynamics simulation study

MD simulations were conducted for both apoprotein systems (AChE and MAGL) and protein–ligand complexes (C3 and C5 with the enzymes) to evaluate the stability of the enzymes and the persistence of ligand binding within the active site. Key parameters, including root-mean-square deviation (RMSD), root-mean-square fluctuation (RMSF), radius of gyration (*R*_g_), solvent-accessible surface area (SASA), and molecular mechanics/generalized Born surface area (MM/GBSA) binding free energy, were analyzed to gain insights into protein flexibility and the molecular interaction mechanisms between the ligands and target enzymes.

#### 2.4.1 Protein stability

**Fig. 3:**
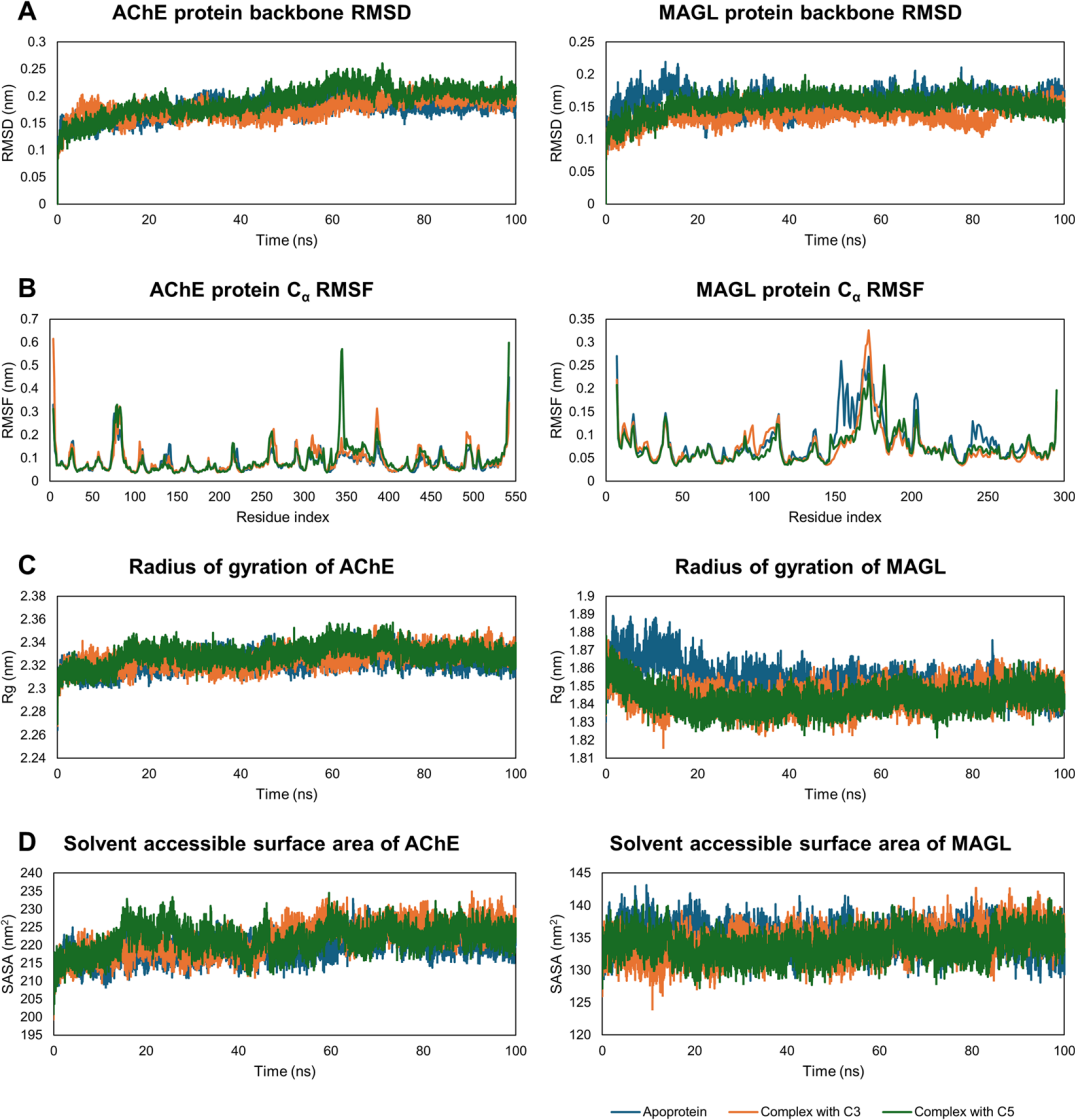
The structural stability of AChE and MAGL in both apoprotein and ligand-bound forms was analyzed over a 100 ns MD simulation. Backbone RMSD plots (A) and residue-specific *C*_α_ RMSF calculations (B) were used to evaluate structural deviations and flexibility. The radius of gyration (C) provided insights into structural compactness, while SASA measurements (D) assessed solvent exposure throughout the simulation.

The 100 ns MD simulation provided valuable insights into the structural dynamics of AChE and MAGL in their free states and in complexes with ligands C3 and C5. RMSD analysis indicated that the proteins reached equilibrium after approximately 10–20 ns and remained stable throughout the rest of the simulation (Fig. 3A). The average RMSD values of the apoproteins (0.175 ± 0.018 nm for AChE and 0.159 ± 0.015 nm for MAGL) demonstrated that both enzymes maintained high structural stability during the simulation. Upon binding to C3 and C5, the RMSD of AChE and MAGL did not change significantly, fluctuating within 0.137–0.191 nm, suggesting that the ligands did not destabilize the enzymes (Table S2†).

RMSF analysis provided detailed insights into how the ligands influenced the mobility of specific regions within the protein structures. The protein–ligand complexes exhibited significantly lower RMSF values in regions directly involved in ligand binding, particularly amino acids in the active sites (Fig. 3B). In the case of AChE, reduced flexibility was observed in the catalytic triad region (Ser203, His447, Glu334), while for MAGL, the stabilizing effect was mainly concentrated in the triad residues (Ser122, His269, Asp239). The ligands exhibited a greater stabilizing effect on MAGL than on AChE, as evidenced by the lower average RMSF values in the complexes compared to the apoproteins.

Additional structural stability insights were obtained from the *R*_g_ and SASA analyses. The stable *R*_g_ values in the protein–ligand complexes indicated that the overall protein folding remained intact throughout the simulation. *R*_g_ fluctuated within the range of 2.324–2.329 nm for AChE and 1.843–1.853 nm for MAGL, confirming that the protein structures did not undergo expansion or collapse (Fig. 3C). Similarly, SASA remained stable, with minimal fluctuations (below 4 nm^2^ for AChE and below 2 nm^2^ for MAGL), suggesting that ligand binding did not significantly alter solvent exposure (Fig. 3D).

#### 2.4.2 Ligand stability

**Fig. 4:**
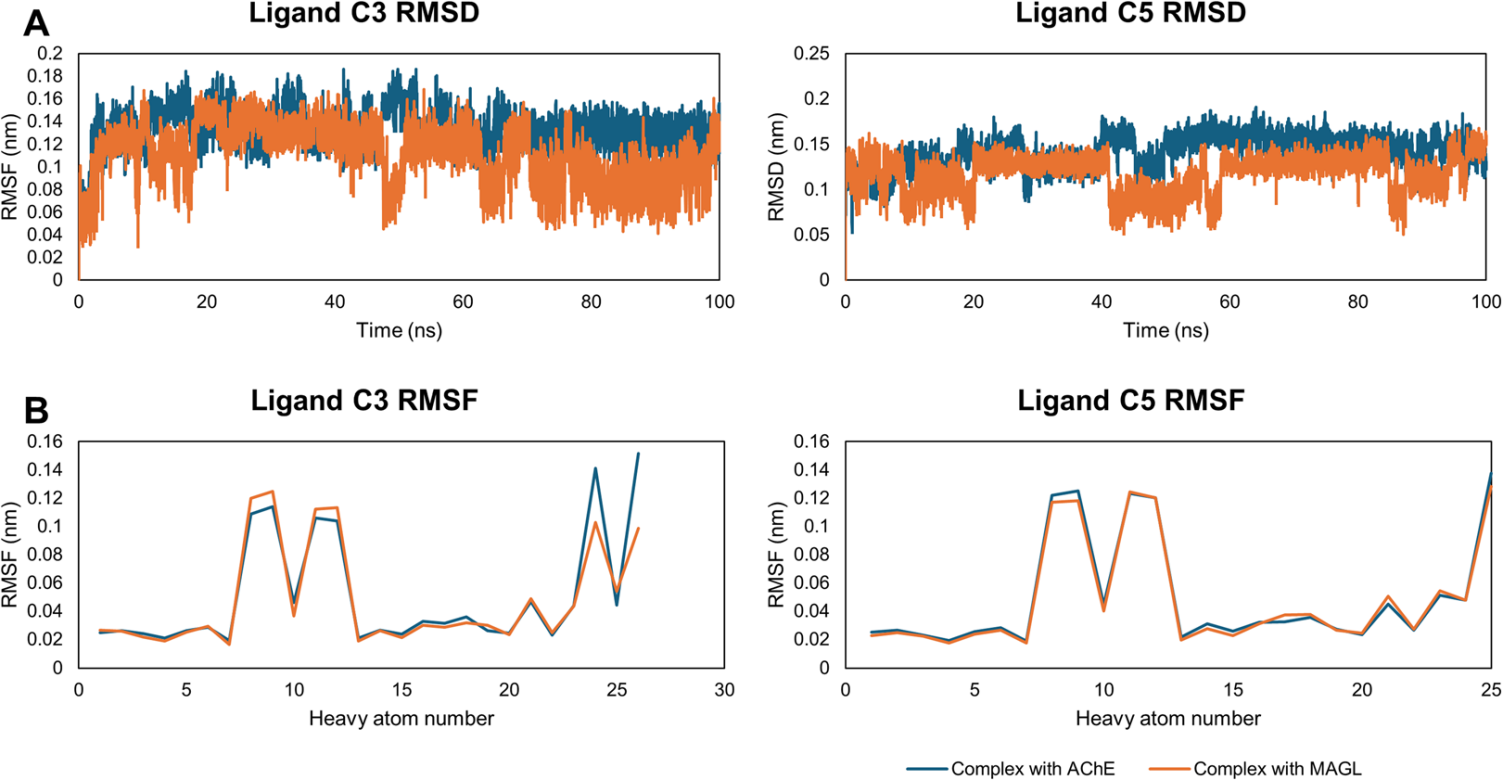
The stability of ligands C3 and C5 in complexes with AChE and MAGL was evaluated using RMSD plots (A) to assess structural deviations over time and RMSF calculations (B) to analyze the flexibility of heavy atoms.

The stability of ligands C3 and C5 within the binding pockets of AChE and MAGL was assessed through RMSD and RMSF analyses (Fig. 4A and B). After the initial equilibration phase, both C3 and C5 exhibited stable RMSD values with minimal fluctuations. The average RMSD values for C3 ranged around 0.134 nm (AChE) and 0.111 nm (MAGL), while C5 showed RMSD values of 0.141 nm (AChE) and 0.118 nm (MAGL) (Table S3†). These low fluctuations indicate that both ligands remained firmly positioned within the binding pockets without significant displacement or dissociation.

RMSF analysis provided insights into the atomic-level fluctuations of the ligands. The average RMSF values were below 0.051 nm, suggesting minimal atomic deviations, particularly for functional groups involved in enzymatic interactions. This result confirms that both ligands retained structural stability and did not undergo significant conformational changes during the simulation.

#### 2.4.3 Protein–ligand interactions

The MM/GBSA energy calculations and interaction network analysis provided profound insights into the molecular nature of protein–ligand interactions. MM/GBSA calculations over 100 ns of MD simulations revealed that both ligands, C3 and C5, tightly bound to AChE and MAGL, with binding free energies ranging from −21.19 to −29.16 kcal mol^−1^, confirming the thermodynamic stability of the complexes (Table 3). C5 exhibited the strongest affinity for MAGL, followed by MAGL-C3, AChE-C3, and AChE-C5 complexes.

**Table 3:** MM/GBSA binding free energy (kcal mol^−1^) of C3 and C5 with AChE and MAGL

Complex	VDWAALS^a^	EEL^b^	EGB^c^	ESURF^d^	GGAS^e^	GSOLV^f^	TOTAL^g^
AChE-C3	−41.06 ± 4.14	−5.70 ± 5.26	27.27 ± 3.39	−5.70 ± 0.39	−46.76 ± 8.13	21.56 ± 3.17	−25.19 ± 6.12
AChE-C5	−38.60 ± 3.34	−1.68 ± 7.32	24.50 ± 5.71	−5.41 ± 0.34	−40.28 ± 7.49	19.09 ± 5.56	−21.19 ± 3.52
MAGL-C3	−42.00 ± 2.88	−7.79 ± 11.21	28.58 ± 6.87	−6.14 ± 0.33	−49.79 ± 11.75	22.44 ± 6.69	−27.35 ± 5.79
MAGL-C5	−38.49 ± 3.50	−23.42 ± 9.98	38.60 ± 6.58	−5.85 ± 0.32	−61.91 ± 8.77	32.75 ± 6.56	−29.16 ± 3.90

a VDWAALS – van der Waals energy.

b EEL – electrostatic energy.

c EGB – polar solvation energy calculated using the Generalized Born model.

d ESURF – nonpolar solvation energy estimated based on the solvent-accessible surface area.

e GGAS – gas-phase interaction energy (sum of VDWAALS and EEL).

f GSOLV – solvation free energy (sum of EGB and ESURF).

g TOTAL – total binding free energy (sum of GGAS and GSOLV).

A detailed breakdown of MM/GBSA energy components revealed significant differences in the molecular binding mechanisms among the complexes. van der Waals interactions contributed significantly to the binding energy in all complexes, with values ranging from −38.49 to −42.00 kcal mol^−1^. In the AChE-C3 and MAGL-C3 complexes, van der Waals interactions played a dominant role in stabilizing the complexes, whereas the electrostatic contribution was relatively modest. This suggests that C3 binds to the enzymes primarily through strong hydrophobic interactions, especially with MAGL (−42.00 ± 2.88 kcal mol^−1^) and AChE (−41.06 ± 4.14 kcal mol^−1^).

Conversely, the MAGL-C5 complex exhibited a significant electrostatic contribution (−23.42 ± 9.98 kcal mol^−1^), much higher than that of the other complexes. Additionally, this complex had the highest solvation energy (GSOLV = EGB + ESURF) at 32.75 ± 6.56 kcal mol^−1^, reflecting the energy cost required to displace water molecules from polar groups of both the ligand and protein. However, the favorable electrostatic interactions were strong enough to offset this solvation penalty, resulting in the most favorable overall binding energy. This highlights the delicate balance between different molecular forces in protein–ligand binding and underscores the importance of optimizing both hydrophobic and hydrophilic interactions in drug design.

Results from initial molecular docking simulations revealed that both compounds, C3 and C5, exhibited strong affinity towards AChE and MAGL enzymes, with key interactions including hydrogen bonds and hydrophobic interactions. These analyses provide an overview of how the ligands interact with the proteins at their lowest energy states according to the docking model. However, results from 100 ns MD simulations highlighted significant changes in how the ligands maintained their interactions with the proteins under physiological conditions over time.

**Fig. 5:**
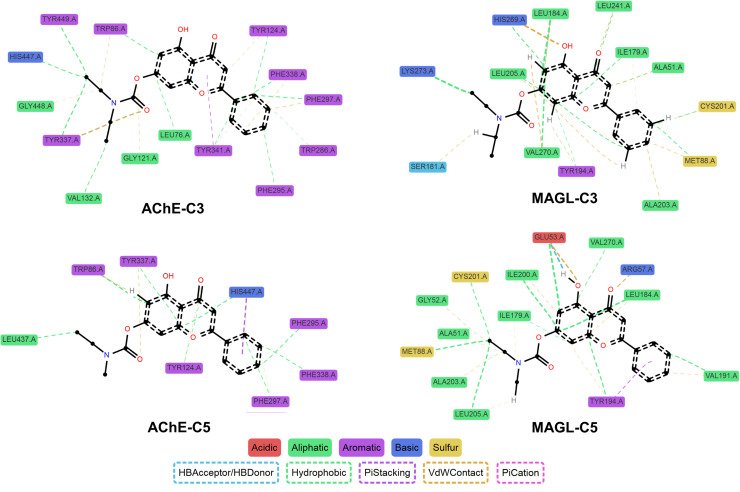
Ligand-enzyme interaction profiles of C3 and C5 from MD simulations, analyzed using ProLIF (occupancy >30%).

Data from ProLIF analysis showed that some interactions identified in docking were not consistently maintained throughout the MD simulation, particularly hydrogen bonds with Ser203 and His447 in AChE or Met123 in MAGL (Fig. 5 and Tables S4–S7†). This observation suggests that hydrogen bonding may be influenced by protein flexibility, solvent effects, and thermal fluctuations of the system. In contrast, hydrophobic interactions tended to be more stable. Key interactions observed in docking, such as those involving Trp86 and Tyr337 in AChE, as well as Ile179, Leu184, Leu205, and Val270 in MAGL, were preserved with a high frequency in MD simulations (>90%). This finding underscores the crucial role of hydrophobic residues in anchoring the ligand within the binding pocket.

Another notable aspect was the emergence of new interactions during the MD simulations. C3 formed a hydrogen bond with Tyr337 (AChE) at a frequency of 57%, while C5 established hydrogen bonds with Glu53 and Arg57 (MAGL) at frequencies of 78% and 37%, respectively. Additionally, the hydrogen bond interactions between the ligands and His447 (AChE) transitioned into hydrophobic interactions and π-stacking, suggesting that the ligand may undergo slight repositioning to optimize protein interactions. Newly identified hydrophobic interactions during MD simulations included Phe297 and Phe338 in AChE, which were present in both C3 and C5 complexes at a high frequency (>90%), emphasizing their role in ligand stability. For MAGL, stable interactions not predicted by docking were observed with Met88, His269, and Lys273 (C3 complex) as well as Glu53, Val191, and Ile200 (C5 complex). Additionally, multiple van der Waals interactions with surrounding amino acids remained stable, highlighting that not only strong interactions such as hydrogen bonding and hydrophobic contacts but also weaker interactions contribute to complex stability.

The differences between docking and MD simulations can be attributed to the rigid protein model used in docking, whereas MD simulations allow both the protein and ligand to adapt their conformations to achieve a more favorable energy state. This aspect is particularly critical in drug design, as ligand binding is not solely determined by the initial docking position but also by its ability to accommodate dynamic changes in the protein under physiological conditions.

Overall, MD simulation data confirmed the stability of ligand–protein complexes over time and provided additional insights into the flexibility of key interactions. The combination of both methods not only enhances our understanding of the binding mechanism but also lays the groundwork for optimizing ligand design, ensuring that observed interactions remain stable under biologically relevant conditions.

#### 2.4.4 Free energy landscape (FEL)

**Fig. 6:**
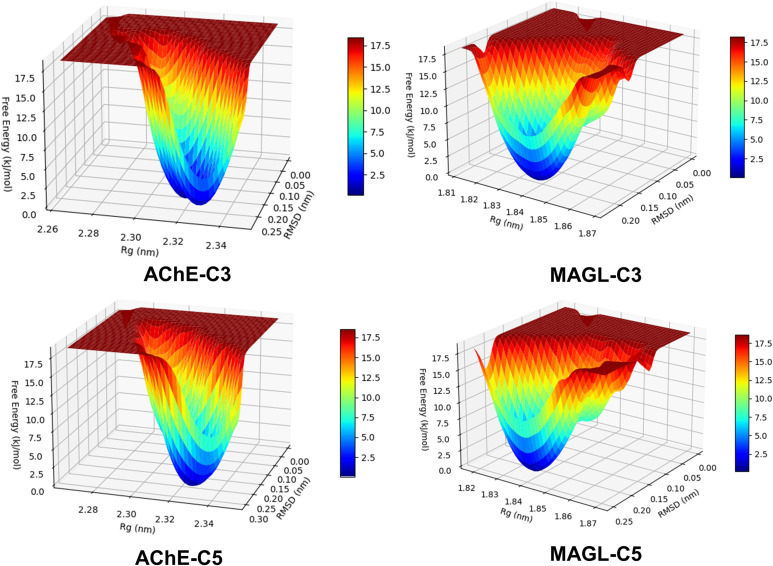
Three-dimensional FEL derived from RMSD and *R*_g_ for enzyme-ligand complexes in MD simulations.

The FEL of each enzyme-ligand complex was constructed to assess the conformational stability and structural dynamics over the 100 ns MD simulation. The FEL was constructed using RMSD and *R*_g_ as reaction coordinates, which provide critical insights into structural deviations and compactness, respectively.^34^ The energy landscape was derived from the probability distribution of these parameters using the Boltzmann inversion approach, and the resulting three-dimensional plots were visualized with a color gradient, where deep blue regions represent the lowest free energy states. The presence of a single deep energy well in all complexes suggests that each enzyme-ligand system maintains a predominant and thermodynamically favorable conformation with minimal structural fluctuations (Fig. 6). This characteristic indicates that the complexes reach a stable equilibrium state during the simulation, with limited transitions to higher-energy conformations. The absence of multiple shallow wells or fragmented basins further supports the notion that significant conformational rearrangements are unlikely to occur under these conditions. These findings confirm the structural stability of the studied complexes and suggest that the ligand binding interactions contribute to maintaining a well-defined and energetically favorable conformational state.

### 2.5 ADME predictions and drug-likeness

The ADME predictions of the 12 synthesized compounds revealed significant differences between chrysin and kaempferol derivatives regarding absorption, blood–brain barrier (BBB) permeability, interactions with *P*-glycoprotein (Pgp), and inhibition of CYP450 enzymes (Table 4). Overall, the chrysin carbamate derivatives exhibited high gastrointestinal (GI) absorption, whereas the kaempferol carbamate derivatives demonstrated low absorption. This distinction may impact the oral bioavailability and drug development potential of each compound class.

**Table 4:** Computationally predicted ADME and drug-likeness properties of compounds

Comp.	GI absorption	BBB permeant	Pgp substrate	CYP1A2 inhibitor	CYP2C19 inhibitor	CYP2C9 inhibitor	CYP2D6 inhibitor	CYP3A4 inhibitor	Lipinski violations	Bioavailability score
C1	High	No	No	Yes	No	Yes	No	No	0	0.55
C2	High	No	No	No	Yes	Yes	No	Yes	0	0.55
C3	High	No	No	Yes	Yes	Yes	No	Yes	0	0.55
C4	High	No	No	No	Yes	Yes	No	Yes	0	0.55
C5	High	No	No	Yes	Yes	Yes	No	Yes	0	0.55
C6	High	No	No	No	Yes	Yes	No	Yes	0	0.55
K1	Low	No	No	Yes	Yes	Yes	No	Yes	1	0.55
K2	Low	No	No	Yes	No	Yes	No	Yes	2	0.17
K3	Low	No	No	No	Yes	Yes	Yes	Yes	2	0.17
K4	Low	No	Yes	No	No	Yes	Yes	Yes	2	0.17
K5	Low	No	No	No	Yes	Yes	Yes	Yes	2	0.17
K6	Low	No	No	No	No	Yes	Yes	Yes	2	0.17

The inability of these compounds to cross the BBB may pose a limitation for AD treatment, as the pathology primarily affects the central nervous system. Future studies could explore strategies to enhance BBB permeability, such as molecular structure optimization to increase lipophilicity, prodrug design, or the use of targeted drug delivery systems.

Regarding interactions with the CYP450 metabolic enzyme system, chrysin carbamate derivatives exhibited distinct inhibition patterns, with each compound affecting different CYP450 isoenzymes, including CYP1A2, CYP2C19, CYP2C9, and CYP3A4, while showing no inhibition of CYP2D6. In contrast, kaempferol carbamate derivatives displayed heterogeneous CYP450 inhibition profiles, with most violating at least one of Lipinski's rules, thereby reducing their potential as orally available drug candidates.

Among the investigated compounds, C3 and C5 demonstrated the most potent inhibitory activity against both AChE and MAGL. These two compounds were predicted to have high GI absorption, BBB permeability, and not be substrates of Pgp. Notably, both C3 and C5 exhibited strong inhibition of CYP1A2, CYP2C19, CYP2C9, and CYP3A4, which could prolong their pharmacological effects but also warrants careful evaluation to prevent potential drug–drug interactions. More importantly, both compounds complied with Lipinski's rule of five, with an oral bioavailability score of 0.55, indicating promising drug-likeness properties. Compared to other derivatives in the series, C3 and C5 possess significant advantages in terms of drug compatibility and pharmacokinetic characteristics.

## 3. Conclusions

This study focused on the design, synthesis, and biological evaluation of flavonoid carbamate derivatives with the aim of developing multi-target inhibitors for AD. Through the synthetic process, we successfully obtained 12 carbamate derivatives from chrysin and kaempferol, with high yields (53–79%), and their structures were confirmed using modern spectroscopic techniques, including NMR, HRMS, and UV.

*In vitro* assays demonstrated that several compounds exhibited inhibitory activity against both MAGL and AChE enzymes. Notably, compounds C3 and C5 displayed remarkable dual-target inhibition, with IC_50_ values of 22.86 μM for MAGL and 61.78 μM for AChE (C3), and 46.65 μM for MAGL and 89.40 μM for AChE (C5). These findings suggest that these compounds may serve as dual inhibitors, an essential strategy in AD treatment.

Molecular docking and MD simulations further provided insights into the binding affinity of these compounds with the target enzymes. The results indicated that the carbamate group plays a crucial role in enhancing interactions with key amino acid residues in the active sites of AChE and MAGL. Both C3 and C5 exhibited stable interactions within the enzyme environment, with favorable MM/GBSA binding free energy, particularly for MAGL. ADME predictions revealed that chrysin carbamate derivatives demonstrated better GI absorption compared to their kaempferol-based counterparts.

Overall, this study identified promising lead compounds, particularly C3 and C5, which could serve as potential multi-target inhibitors for AD. Future research should focus on structural optimization to enhance selectivity, pharmacokinetic properties, and *in vivo* investigations, paving the way for the clinical application of these hybrid compounds.

## 4. Experimental

### 4.1 Materials and equipment

#### 4.1.1 Chemicals and reagents

All chemicals and reagents utilized in this study were obtained from reputable suppliers and used without further purification. Chrysin (98%) and kaempferol (98%) were sourced from Shaanxi (China). The carbamoyl chloride derivatives, including *N*,*N*-dimethylcarbamoyl chloride (97%), *N*,*N*-diethylcarbamoyl chloride (98%), and *N*-ethyl-*N*-methylcarbamoyl chloride (99%), were purchased from Macklin (China).

For the enzymatic inhibition assays, AChE enzyme, acetylthiocholine iodide, and 5,5′-dithiobis-2-nitrobenzoic acid were obtained from Sigma-Aldrich (Germany). The MAGL enzyme was purchased from Finetest (China), while 4-nitrophenyl acetate (4-NPA), used as a surrogate substrate, was sourced from Sigma-Aldrich (Germany).

#### 4.1.2 Equipment and instruments

##### 4.1.2.1 Synthetic procedures

Thin-layer chromatography was performed on silica gel 60 F_254_ plates (Merck, Germany). A magnetic stirrer with heating (C-MAG HS7 Digital, Ika, Germany) and a rotary evaporator (R-100, Büchi, Switzerland) were used for reaction processing and solvent removal. The synthesized compounds were characterized using UV spectroscopy (V-730, Jasco, Japan), HRMS (QTOF X500, Sciex, USA), NMR spectroscopy (Bruker AM500 FT-NMR and Advance NEO 600 MHz, Bruker, Germany), and melting point determination (Stuart SMP20, Cole-Parmer, UK).

##### 4.1.2.2 Bioassay procedures

Enzymatic inhibition assays were conducted in a 96-well microplate format, with absorbance recorded using an ELISA reader (EMR-500, Labomed, USA). Supporting equipment included micropipettes (C50-8, CAPP, Denmark), a vortex mixer (VX-200, Labnet, USA), an ultrasonic bath (S100H, Elmasonic, Germany), and a pH meter (sensION+ PH3, Hach, USA).

##### 4.1.2.3 Computational studies

*In silico* molecular docking and MD simulations were performed using a high-performance computing system equipped with an Intel Core i5-12400F processor and an NVIDIA RTX 4060 8 GB GPU, ensuring efficient processing of complex calculations.

### 4.2 Synthesis

#### 4.2.1 Synthesis procedure

A total of 5 mmol of the flavonoid (chrysin or kaempferol) was weighed and added to a two-neck round-bottom flask, followed by the addition of 250 mL of anhydrous acetone. The mixture was stirred until complete dissolution. K_2_CO_3_ was then added, and stirring was continued. Subsequently, the corresponding carbamoyl chloride reagent (*N*,*N*-diethylcarbamoyl chloride, *N*-ethyl-*N*-methylcarbamoyl chloride, or *N*,*N*-dimethylcarbamoyl chloride) was introduced into the reaction flask. The reaction mixture was stirred and heated at 55 °C under reflux, with progress monitored by thin-layer chromatography using a suitable solvent system. Upon completion, the reaction mixture was filtered to remove insoluble solids, and the filtrate was concentrated under reduced pressure to obtain the crude product. The crude product was then purified by recrystallization using an appropriate solvent system.

The specific amounts of K_2_CO_3_ and carbamoyl chloride used for the synthesis of each compound are provided in Tables S8 and S9 in the ESI.†

#### 4.2.2 5-Hydroxy-4-oxo-2-phenyl-4*H*-chromen-7-yl dimethylcarbamate (C1)

Yellow crystals; yield: 66%; mp: 157 °C; UV (*λ*_max_ nm, MeOH): 210, 216, 268, 341; ^1^H NMR (600 MHz, DMSO-*d*_6_): *δ* 12.79 (s, 1H, OH), 8.11–8.09 (m, 2H, Ar–H), 7.63–7.62 (m, 1H, Ar–H), 7.59 (dd, *J* = 8.3, 6.6 Hz, 2H, Ar–H), 7.11 (s, 1H, Ar–H), 7.08 (d, *J* = 1.8 Hz, 1H, Ar–H), 6.65 (d, *J* = 1.8 Hz, 1H, Ar–H), 3.06 (s, 3H, NCH_3_), 2.94 (s, 3H, NCH_3_); ^13^C NMR (150 MHz, DMSO-*d*_6_): *δ* 182.4 (C=O), 164.0, 160.5, 156.8, 156.1, 152.7, 132.2, 130.3, 129.1, 126.5, 107.6, 105.5, 104.9, 101.1, 36.3 (NCH_3_), 36.1 (NCH_3_); HRMS (ESI): calcd. for C_18_H_16_NO_5_ [M + H]^+^: 326.1028, found: 326.1014.

#### 4.2.3 4-Oxo-2-phenyl-4*H*-chromene-5,7-diyl bis(dimethylcarbamate) (C2)

White crystals; yield: 78%; mp: 193 °C; UV (*λ*_max_ nm, MeOH): 209, 218, 255, 296; ^1^H NMR (600 MHz, DMSO-*d*_6_): *δ* 8.06–8.05 (m, 2H, Ar–H), 7.60–7.56 (m, 3H, Ar–H), 7.53 (d, *J* = 2.4 Hz, 1H, Ar–H), 7.02 (d, *J* = 2.4 Hz, 1H, Ar–H), 6.86 (s, 1H, Ar–H), 3.10 (s, 3H, NCH_3_), 3.07 (s, 3H, NCH_3_), 2.95 (s, 3H, NCH_3_), 2.92 (s, 3H, NCH_3_); ^13^C NMR (150 MHz, DMSO-*d*_6_): *δ* 175.5 (C=O), 161.3, 156.8, 154.6, 153.6, 152.6, 150.3, 131.7, 130.5, 129.0, 126.1, 114.5, 114.1, 108.5, 107.8, 36.4 (NCH_3_), 36.3 (NCH_3_), 36.1 (NCH_3_); HRMS (ESI): calcd. for C_21_H_21_N_2_O_6_ [M + H]^+^: 397.1400, found: 397.1381.

#### 4.2.4 5-Hydroxy-4-oxo-2-phenyl-4*H*-chromen-7-yl diethylcarbamate (C3)

Yellow crystals; yield: 65%; mp: 136 °C; UV (*λ*_max_ nm, MeOH): 209, 218, 268; ^1^H NMR (600 MHz, DMSO-*d*_6_): *δ* 12.79 (s, 1H, OH), 8.11–8.10 (m, 2H, Ar–H), 7.63–7.57 (m, 3H, Ar–H), 7.09 (s, 1H, Ar–H), 7.07 (d, *J* = 2.4 Hz, 1H, Ar–H), 6.63 (d, *J* = 2.4 Hz, 1H, Ar–H), 3.42–3.40 (m, 2H, NCH_2_), 3.34–3.32 (m, 2H, NCH_2_), 1.21 (t, *J* = 6.6 Hz, 3H, CH_3_), 1.15 (t, *J* = 6.6 Hz, 3H, CH_3_); ^13^C NMR (150 MHz, DMSO-*d*_6_): *δ* 182.4 (C=O), 163.9, 160.5, 156.8, 156.1, 152.0, 132.2, 130.3, 129.0, 126.5, 107.5, 105.5, 104.8, 101.0, 41.9 (NCH_2_), 41.6 (NCH_2_), 14.0 (CH_3_), 13.1 (CH_3_); HRMS (ESI): calcd. for C_20_H_20_NO_5_ [M + H]^+^: 354.1341, found: 354.1329.

#### 4.2.5 4-Oxo-2-phenyl-4*H*-chromene-5,7-diyl bis(diethylcarbamate) (C4)

White crystals; yield: 76%; mp: 114 °C; UV (*λ*_max_ nm, MeOH): 219, 255, 295; ^1^H NMR (600 MHz, DMSO-*d*_6_): *δ* 8.08–8.07 (m, 2H, Ar–H), 7.60–7.56 (m, 3H, Ar–H), 7.55 (d, *J* = 2.4 Hz, 1H, Ar–H), 7.04 (d, *J* = 2.4 Hz, 1H, Ar–H), 6.88 (s, 1H, Ar–H), 3.47 (q, *J* = 7.2 Hz, 2H, NCH_2_), 3.43 (q, *J* = 6.6 Hz, 2H, NCH_2_), 3.34 (q, *J* = 6.6 Hz, 2H, NCH_2_), 3.29 (q, *J* = 7.2 Hz, 2H, NCH_2_), 1.27 (t, *J* = 6.6 Hz, 3H, CH_3_), 1.22 (t, *J* = 6.6 Hz, 3H, CH_3_), 1.15 (t, *J* = 7.2 Hz, 3H, CH_3_), 1.14 (t, *J* = 7.2 Hz, 3H, CH_3_); ^13^C NMR (150 MHz, DMSO-*d*_6_): *δ* 175.4 (C=O), 161.2, 156.8, 154.5, 152.7, 152.0, 150.3, 131.7, 130.6, 129.0, 126.1, 114.7, 114.3, 108.5, 107.8, 41.9 (NCH_2_), 41.6 (NCH_2_), 41.5 (NCH_2_), 41.4 (NCH_2_), 14.0 (CH_3_), 13.7 (CH_3_), 13.1 (CH_3_), 13.1 (CH_3_); HRMS (ESI): calcd. for C_25_H_29_N_2_O_6_ [M + H]^+^: 453.2026, found: 453.1999.

#### 4.2.6 5-Hydroxy-4-oxo-2-phenyl-4*H*-chromen-7-yl ethyl(methyl)carbamate (C5)

Yellow crystals; yield: 65%; mp: 101 °C; UV (*λ*_max_ nm, MeOH): 209, 218, 268; ^1^H NMR (600 MHz, DMSO-*d*_6_): *δ* 12.79 (s, 1H, OH), 8.10 (d, *J* = 7.8 Hz, 2H, Ar–H), 7.64–7.57 (m, 3H, Ar–H), 7.10 (s, 1H, Ar–H), 7.08 (d, *J* = 1.8 Hz, 1H, Ar–H), 6.64 (d, *J* = 1.8 Hz, 1H, Ar–H), 3.43 (q, *J* = 7.2 Hz, 1H, 1/2NCH_2_), 3.35 (q, *J* = 7.2 Hz, 1H, 1/2NCH_2_), 3.04 (s, 1.5H, 1/2NCH_3_), 2.93 (s, 1.5H, 1/2NCH_3_), 1.20 (t, *J* = 7.2 Hz, 1.5H, 1/2CH_3_), 1.13 (t, *J* = 7.2 Hz, 1.5H, 1/2CH_3_); ^13^C NMR (150 MHz, DMSO-*d*_6_): *δ* 182.4 (C=O), 164.0, 160.5, 156.8, 156.1, 152.2, 132.2, 130.3, 129.1, 126.5, 107.6, 105.5, 104.9, 101.1, 43.6 (1/2NCH_2_), 43.4 (1/2NCH_2_), 33.9 (1/2NCH_3_), 33.6 (1/2NCH_3_), 12.9 (1/2CH_3_), 12.1 (1/2CH_3_); HRMS (ESI): calcd. for C_19_H_18_NO_5_ [M + H]^+^: 340.1185, found: 340.1166.

#### 4.2.7 4-Oxo-2-phenyl-4*H*-chromene-5,7-diyl bis(ethyl(methyl)carbamate) (C6)

White crystals; yield: 79%; mp: 127 °C; UV (*λ*_max_ nm, MeOH): 209, 218, 255, 296; ^1^H NMR (600 MHz, DMSO-*d*_6_): *δ* 8.06 (d, *J* = 7.8 Hz, 2H, Ar–H), 7.60–7.56 (m, 3H, Ar–H), 7.54 (d, *J* = 10.8 Hz, 1H, Ar–H), 7.02 (s, 1H, Ar–H), 6.87 (d, *J* = 10.8 Hz, 1H, Ar–H), 3.48 (q, *J* = 6.6 Hz, 1H, 1/2NCH_2_), 3.45 (q, *J* = 6.6 Hz, 1H, 1/2NCH_2_), 3.35 (q, *J* = 6.6 Hz, 1H, 1/2NCH_2_), 3.32 (q, *J* = 6.6 Hz, 1H, 1/2NCH_2_), 3.07 (s, 1.5H, 1/2NCH_3_), 3.05 (s, 1.5H, 1/2NCH_3_), 2.94 (s, 1.5H, 1/2NCH_3_), 2.90 (s, 1.5H, 1/2NCH_3_), 1.27 (t, *J* = 6.6 Hz, 1.5H, 1/2CH_3_), 1.21 (t, *J* = 6.6 Hz, 1.5H, 1/2CH_3_), 1.14 (t, *J* = 6.6 Hz, 3H, CH_3_); ^13^C NMR (150 MHz, DMSO-*d*_6_): *δ* 175.5 (C=O), 161.2, 156.8, 154.5, 153.1, 152.3, 150.3, 131.7, 130.5, 129.0, 126.1, 114.6, 114.2, 108.5, 107.8, 43.6 (NCH_2_), 43.5 (NCH_2_), 33.9 (1/2NCH_3_), 33.8 (1/2NCH_3_), 33.7 (1/2NCH_3_), 33.6 (1/2NCH_3_), 12.9 (1/2CH_3_), 12.5 (1/2CH_3_), 12.1 (1/2CH_3_), 12.0 (1/2CH_3_); HRMS (ESI): calcd. for C_23_H_25_N_2_O_6_ [M + H]^+^: 425.1713, found: 425.1687.

#### 4.2.8 2-(4-((Dimethylcarbamoyl)oxy)phenyl)-5-hydroxy-4-oxo-4*H*-chromene-3,7-diyl bis(dimethylcarbamate) (K1)

Yellow crystals; yield: 69%; mp: 203 °C; UV (*λ*_max_ nm, MeOH): 218, 266, 299; ^1^H NMR (600 MHz, DMSO-*d*_6_): *δ* 12.11 (s, 1H, OH), 7.94 (dd, *J* = 8.4, 1.8 Hz, 2H, Ar–H), 7.38 (dd, *J* = 8.4, 1.8 Hz, 2H, Ar–H), 7.10 (d, *J* = 1.8 Hz, 1H, Ar–H), 6.71 (d, *J* = 1.8 Hz, 1H, Ar–H), 3.09 (s, 3H, NCH_3_), 3.07 (s, 3H, NCH_3_), 3.05 (s, 3H, NCH_3_), 2.94 (s, 6H, 2NCH_3_), 2.90 (s, 3H, NCH_3_); ^13^C NMR (125 MHz, DMSO-*d*_6_): *δ* 176.4 (C=O), 160.1, 157.1, 156.3, 155.5, 153.8, 153.3, 152.6, 152.3, 131.7, 129.5, 125.6, 122.4, 107.5, 105.0, 101.4, 36.5 (NCH_3_), 36.4 (NCH_3_), 36.3 (NCH_3_), 36.2 (NCH_3_), 36.2 (NCH_3_), 36.1 (NCH_3_); HRMS (ESI): calcd. for C_24_H_26_N_3_O_9_ [M + H]^+^: 500.1669, found: 500.1640.

#### 4.2.9 2-(4-((Dimethylcarbamoyl)oxy)phenyl)-4-oxo-4*H*-chromene-3,5,7-triyl tris(dimethylcarbamate) (K2)

White crystals; yield: 77%; mp: 158 °C; UV (*λ*_max_ nm, MeOH): 209, 219, 250, 303; ^1^H NMR (600 MHz, DMSO-*d*_6_): *δ* 7.92 (dd, *J* = 7.2, 1.8 Hz, 2H, Ar–H), 7.54 (d, *J* = 1.8 Hz, 1H, Ar–H), 7.36 (dd, *J* = 7.2, 1.8 Hz, 2H, Ar–H), 7.09 (d, *J* = 1.8 Hz, 1H, Ar–H), 3.30 (s, 3H, NCH_3_), 3.10 (s, 3H, NCH_3_), 3.07 (s, 3H, NCH_3_), 3.06 (s, 3H, NCH_3_), 2.94 (s, 3H, NCH_3_), 2.93 (s, 3H, NCH_3_), 2.92 (s, 3H, NCH_3_), 2.88 (s, 3H, NCH_3_); ^13^C NMR (125 MHz, DMSO-*d*_6_): *δ* 170.2 (C=O), 156.1, 154.9, 154.2, 153.5, 153.4, 153.4, 152.6, 152.4, 150.4, 133.3, 129.3, 125.9, 122.3, 114.6, 114.3, 108.6, 36.5 (NCH_3_), 36.4 (NCH_3_), 36.3 (NCH_3_), 36.2 (NCH_3_), 36.1 (NCH_3_); HRMS (ESI): calcd. for C_27_H_31_N_4_O_10_ [M + H]^+^: 571.2040, found: 571.2008.

#### 4.2.10 2-(4-((Diethylcarbamoyl)oxy)phenyl)-5-hydroxy-4-oxo-4*H*-chromene-3,7-diyl bis(diethylcarbamate) (K3)

Yellow crystals; yield: 58%; mp: 162 °C; UV (*λ*_max_ nm, MeOH): 209, 217, 266; ^1^H NMR (600 MHz, DMSO-*d*_6_): *δ* 12.12 (s, 1H, OH), 7.94–7.91 (m, 2H, Ar–H), 7.39–7.37 (m, 2H, Ar–H), 7.11 (d, *J* = 2.4 Hz, 1H, Ar–H), 6.71 (d, *J* = 2.4 Hz, 1H, Ar–H), 3.46–3.40 (m, 6H, 3NCH_2_), 3.33–3.29 (m, 6H, 3NCH_2_), 1.21–1.18 (m, 9H, 3CH_3_), 1.15–1.13 (m, 6H, 2CH_3_), 1.09 (t, *J* = 7.2 Hz, 3H, CH_3_); ^13^C NMR (125 MHz, DMSO-*d*_6_): *δ* 176.5 (C=O), 160.2, 157.1, 156.3, 155.5, 153.7, 152.6, 151.9, 151.7, 131.5, 129.5, 125.6, 122.3, 107.5, 104.9, 101.3, 42.0 (NCH_2_), 41.9 (NCH_2_), 41.8 (NCH_2_), 41.7 (NCH_2_), 41.6 (NCH_2_), 41.5 (NCH_2_), 14.1 (CH_3_), 14.0 (CH_3_), 13.2 (CH_3_), 13.1 (CH_3_); HRMS (ESI): calcd. for C_30_H_38_N_3_O_9_ [M + H]^+^: 584.2608, found: 584.2587.

#### 4.2.11 2-(4-((Diethylcarbamoyl)oxy)phenyl)-4-oxo-4*H*-chromene-3,5,7-triyl tris(diethylcarbamate) (K4)

White crystals; yield: 78%; mp: 150 °C; UV (*λ*_max_ nm, MeOH): 209, 219, 250, 301; ^1^H NMR (600 MHz, DMSO-*d*_6_): *δ* 7.92 (dd, *J* = 7.2, 1.8 Hz, 2H, Ar–H), 7.54 (d, *J* = 1.8 Hz, 1H, Ar–H), 7.36 (dd, *J* = 7.2, 1.8 Hz, 2H, Ar–H), 7.09 (d, *J* = 1.8 Hz, 1H, Ar–H), 3.48–3.41 (m, 8H, 4NCH_2_), 3.35–3.25 (m, 8H, 4NCH_2_), 1.25 (t, *J* = 7.2 Hz, 3H, CH_3_), 1.22–1.11 (m, 18H, 6CH_3_), 1.08 (t, *J* = 7.2 Hz, 3H, CH_3_); ^13^C NMR (150 MHz, DMSO-*d*_6_): *δ* 170.1 (C=O), 156.1, 154.7, 153.8, 153.4, 152.6, 152.6, 151.9, 151.8, 150.4, 133.5, 129.1, 125.9, 122.1, 114.3, 114.3, 108.4, 41.9 (NCH_2_), 41.8 (NCH_2_), 41.7 (NCH_2_), 41.6 (NCH_2_), 41.5 (NCH_2_), 14.5 (CH_3_), 13.8 (CH_3_), 13.6 (CH_3_), 13.1 (CH_3_), 13.0 (CH_3_), 12.9 (CH_3_), 12.9 (CH_3_); HRMS (ESI): calcd. for C_35_H_47_N_4_O_10_ [M + H]^+^: 683.3292, found: 683.3256.

#### 4.2.12 2-(4-((Ethyl(methyl)carbamoyl)oxy)phenyl)-5-hydroxy-4-oxo-4*H*-chromene-3,7-diyl bis(ethyl(methyl)carbamate) (K5)

Yellow crystals; yield: 53%; mp: 153 °C; UV (*λ*_max_ nm, MeOH): 209, 218, 266, 298, 333; ^1^H NMR (600 MHz, DMSO-*d*_6_): *δ* 12.11 (s, 1H, OH), 7.95–7.92 (m, 2H, Ar–H), 7.39–7.37 (m, 2H, Ar–H), 7.11 (d, *J* = 1.8 Hz, 1H, Ar–H), 6.71 (d, *J* = 1.8 Hz, 1H, Ar–H), 3.47–3.42 (m, 3H, 3/2NCH_2_), 3.35–3.30 (m, 3H, 3/2NCH_2_), 3.06 (s, 1.5H, 1/2NCH_3_), 3.04 (s, 1.5H, 1/2NCH_3_), 3.03 (s, 1.5H, 1/2NCH_3_), 2.93 (s, 1.5H, 1/2NCH_3_), 2.92 (s, 1.5H, 1/2NCH_3_), 2.89 (s, 1.5H, 1/2NCH_3_), 1.21–1.17 (m, 4.5H, 3/2CH_3_), 1.13 (t, *J* = 7.2 Hz, 1.5H, 1/2CH_3_), 1.12 (t, *J* = 7.2 Hz, 1.5H, 1/2CH_3_), 1.07 (t, *J* = 7.2 Hz, 1.5H, 1/2CH_3_); ^13^C NMR (125 MHz, DMSO-*d*_6_): *δ* 176.5 (C=O), 160.1, 157.1, 156.4, 155.5, 153.8, 152.0, 131.8, 129.5, 125.6, 122.3, 107.5, 105.0, 101.4, 43.7 (NCH_2_), 43.6 (NCH_2_), 43.5 (NCH_2_), 34.1 (NCH_3_), 34.0 (NCH_3_), 33.7 (NCH_3_), 13.0 (CH_3_), 12.9 (CH_3_), 12.1 (CH_3_); HRMS (ESI): calcd. for C_27_H_32_N_3_O_9_ [M + H]^+^: 542.2139, found: 542.2110.

#### 4.2.13 2-(4-((Ethyl(methyl)carbamoyl)oxy)phenyl)-4-oxo-4*H*-chromene-3,5,7-triyl tris(ethyl(methyl)carbamate) (K6)

White crystals; yield: 71%; mp: 132 °C; UV (*λ*_max_ nm, MeOH): 209, 219, 250, 301; ^1^H NMR (600 MHz, DMSO-*d*_6_): *δ* 7.92–7.90 (m, 2H, Ar–H), 7.55 (d, *J* = 1.2 Hz, 1H, Ar–H), 7.36–7.35 (m, 2H, Ar–H), 7.09 (d, *J* = 1.2 Hz, 1H, Ar–H), 3.48–3.43 (m, 4H, 2NCH_2_), 3.35–3.30 (m, 4H, 2NCH_2_), 3.07 (s, 1.5H, 1/2NCH_3_), 3.05 (s, 3H, NCH_3_), 3.03 (s, 1.5H, 1/2NCH_3_), 2.93 (s, 1.5H, 1/2NCH_3_), 2.92 (s, 1.5H, 1/2NCH_3_), 2.90 (s, 1.5H, 1/2NCH_3_), 2.86 (s, 1.5H, 1/2NCH_3_), 1.24 (t, *J* = 7.2 Hz, 1.5H, 1/2CH_3_), 1.21 (t, *J* = 7.2 Hz, 3H, CH_3_), 1.16–1.11 (m, 6H, 2CH_3_), 1.05 (t, *J* = 7.2 Hz, 1.5H, 1/2CH_3_); ^13^C NMR (150 MHz, DMSO-*d*_6_): *δ* 170.1 (C=O), 156.1, 154.8, 153.4, 152.9, 152.1, 150.0, 129.2, 129.2, 125.9, 122.2, 114.4, 113.8, 108.6, 108.5, 43.6 (NCH_2_), 43.2 (NCH_2_), 34.0 (NCH_3_), 33.8 (NCH_3_), 33.6 (NCH_3_), 33.5 (NCH_3_), 12.9 (CH_3_), 12.1 (CH_3_), 12.1 (CH_3_), 12.0 (CH_3_); HRMS (ESI): calcd. for C_31_H_39_N_4_O_10_ [M + H]^+^: 627.2666, found: 627.2632.

### 4.3 Bioactivity evaluation

#### 4.3.1 AChE inhibition assay

The AChE inhibitory activity of the synthesized compounds was determined using Ellman's method, with slight modifications to optimize experimental conditions.^35^ The assay was performed following the procedures described in our previous study.^36^

#### 4.3.2 MAGL inhibition assay

The MAGL inhibitory activity of the synthesized compounds was assessed using a spectrophotometric assay, employing 4-NPA as a surrogate substrate. The experimental procedure was adapted from a previously published protocol with minor modifications.^37^

The assay was conducted at room temperature using an ELISA reader (EMR-500) with a 96-well plate, with a total reaction volume of 200 μL per well. The reaction system comprised 10 mM tris–HCl buffer (pH 7.2), 1 mM EDTA, and 0.1 mg mL^−1^ bovine serum albumin. A volume of 150 μL of 4-NPA solution (133.3 μM, final concentration: 100 μM) was added to each well, followed by 10 μL of the test compound or the reference inhibitor JZL-184 (dissolved in DMSO at various concentrations). The negative control was prepared by replacing the inhibitor with 10 μL of DMSO. The enzymatic reaction was initiated by adding 40 μL of MAGL enzyme solution (11 ng per well), while the blank control used buffer instead of the enzyme. The reaction mixtures were incubated for 30 minutes, and absorbance was measured at 405 nm. Each sample was tested in triplicate, and IC_50_ values were determined from the obtained data using GraphPad Prism 8.4.3.

### 4.4 Molecular docking method

Molecular docking studies were conducted using AutoDock Vina version 1.1.2 to predict the binding affinity and interactions of the synthesized flavonoid carbamate derivatives with target enzymes.^38^ The structures of AChE (PDB: 4EY6) and MAGL (PDB: 3PE6) were retrieved from the Protein Data Bank and prepared using AutoDock Tools version 1.5.7.^39–41^ The preparation steps included adding hydrogen atoms, removing water molecules, and assigning Kollman charges. Ligands were energy-minimized using the MM94FF force field in Open Babel version 3.1.1 before docking.^42^ The docking grid was defined based on the active site of each protein. After docking, the binding poses with the lowest binding energy were selected for molecular interaction analysis, including hydrogen bonding and hydrophobic interactions, using UCSF ChimeraX version 1.9 and Discovery Studio version 2024.1.^43^

### 4.5 Molecular dynamics simulation method

MD simulations were performed using GROMACS 2024.3 to evaluate the stability and interactions of the protein–ligand complexes, employing the CHARMM36 force field for both proteins and ligands.^44–46^ The apoproteins (AChE and MAGL) and their complexes with the most potent enzyme inhibitors (C3 and C5) were selected for simulation. Protein structures were prepared using UCSF Chimera 1.18.^47^ Ligand topology and force field parameters were generated using SwissParam, which provides CHARMM-compatible parameters for small molecules, allowing seamless integration with the CHARMM36 force field in GROMACS.^48,49^

The system was placed in a triclinic simulation box, solvated with TIP3P water molecules, and neutralized by adding Na^+^ and Cl^−^ ions at a concentration of 0.15 M. After energy minimization to eliminate steric clashes, the system was equilibrated in two phases: NVT (temperature stabilization at 300 K) and NPT (pressure stabilization at 1 atm), each lasting 100 ps. Subsequently, a 100 ns MD simulation was conducted at 300 K and 1 atm using the leap-frog algorithm, with bond constraints applied *via* the LINCS method.

Key dynamical parameters, RMSD, RMSF, SASA, and *R*_g_, were analyzed to assess the stability and interaction capability of the protein–ligand complexes. To evaluate ligand binding affinity, the MM/GBSA method was employed to calculate the free binding energy from the MD simulation trajectory, with data extracted every 10 frames.^50^ Additionally, the ProLIF tool was used to analyze key interactions, including hydrogen bonding, hydrophobic interactions, and π-stacking between the ligands and enzymes throughout the MD simulation.^51^

### 4.6 Construction of the FEL

The FEL was constructed from MD simulation data to evaluate the stability and conformational dynamics of enzyme-ligand complexes. The RMSD and *R*_g_ were chosen as reaction coordinates to describe the structural flexibility and compactness of the complex.^52,53^ The data were discretized into a 40 × 40 bin grid, and the probability distribution of each state was estimated using a histogram method.

The Gibbs free energy (*G*) was calculated using the Boltzmann equation:*G* = −*RT *ln(*P*)where *R* is the gas constant, *T* is the temperature, and *P* is the probability of a given state.

A 3D plot was generated using Matplotlib, with the “jet” colormap representing energy variations.

### 4.7 Prediction of ADME and drug-likeness

The absorption, distribution, metabolism, and excretion (ADME) properties and drug-likeness of the synthesized compounds, were predicted using the online tool SwissADME (https://www.swissadme.ch/index.php).^54^

## Data availability

The data supporting this article have been included as part of the ESI.†

## Author contributions

The-Huan Tran: conceptualization, investigation, formal analysis, methodology, funding acquisition, software, visualization, writing – original draft. Dai-Nhat-Huy Doan: investigation. Thi-Cam-Nhung Cao: investigation. Thai-Son Tran: investigation, software. Thanh-Dao Tran: conceptualization, methodology, supervision, writing – review & editing.

## Conflicts of interest

There are no conflicts to declare.

## Supplementary Material

RA-015-D5RA02267C-s001
