# Supplementary material for: Flavonoid carbamate hybrids: design, synthesis, and evaluation as multi-target enzyme inhibitors for Alzheimer's disease
Source: RSC Adv. 2025 May 20;15(21):16855–68. doi: 10.1039/d5ra02267c (PMC12090193; doi:10.1039/d5ra02267c)
Supplement: RA-015-D5RA02267C-s001 [file RA-015-D5RA02267C-s001.pdf]

Supplementary Information (SI) for RSC Advances.
This journal is © The Royal Society of Chemistry 2025

Supplementary information

Flavonoid carbamate hybrids: design, synthesis, and evaluation as multi-target enzyme inhibitors for Alzheimer's disease

The-Huan Tran,[a,b] Dai-Nhat-Huy Doan,[b] Thi-Cam-Nhung Cao,[b] Thai-Son Tran[b] and Thanh-Dao Tran*[a]

[a]*Faculty of Pharmacy, University of Medicine and Pharmacy at Ho Chi Minh City, Ho Chi Minh 700000, Vietnam*

[b]*Faculty of Pharmacy, University of Medicine and Pharmacy, Hue University, Hue 530000, Vietnam*

Corresponding author:
Thanh-Dao Tran
Faculty of Pharmacy, University of Medicine and Pharmacy at Ho Chi Minh City, Ho Chi Minh 700000, Vietnam
Email: daott@ump.edu.vn

Table of Contents	Page
**Table S1.** Docking results between carbamate derivatives and enzymes	1
**Table S2.** Stability parameters of enzymes in the apoprotein state and ligand-bound complexes from MD simulations	2
**Table S3.** Stability parameters of ligands C3 and C5 in enzyme-ligand complexes	2
**Table S4.** Interaction analysis between AChE and C3 based on molecular dynamics simulation using ProLIF (occupancy >30%)	3
**Table S5.** Interaction analysis between AChE and C5 based on molecular dynamics simulation using ProLIF (occupancy >30%)	4
**Table S6.** Interaction analysis between MAGL and C3 based on molecular dynamics simulation using ProLIF (occupancy >30%)	5
**Table S7.** Interaction analysis between MAGL and C5 based on molecular dynamics simulation using ProLIF (occupancy >30%)	6
**Table S8.** Molar quantities of reagents used for the preparation of chrysin carbamate derivatives (**C1**–**C6**)	7
**Table S9.** Molar quantities of reagents used for the preparation of kaempferol carbamate derivatives (**K1**–**K6**)	7
**Fig. S1.** Spectral data of compound **C1**	9
**Fig. S2.** Spectral data of compound **C2**	11
**Fig. S3.** Spectral data of compound **C3**	14
**Fig. S4.** Spectral data of compound **C4**	17
**Fig. S5.** Spectral data of compound **C5**	19
**Fig. S6.** Spectral data of compound **C6**	21
**Fig. S7.** Spectral data of compound **K1**	23
**Fig. S8.** Spectral data of compound **K2**	25
**Fig. S9.** Spectral data of compound **K3**	28
**Fig. S10.** Spectral data of compound **K4**	31
**Fig. S11.** Spectral data of compound **K5**	33
**Fig. S12.** Spectral data of compound **K6**	35

Table S1. Docking results between carbamate derivatives and enzymes

Comp.	ΔG (kcal/mol)		Hydrogen bonds		Hydrophobic interactions	
	AChE	MAGL	AChE	MAGL	AChE	MAGL
C1	-9.2	-10.5		Met123	Tyr124, Trp286, Tyr337	Ala51, Ile179, Leu184, Tyr194, Leu241, His269, Val270
C2	-10.4	-9.4	Ser125, Ser203, His447		Tyr124, Val294, Tyr337, Tyr341	Ala51, Ile179, Leu205, Leu241
C3	-9.4	-10.3	Gly122, Ser203, His447	Ala51, Met123	Trp86, Tyr124, Tyr337, Tyr341	Ile179, Leu184, Tyr194, Leu205, Leu241, Val270, Sys273
C4	-10.4	-9.4	Gly122, Ser125, Ser203, His447		Trp86, Pro88, Tyr124, Val294, Tyr337, Tyr341	Ala51, Ala151, Ala156, Phe159, Ile179, Leu205, Leu214, Leu241
C5	-9.4	-10.4	Ser203, His447	Met123	Trp86, Tyr124, Val294, Tyr337, Tyr341	Ala51, Ile179, Tyr194, Leu205, Leu241, His269, Val270
C6	-10.3	-9.3	Gly121, Gly122, Ser125		Trp86, Tyr337, Tyr341	Ala51, Phe159, Ile179, Leu205, Leu241
K1	-9.2	-9.7	Gly121, Ser125, Ser203, Phe295, Arg296, His447	Ser176, Gly177	Trp86, Tyr124, Trp286, Tyr337, Phe338	Leu205, Leu213, Leu241
K2	-9	-8.2	Gly121, Ser125, Ser203, Phe295, Arg296, His447	Ser155, Ser176, Gly177	Trp86, Tyr124, Trp286, Tyr337	Leu213, Leu241
K3	-6.7	-8.3		Ser122	Trp286, Tyr337	Ala51, His121, Leu148, Ala151, Phe159, Ile179, Leu205, Leu213, Val217, Leu241
K4	-7.9	-7.7	Gly121, Ser125, Ser203, Trp286, His447	Ser122, Arg240	Leu76, Trp86, Trp286	His121, Leu148, Ala151, Ile179, Leu205, Leu213, Val217, Leu241
K5	-8.3	-10.2	Gly121, Ser125, Ser203, His447	Gly177	Trp86, Trp286	Ala51, Leu148, Ala151, Ile179, Tyr194, Leu213, Leu241, His269, Val270
K6	-8	-7.7	Gly121, Ser125, Ser203, Trp286, His447	Ser122	Trp86, Trp286, Phe338	His121, Leu148, Ala151, Ile179, Leu205, Leu213, Leu214, Val217, Leu241, Val270
Chrysin	-9.9	-9.8		Ala51	Trp86	Ile179, Tyr194, Leu241, His269, Val270
Kaempferol	-9.9	-9.8	Asn87		Trp86	Ala51, Ile179, Leu184, Tyr194, Leu241, His269, Val270
Rivastigmine	-7.2	ND	Ser125	-	Trp86, Tyr337	-
JZL-184	ND	-9.8	-	Ala51, Met123, Arg240	-	Leu213, Leu241

Table S2. Stability parameters of enzymes in the apoprotein state and ligand-bound complexes from MD simulations

Complex	RMSD (nm)		RMSF (nm)		Rg (nm)		SASA (nm^2)	
	AChE	MAGL	AChE	MAGL	AChE	MAGL	AChE	MAGL
Apo	0.175 ± 0.018	0.159 ± 0.015	0.08 ± 0.043	0.085 ± 0.045	2.324 ± 0.007	1.853 ± 0.009	219.393 ± 3.34	135.049 ± 1.97
C3	0.177 ± 0.019	0.137 ± 0.014	0.087 ± 0.054	0.076 ± 0.042	2.326 ± 0.008	1.844 ± 0.007	221.67 ± 4.324	134.208 ± 2.208
C5	0.191 ± 0.026	0.154 ± 0.015	0.09 ± 0.065	0.074 ± 0.035	2.329 ± 0.009	1.843 ± 0.007	221.741 ± 3.679	133.815 ± 1.919

Table S3. Stability parameters of ligands C3 and C5 in enzyme-ligand complexes

Complex	RMSD (nm)		RMSF (nm)	
	C3	C5	C3	C5
AChE	0.134 ± 0.018	0.141 ± 0.018	0.051 ± 0.048	0.05 ± 0.048
MAGL	0.111 ± 0.026	0.118 ± 0.021	0.041 ± 0.037	0.04 ± 0.039

Table S4. Interaction analysis between AChE and C3 based on molecular dynamics simulation using ProLIF (occupancy >30%)

**Ligand**	**Protein**	**Interaction**	**Occupancy (%)**
LIG543.B	TRP86.A	Hydrophobic	99.2008
LIG543.B	PHE297.A	Hydrophobic	97.3027
LIG543.B	PHE338.A	Hydrophobic	90.90909
LIG543.B	TYR337.A	Hydrophobic	90.30969
LIG543.B	TYR124.A	Hydrophobic	88.01199
LIG543.B	TRP86.A	VdWContact	80.21978
LIG543.B	TYR337.A	VdWContact	67.13287
LIG543.B	TYR341.A	Hydrophobic	66.03397
LIG543.B	TYR337.A	HBAcceptor	56.74326
LIG543.B	LEU76.A	Hydrophobic	49.95005
LIG543.B	GLY448.A	VdWContact	48.65135
LIG543.B	PHE297.A	VdWContact	47.25275
LIG543.B	PHE338.A	VdWContact	47.15285
LIG543.B	TYR449.A	Hydrophobic	46.15385
LIG543.B	HIS447.A	Hydrophobic	43.85614
LIG543.B	PHE295.A	Hydrophobic	43.65634
LIG543.B	TYR124.A	VdWContact	42.15784
LIG543.B	TYR341.A	PiStacking	40.35964
LIG543.B	TRP286.A	Hydrophobic	36.86314
LIG543.B	TYR341.A	VdWContact	36.36364
LIG543.B	VAL132.A	Hydrophobic	34.96503
LIG543.B	GLY121.A	VdWContact	31.46853

Table S5. Interaction analysis between AChE and C5 based on molecular dynamics simulation using ProLIF (occupancy >30%)

Ligand	Protein	Interaction	Occupancy (%)
LIG543.B	TYR337.A	Hydrophobic	99.1009
LIG543.B	TRP86.A	Hydrophobic	97.2028
LIG543.B	PHE338.A	Hydrophobic	94.20579
LIG543.B	PHE297.A	Hydrophobic	92.90709
LIG543.B	HIS447.A	Hydrophobic	92.70729
LIG543.B	PHE295.A	Hydrophobic	72.12787
LIG543.B	TRP86.A	VdWContact	71.72827
LIG543.B	TYR124.A	Hydrophobic	67.23277
LIG543.B	TYR337.A	VdWContact	62.93706
LIG543.B	HIS447.A	PiStacking	58.94106
LIG543.B	HIS447.A	VdWContact	53.24675
LIG543.B	HIS447.A	PiCation	44.15584
LIG543.B	LEU437.A	Hydrophobic	41.05894
LIG543.B	TYR124.A	VdWContact	37.76224
LIG543.B	PHE297.A	VdWContact	30.16983
LIG543.B	PHE295.A	VdWContact	29.17083

Table S6. Interaction analysis between MAGL and C3 based on molecular dynamics simulation using ProLIF (occupancy >30%)

Ligand	Protein	Interaction	Occupancy (%)
LIG296.B	VAL270.A	Hydrophobic	100
LIG296.B	LEU184.A	Hydrophobic	99.9001
LIG296.B	MET88.A	Hydrophobic	92.60739
LIG296.B	LEU205.A	Hydrophobic	92.00799
LIG296.B	LYS273.A	Hydrophobic	88.81119
LIG296.B	ILE179.A	Hydrophobic	88.01199
LIG296.B	HIS269.A	Hydrophobic	82.91708
LIG296.B	ALA51.A	Hydrophobic	80.11988
LIG296.B	HIS269.A	VdWContact	78.12188
LIG296.B	LEU241.A	Hydrophobic	67.33267
LIG296.B	VAL270.A	VdWContact	66.83317
LIG296.B	LEU184.A	VdWContact	66.03397
LIG296.B	CYS201.A	Hydrophobic	56.84316
LIG296.B	TYR194.A	VdWContact	55.84416
LIG296.B	TYR194.A	Hydrophobic	50.94905
LIG296.B	SER181.A	VdWContact	50.04995
LIG296.B	CYS201.A	VdWContact	45.55445
LIG296.B	ALA203.A	VdWContact	43.95604
LIG296.B	LEU205.A	VdWContact	40.45954
LIG296.B	ILE179.A	VdWContact	38.46154
LIG296.B	ALA51.A	VdWContact	37.36264
LIG296.B	MET88.A	VdWContact	36.76324
LIG296.B	LEU241.A	VdWContact	33.76623

Table S7. Interaction analysis between MAGL and C5 based on molecular dynamics simulation using ProLIF (occupancy >30%)

**Ligand**	**Protein**	**Interaction**	**Occupancy (%)**
LIG296.B	TYR194.A	Hydrophobic	99.9001
LIG296.B	LEU184.A	Hydrophobic	99.6004
LIG296.B	GLU53.A	Hydrophobic	95.1049
LIG296.B	VAL191.A	Hydrophobic	92.80719
LIG296.B	GLU53.A	VdWContact	88.11189
LIG296.B	ILE200.A	Hydrophobic	81.21878
LIG296.B	GLU53.A	HBDonor	77.72228
LIG296.B	LEU184.A	VdWContact	69.53047
LIG296.B	TYR194.A	PiStacking	68.73127
LIG296.B	MET88.A	Hydrophobic	61.43856
LIG296.B	LEU205.A	Hydrophobic	58.84116
LIG296.B	VAL270.A	Hydrophobic	57.84216
LIG296.B	TYR194.A	VdWContact	55.74426
LIG296.B	ARG57.A	VdWContact	54.94505
LIG296.B	ILE179.A	Hydrophobic	48.45155
LIG296.B	ALA203.A	VdWContact	43.55644
LIG296.B	CYS201.A	Hydrophobic	40.05994
LIG296.B	ALA51.A	VdWContact	39.46054
LIG296.B	ARG57.A	HBAcceptor	37.36264
LIG296.B	VAL191.A	VdWContact	36.36364
LIG296.B	ILE179.A	VdWContact	35.46454
LIG296.B	LEU205.A	VdWContact	32.96703
LIG296.B	ILE200.A	VdWContact	31.86813
LIG296.B	GLY52.A	VdWContact	30.86913

Table S8. Molar quantities of reagents used for the preparation of chrysin carbamate derivatives (**C1**–**C6**)

**Comp.**	**Chrysin (mmol)**	***N*,*N*-dimethylcarbamoyl clorid (mmol)**	***N*,*N*-dimethylcarbamoyl clorid (mmol)**	***N*-ethyl-*N*-methylcarbamoyl clorid (mmol)**	**K_2CO_3 (mmol)**
**C1**	5	5			10
**C2**	5	12			20
**C3**	5		5		10
**C4**	5		12		20
**C5**	5			5	10
**C6**	5			12	20

Table S9. Molar quantities of reagents used for the preparation of kaempferol carbamate derivatives (**K1**–**K6**)

**Comp.**	**Kaempferol (mmol)**	***N*,*N*-dimethylcarbamoyl clorid (mmol)**	***N*,*N*-dimethylcarbamoyl clorid (mmol)**	***N*-ethyl-*N*-methyl carbamoyl clorid (mmol)**	**K_2CO_3 (mmol)**
**K1**	5	15			30
**K2**	5	25			46
**K3**	5		15		30
**K4**	5		25		46
**K5**	5			15	30
**K6**	5			25	46

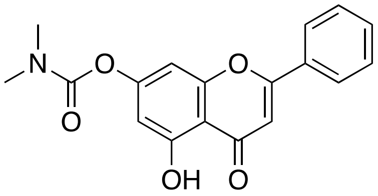

(A) UV spectrum of C1

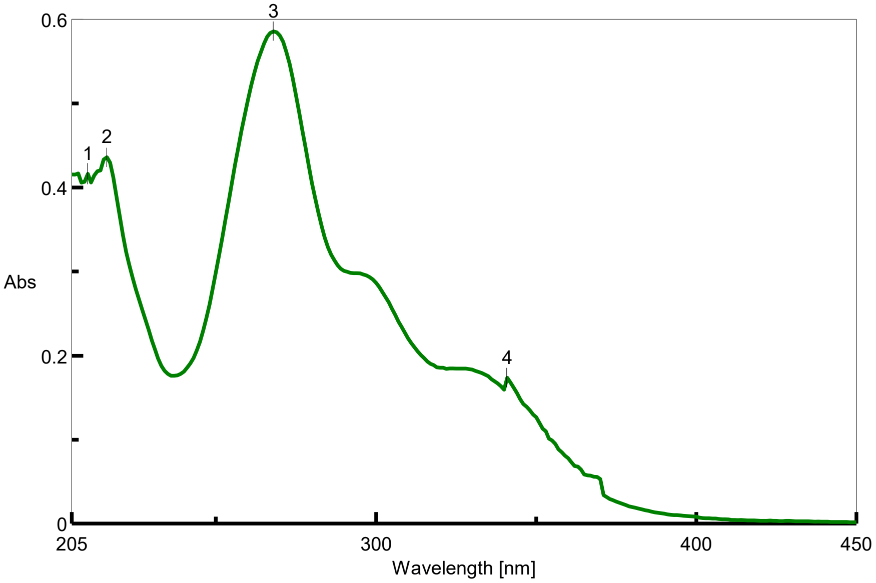

(B) HRMS spectrum of C1

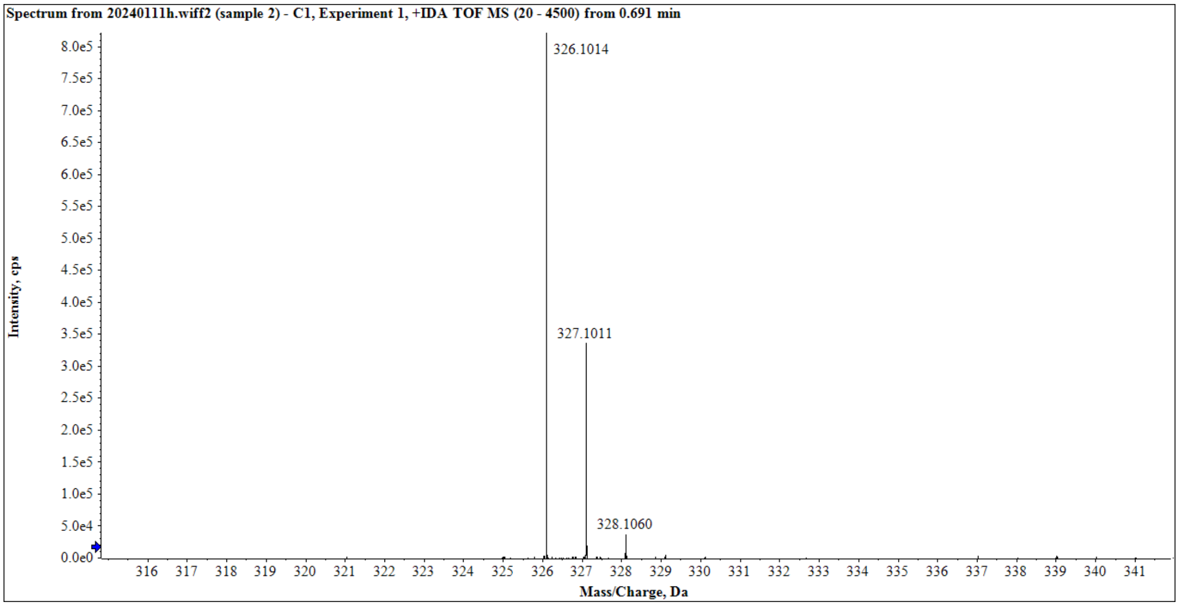

(C) ¹H-NMR spectrum of C1

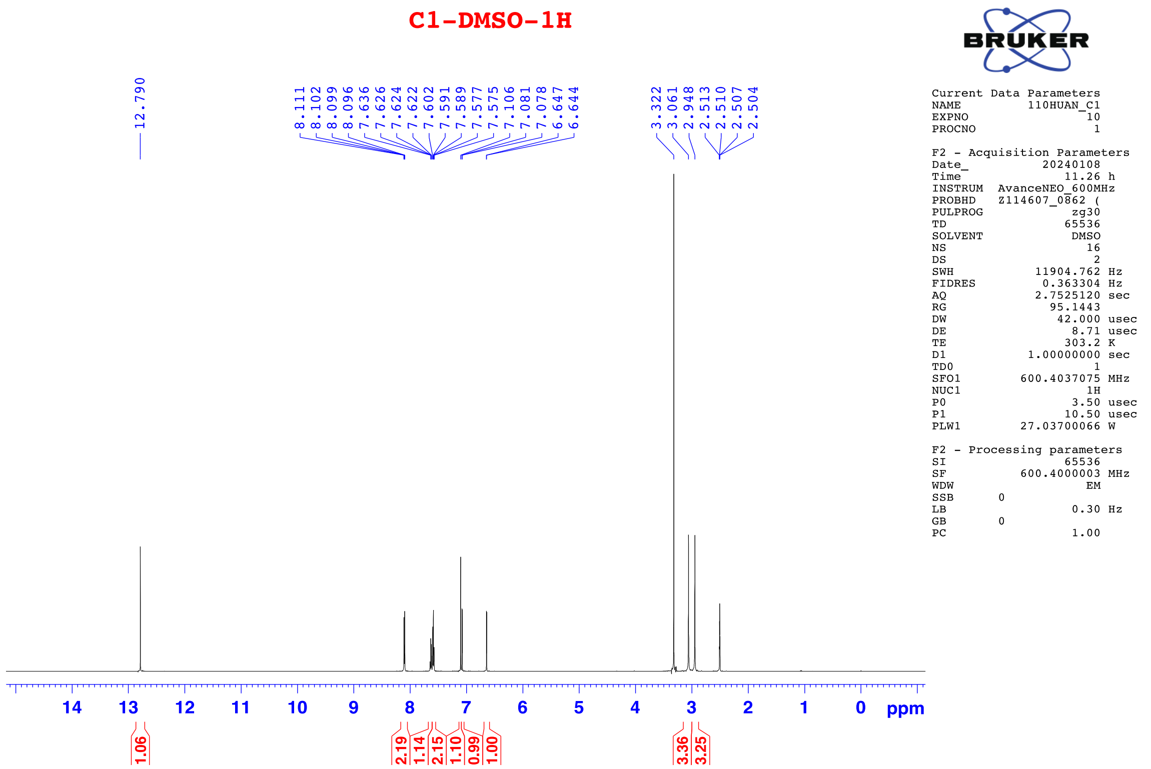

(D) ¹³C-NMR spectrum of C1

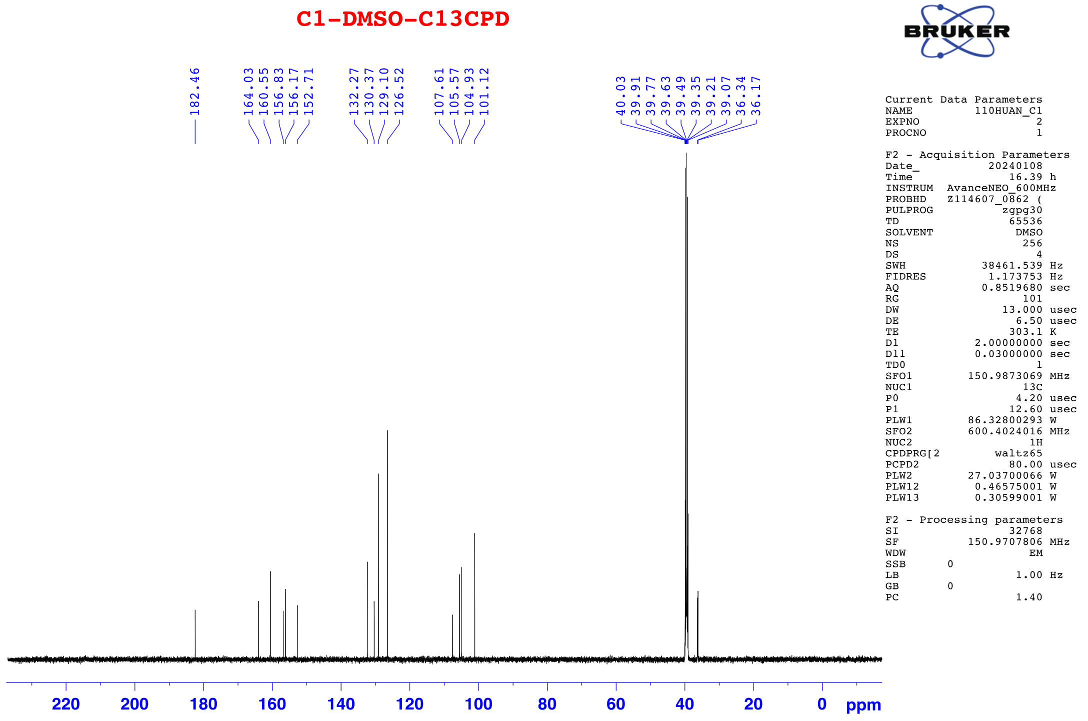

Fig. S1. Spectral data of compound **C1**

A. UV spectrum B. HRMS spectrum C. ¹H-NMR spectrum D. ¹³C-NMR spectrum

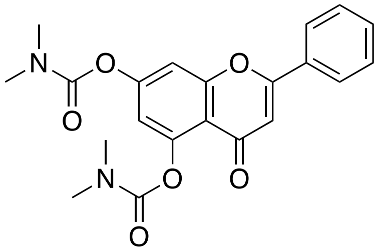

(A) UV spectrum of C2

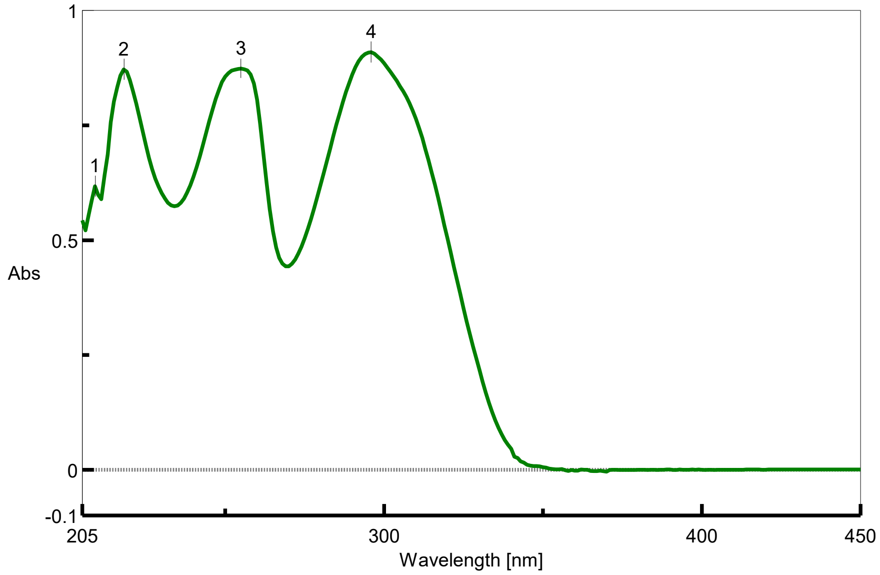

(B) HRMS spectrum of C2

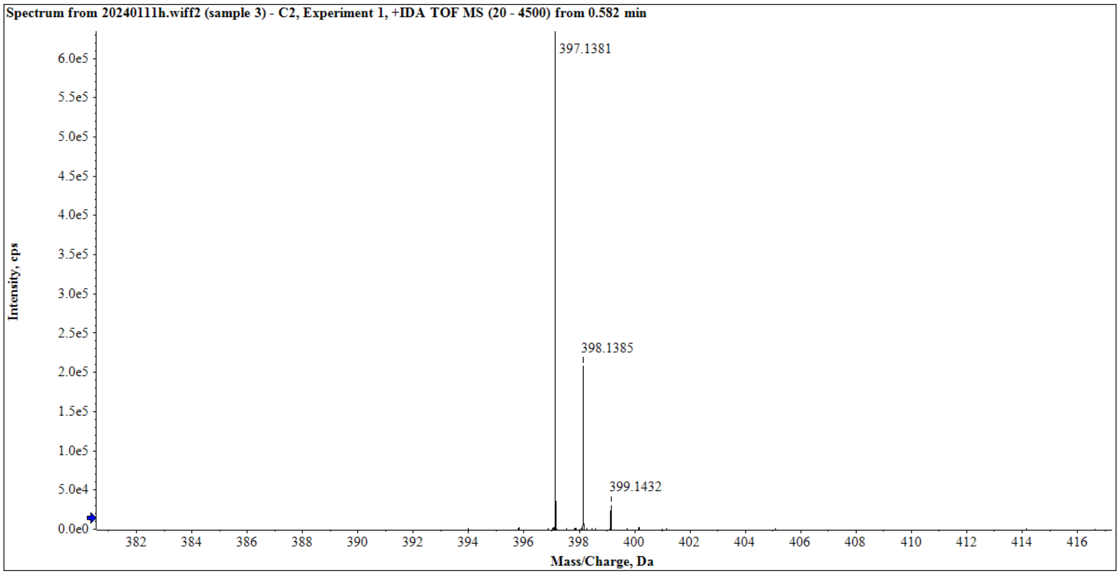

(C) ^{1}H-NMR spectrum of C2

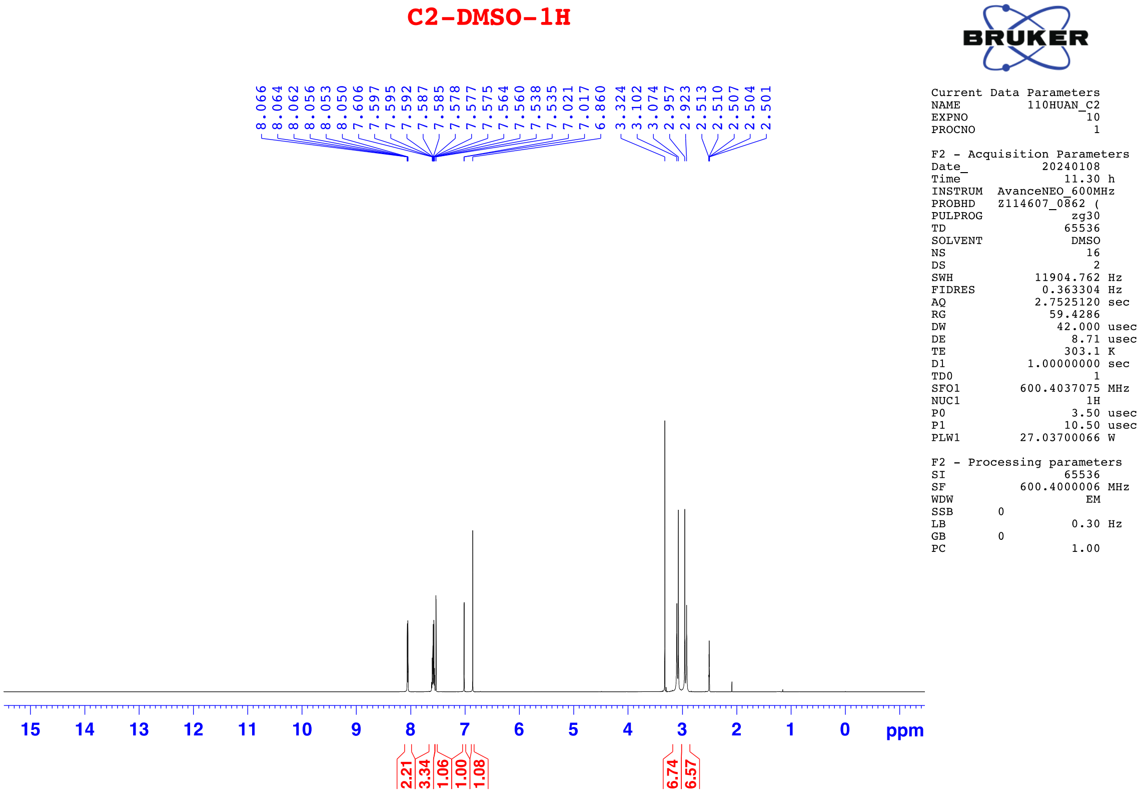

(D) ^{13}C-NMR spectrum of C2

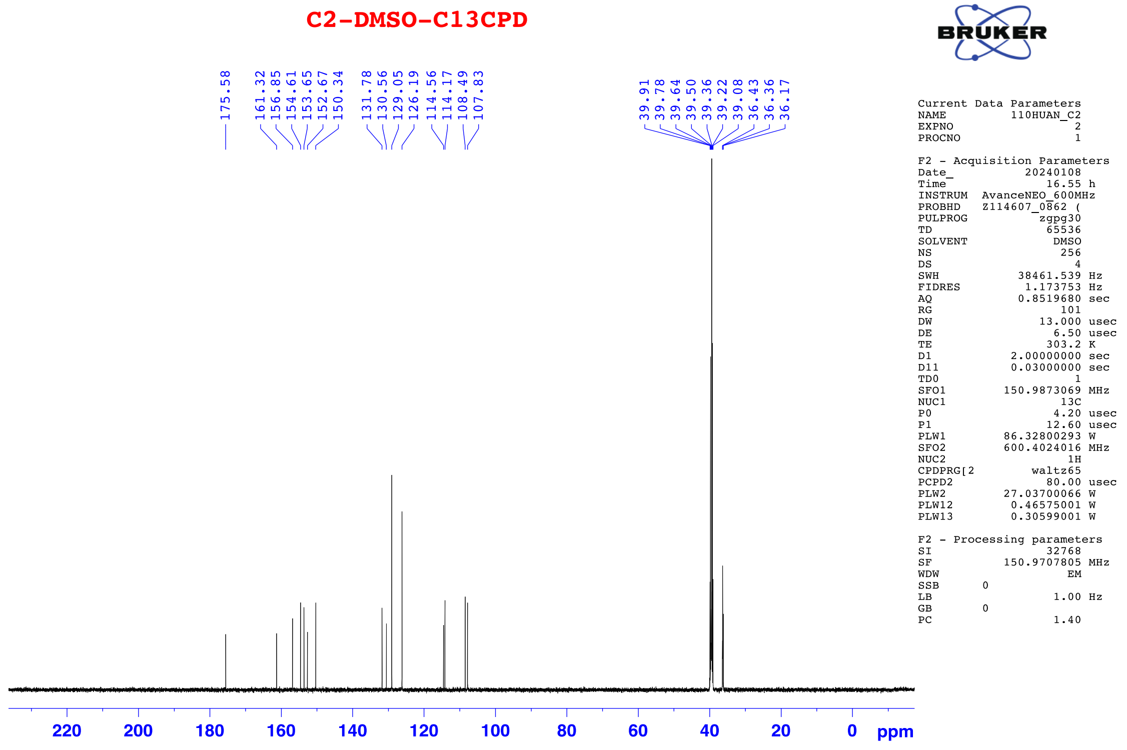

Fig. S2. Spectral data of compound **C2**

A. UV spectrum B. HRMS spectrum C. ^{1}H-NMR spectrum D. ^{13}C-NMR spectrum

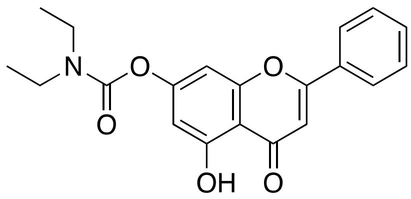

(A) UV spectrum of C3

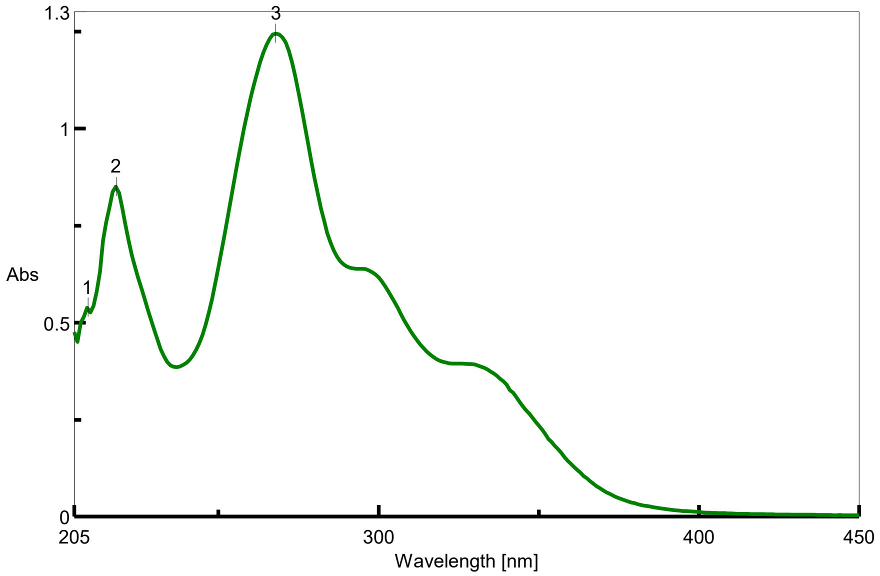

(B) HRMS spectrum of C3

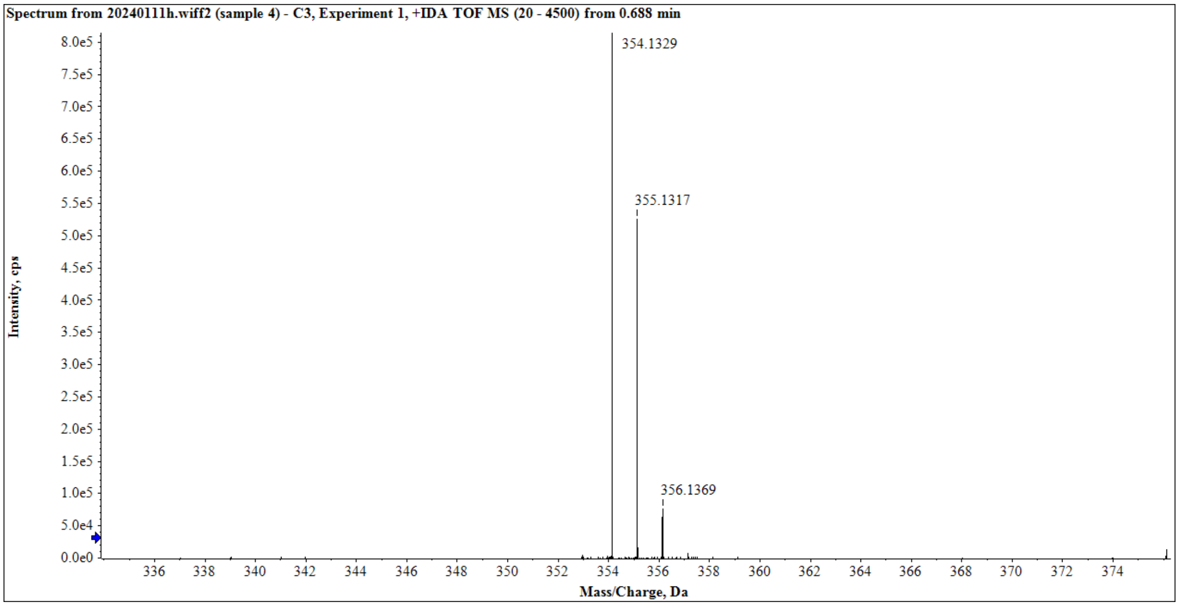

(C) ¹H-NMR spectrum of C3

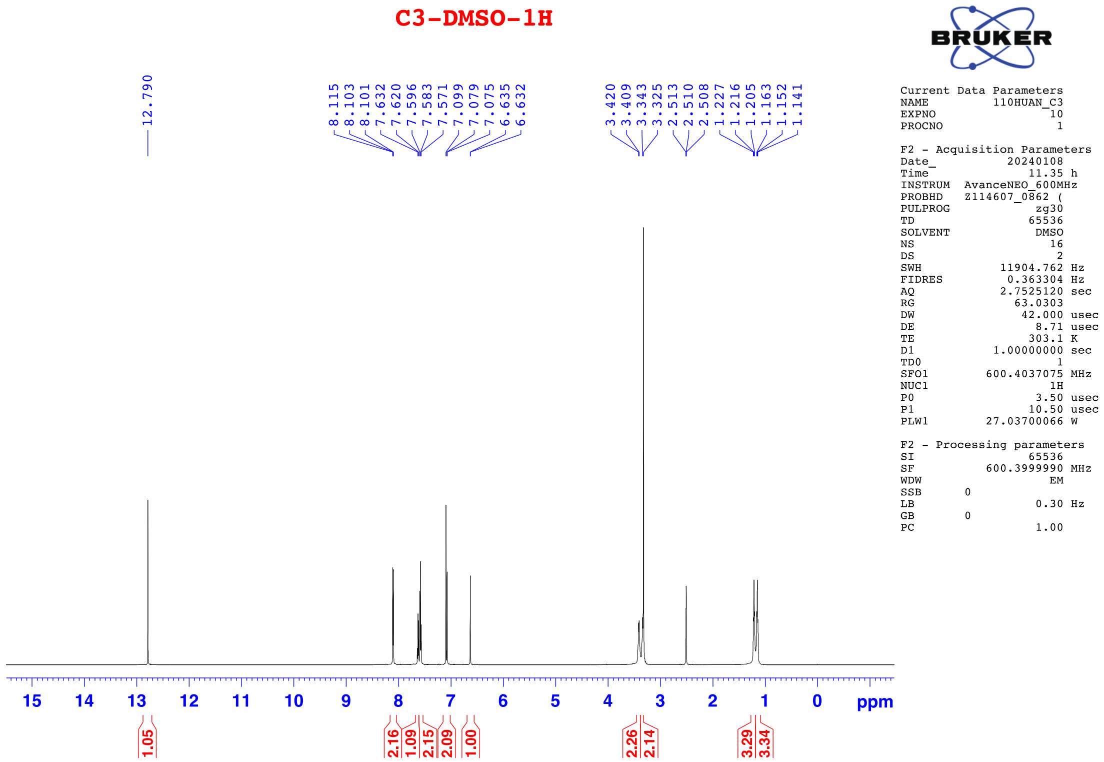

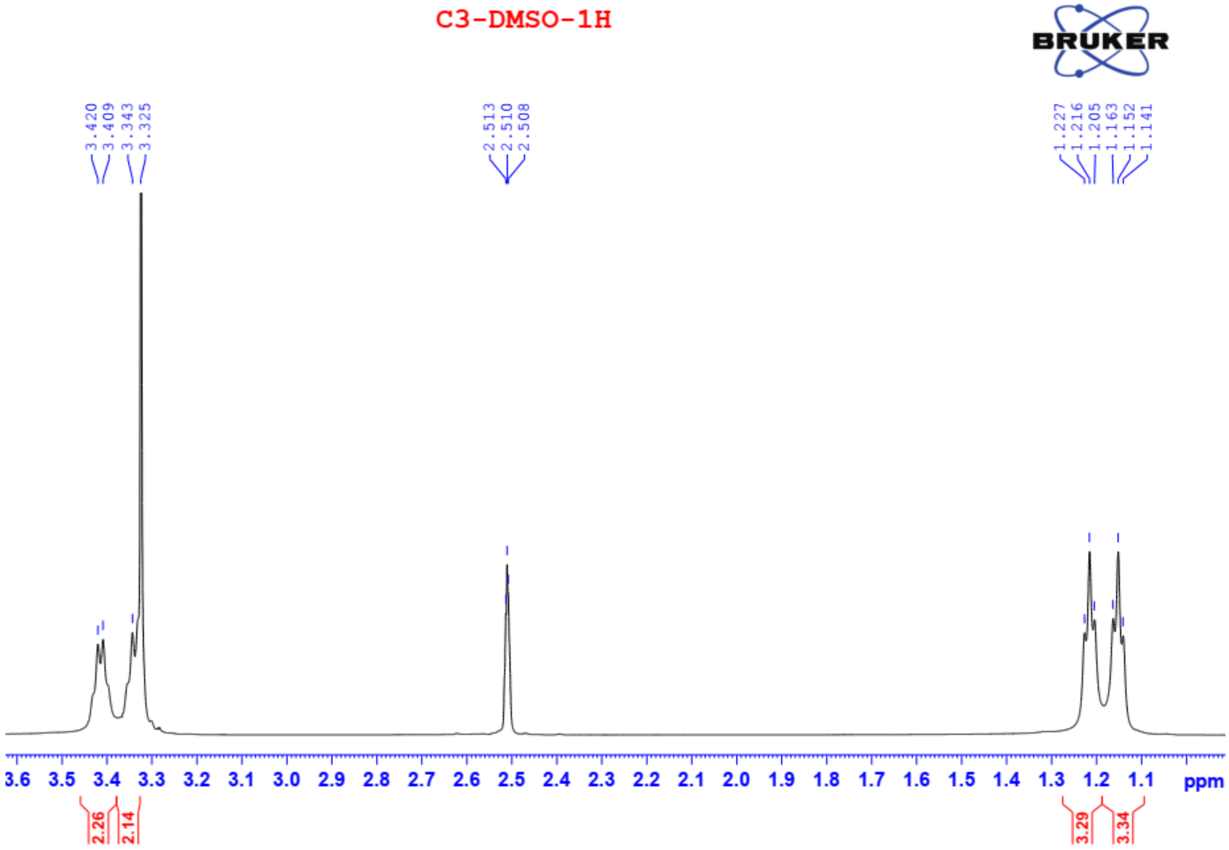

(D) ^{13}C-NMR spectrum of C3

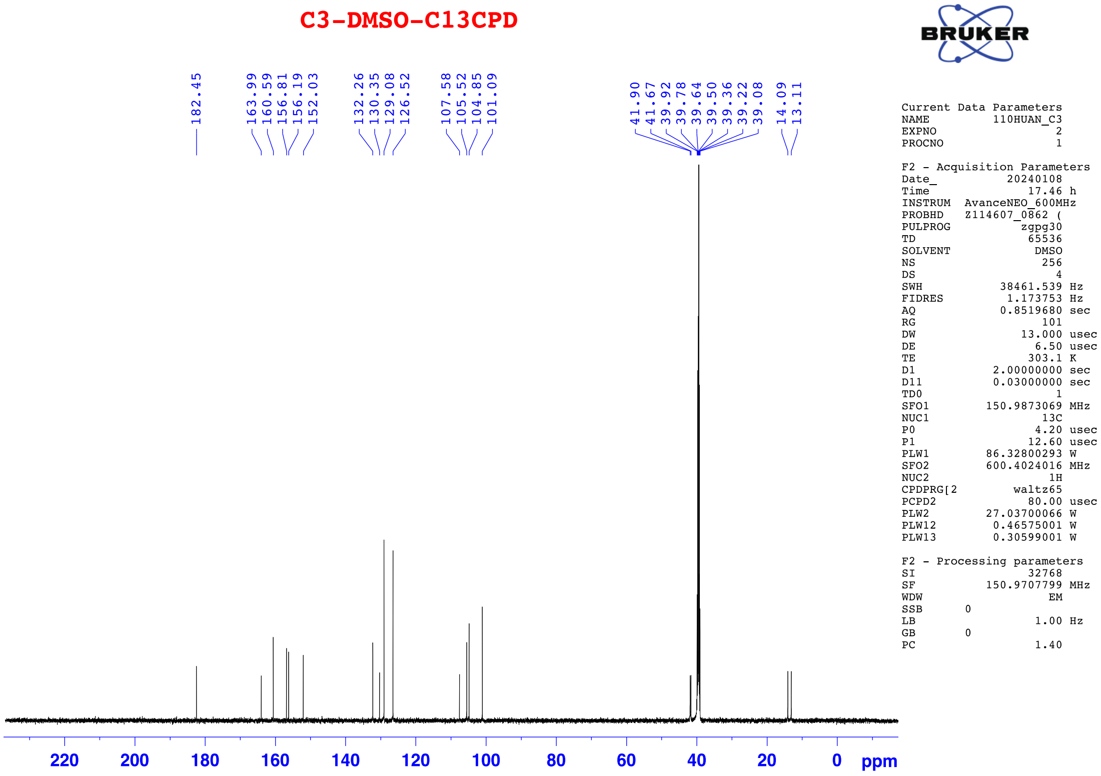

Fig. S3. Spectral data of compound **C3**

A. UV spectrum B. HRMS spectrum C. ^{1}H-NMR spectrum D. ^{13}C-NMR spectrum

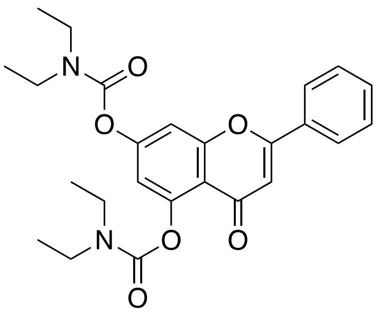

(A) UV spectrum of C4

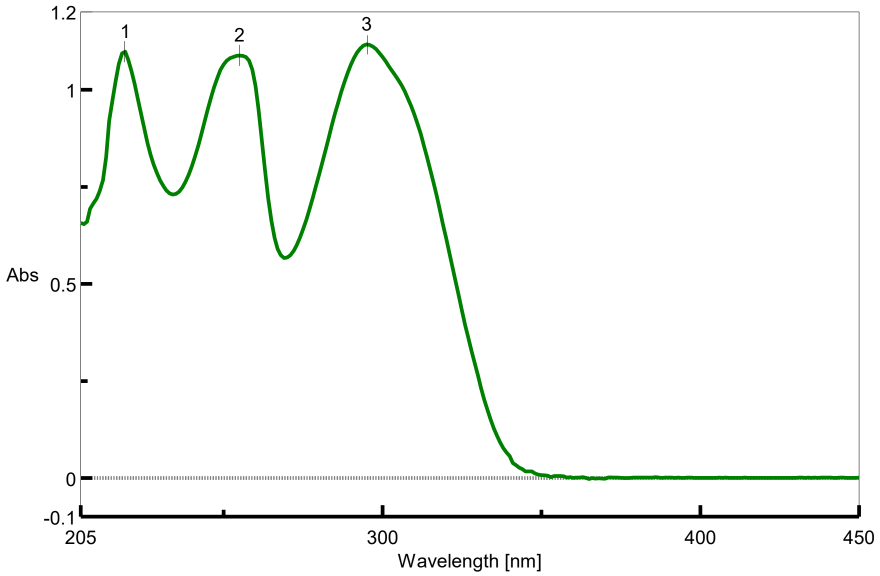

(B) HRMS spectrum of C4

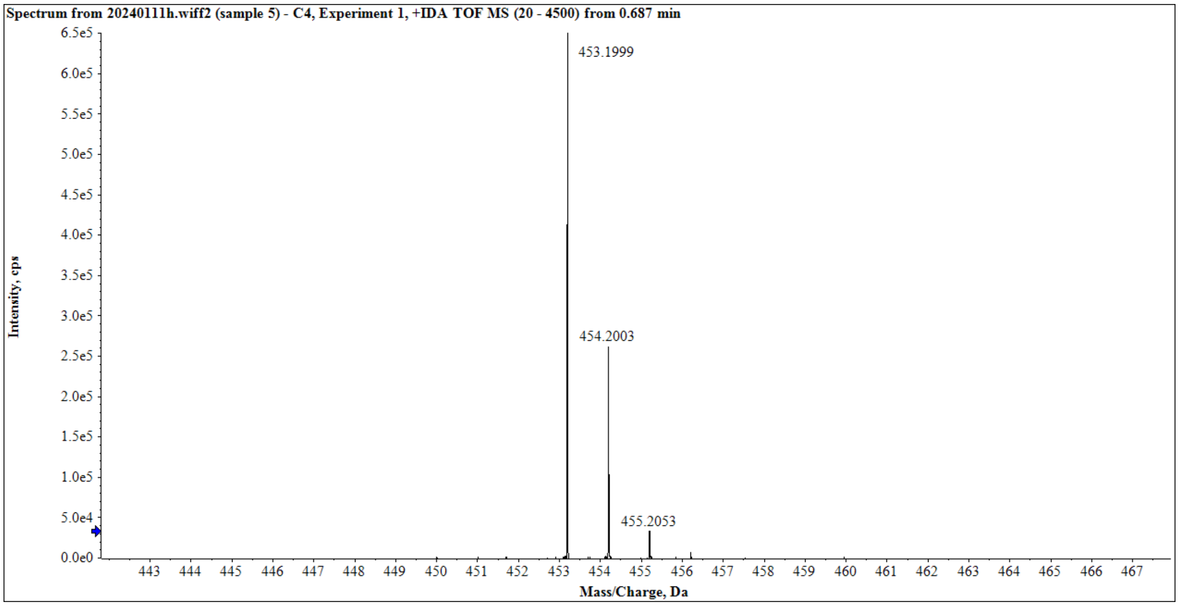

(C) ¹H-NMR spectrum of C4

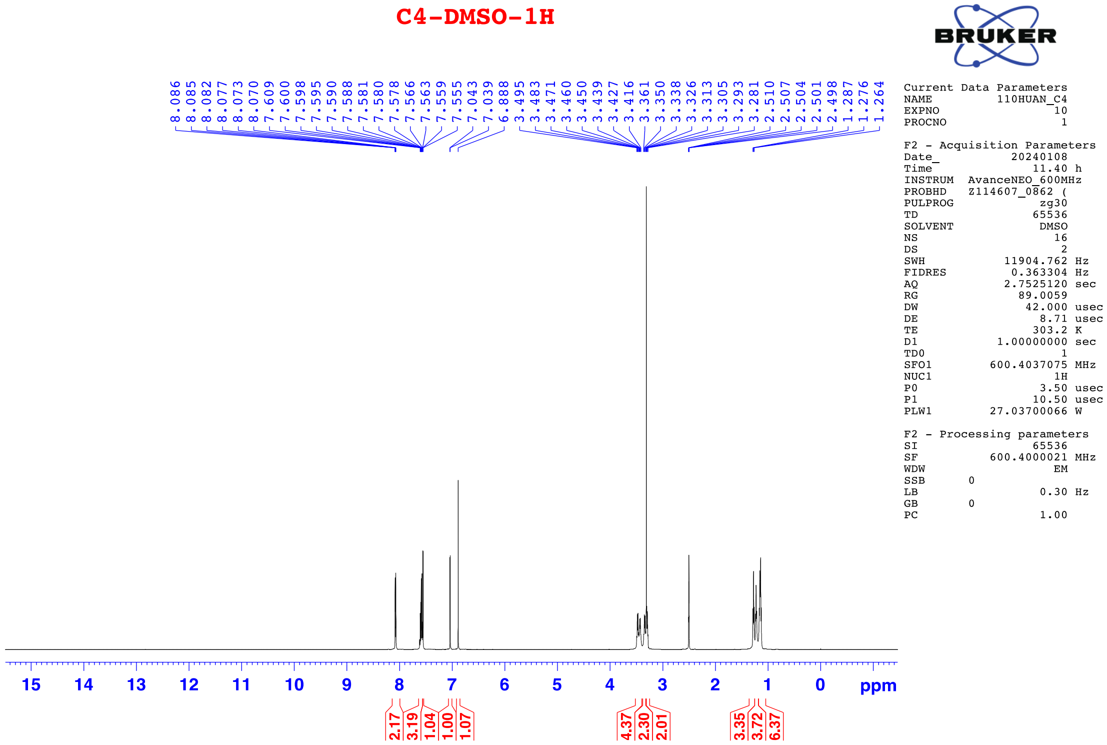

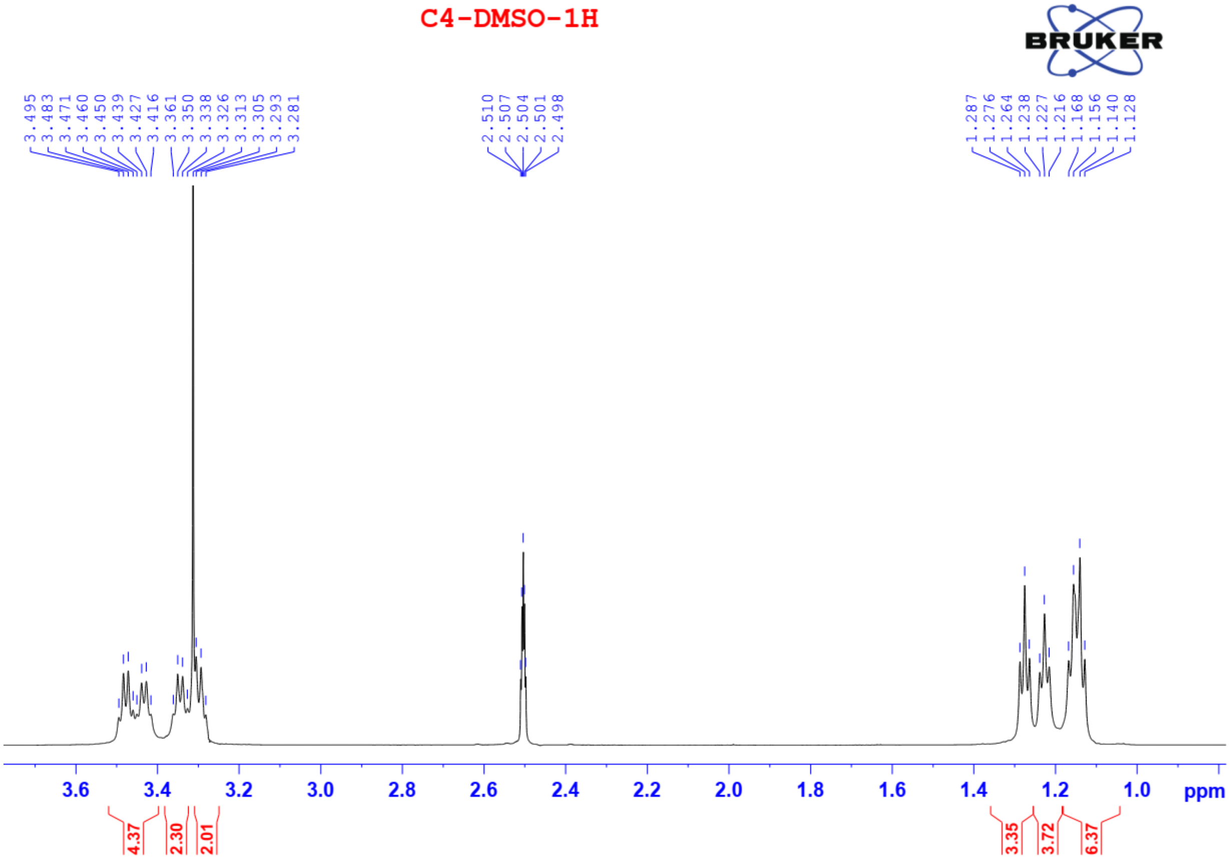

(D) ^{13}C-NMR spectrum of C4

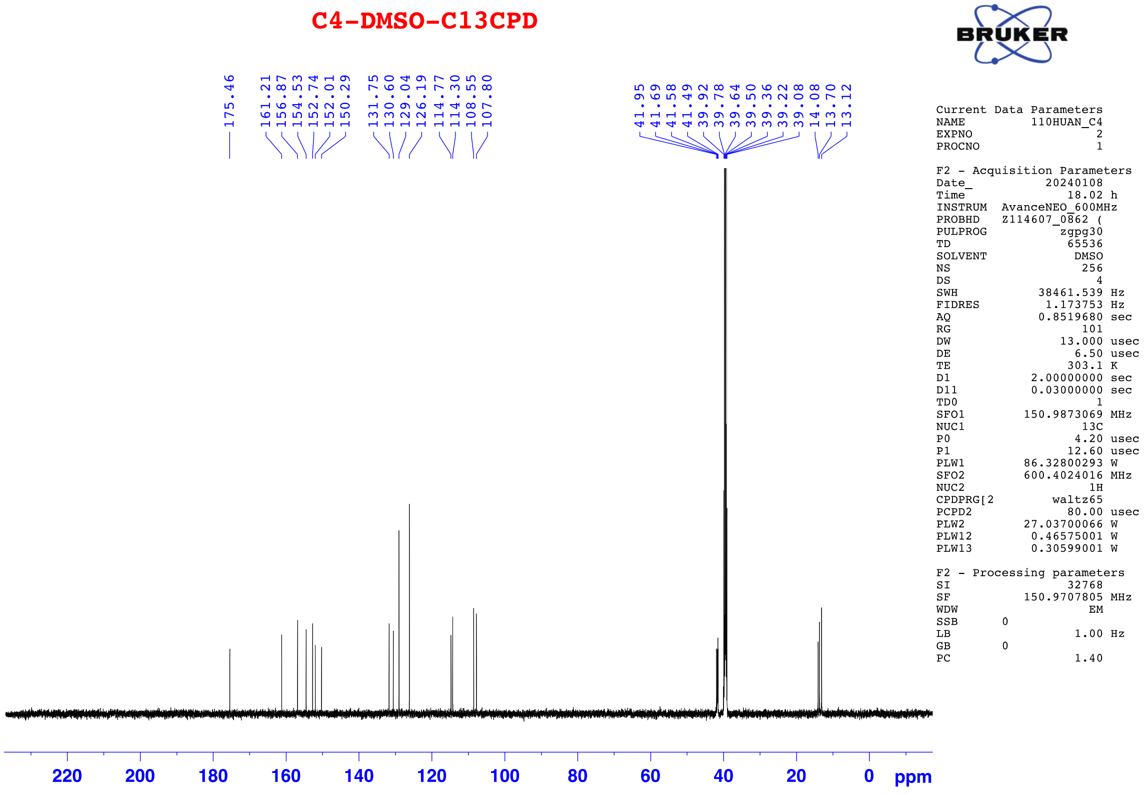

Fig. S4. Spectral data of compound **C4**

A. UV spectrum B. HRMS spectrum C. ^{1}H-NMR spectrum D. ^{13}C-NMR spectrum

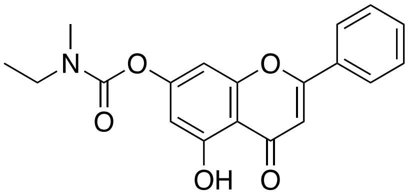

(A) UV spectrum of C5

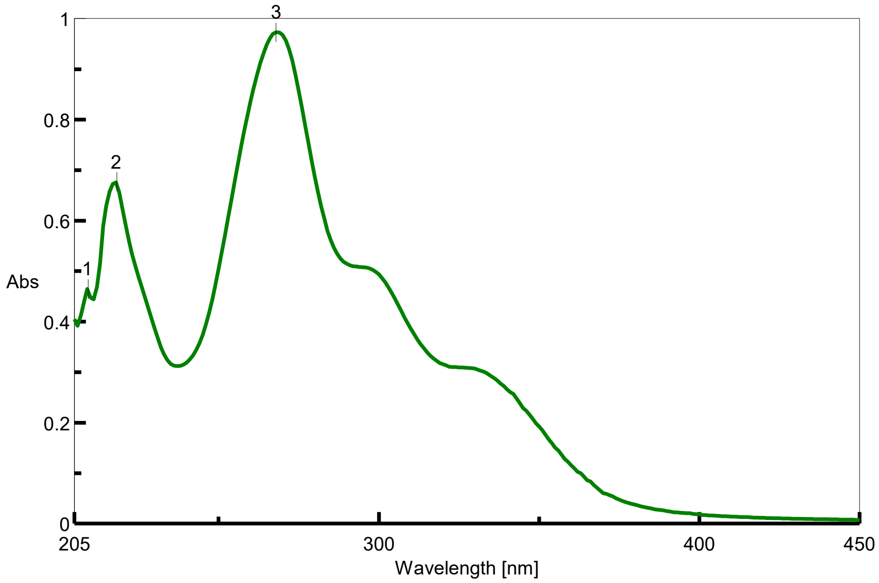

(B) HRMS spectrum of C5

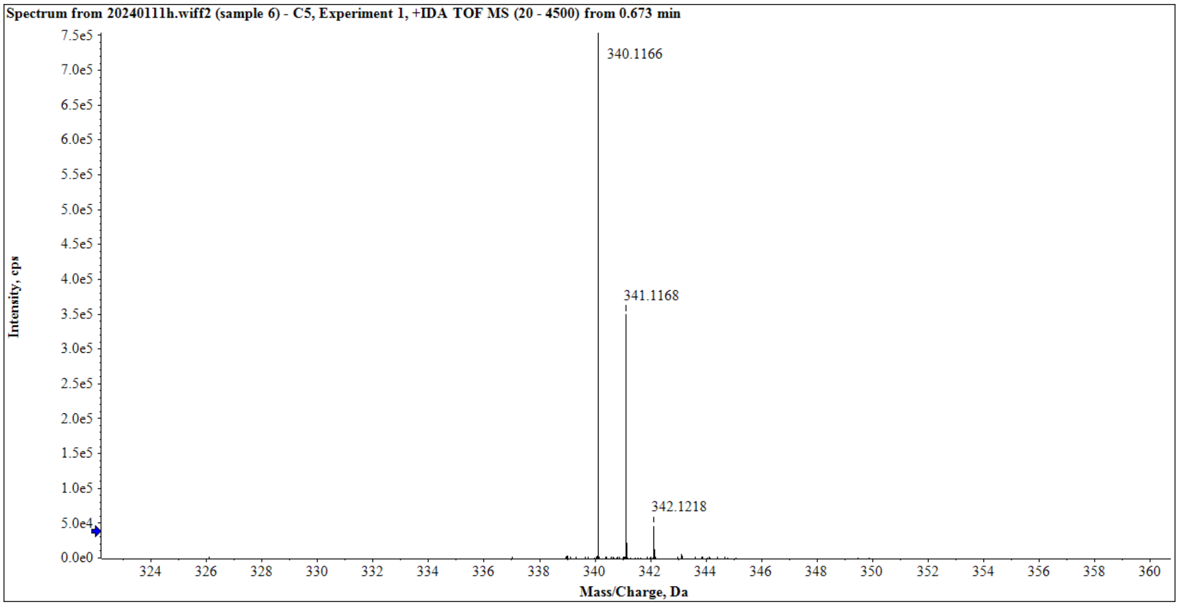

(C) ^{1}H-NMR spectrum of C5

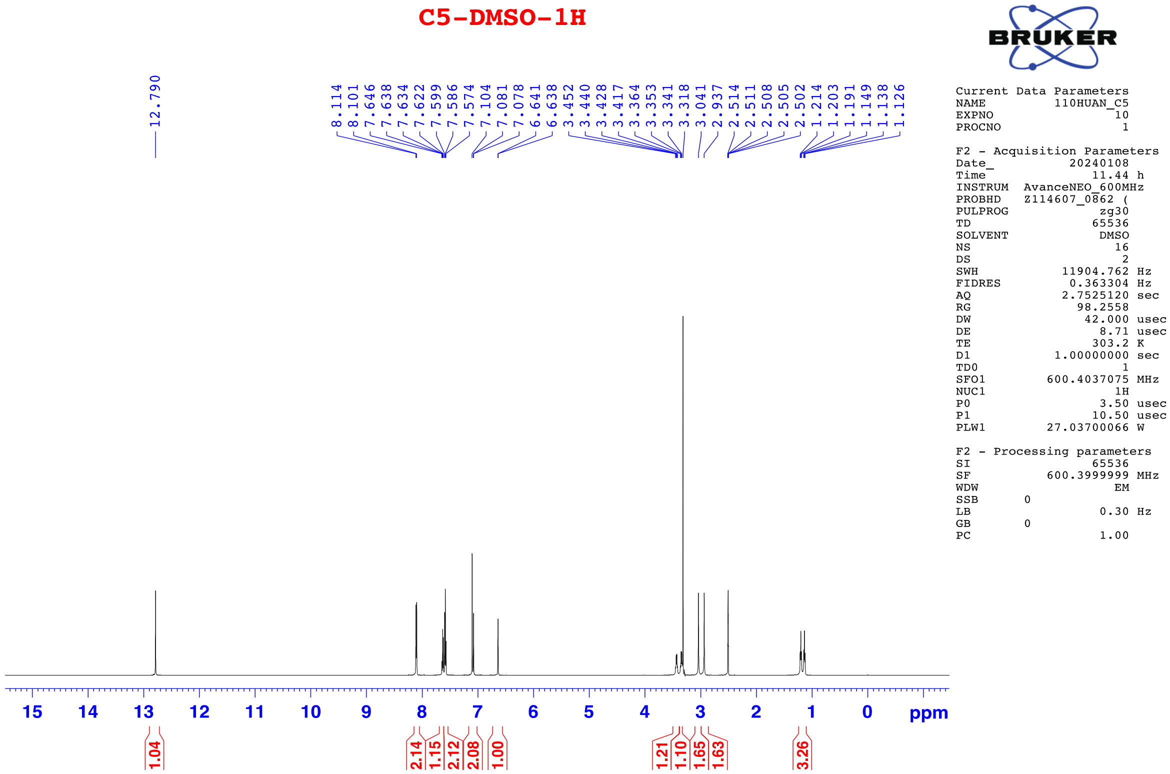

(D) ^{13}C-NMR spectrum of C5

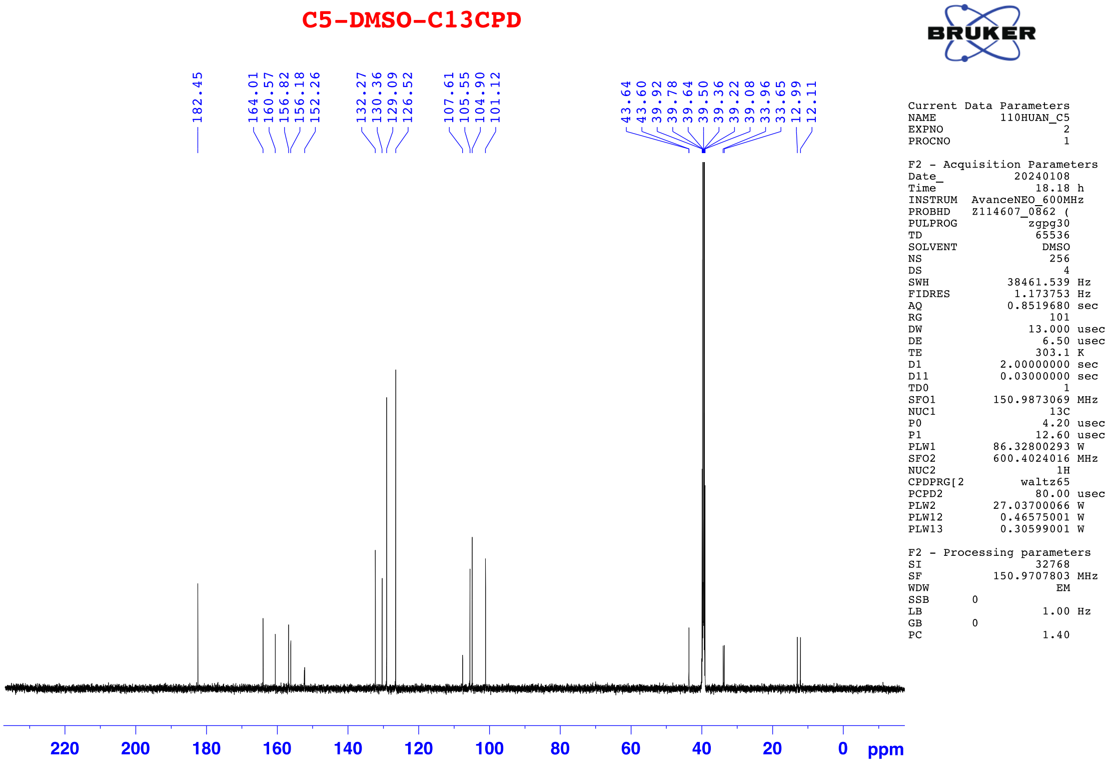

Fig. S5. Spectral data of compound **C5**

A. UV spectrum B. HRMS spectrum C. ^{1}H-NMR spectrum D. ^{13}C-NMR spectrum

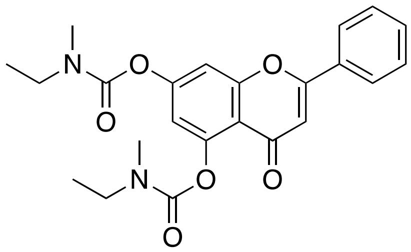

(A) UV spectrum of C6

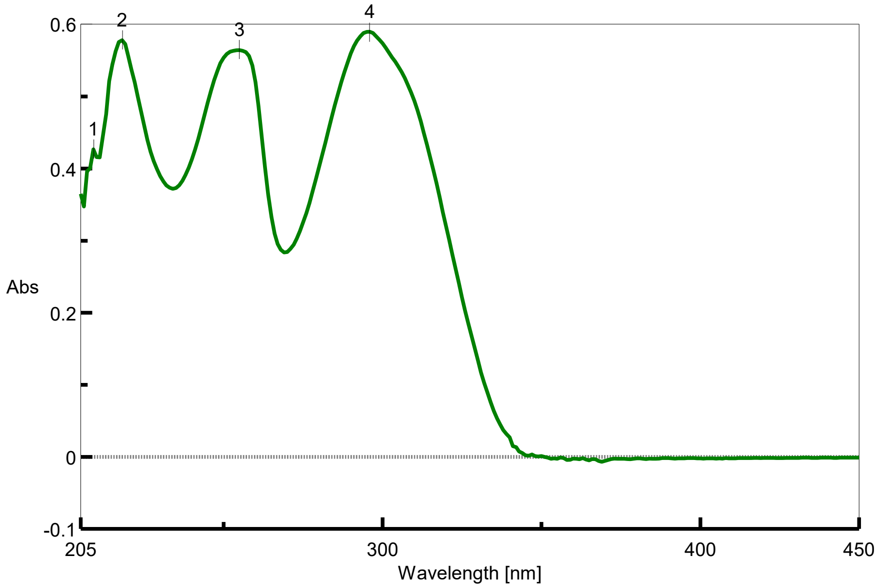

(B) HRMS spectrum of C6

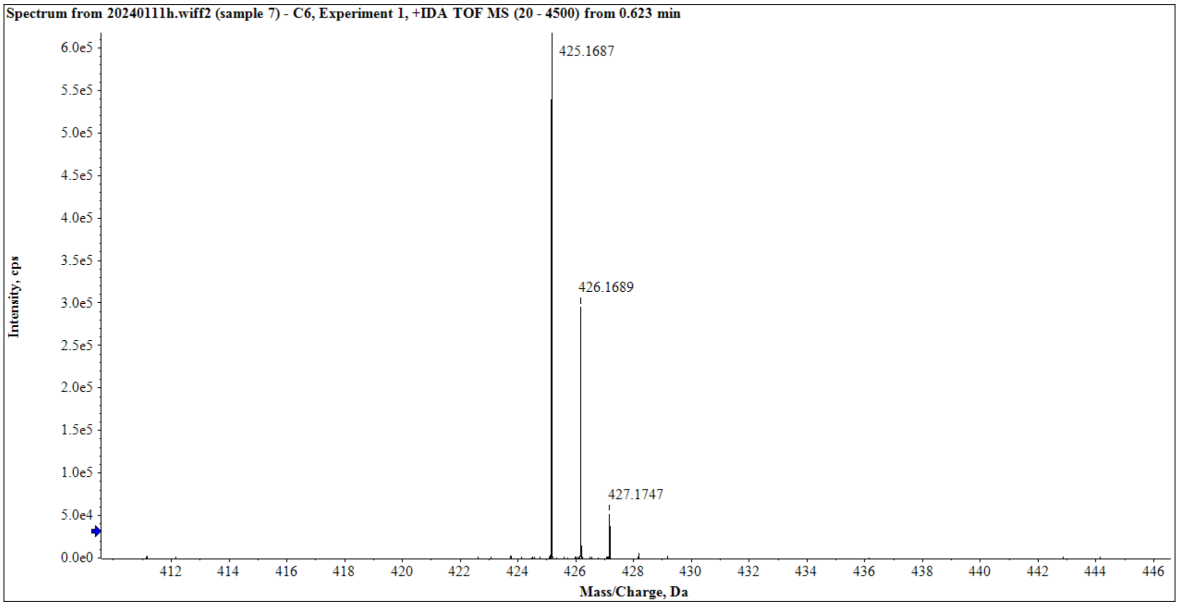

(C) ^{1}H-NMR spectrum of C6

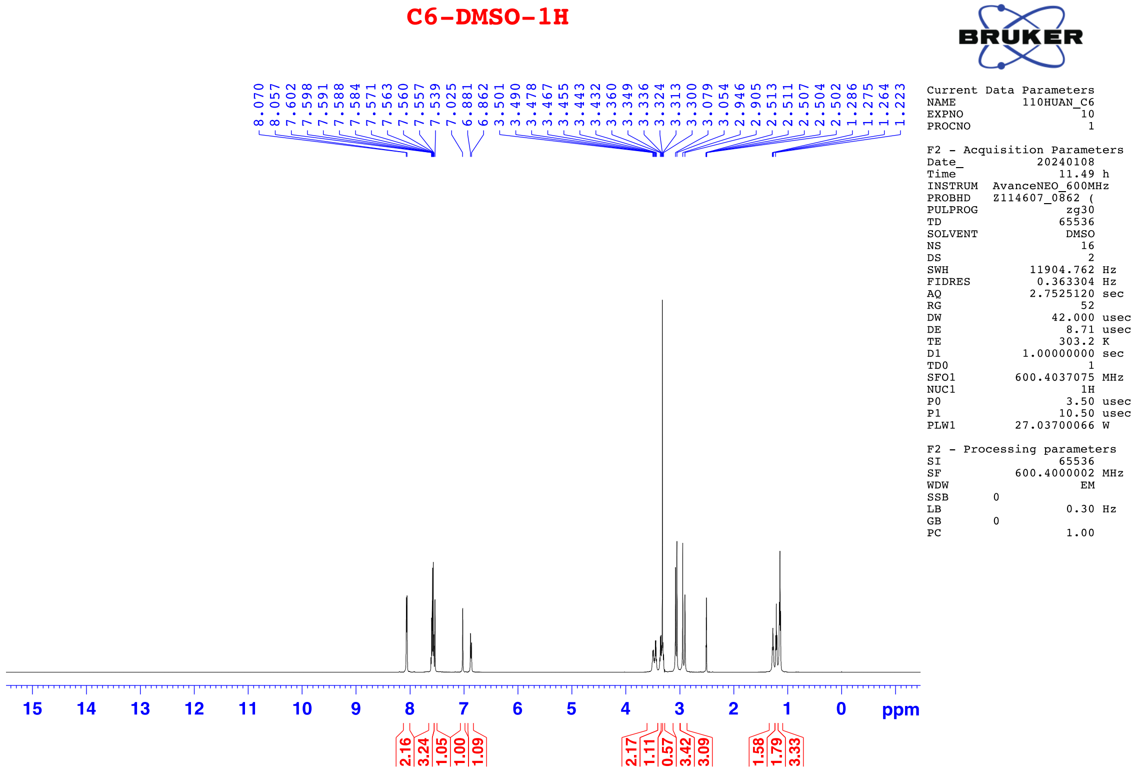

(D) ^{13}C-NMR spectrum of C6

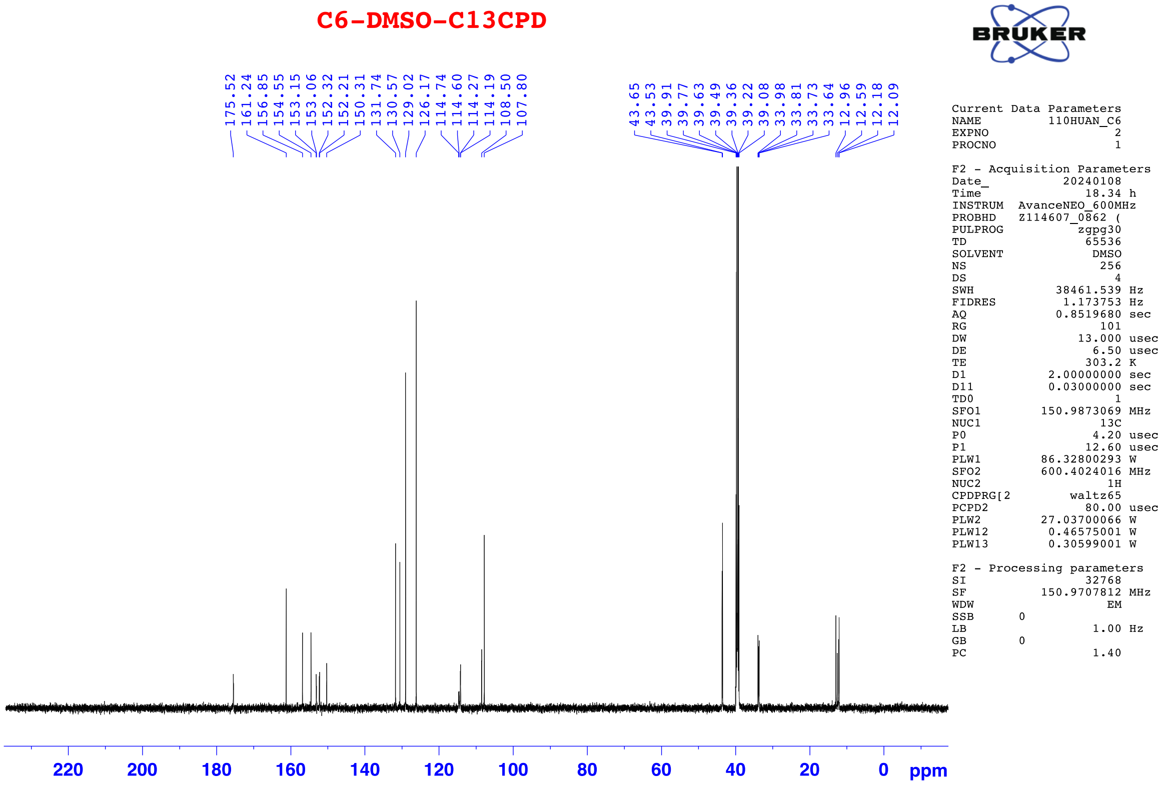

Fig. S6. Spectral data of compound **C6**

A. UV spectrum B. HRMS spectrum C. ^{1}H-NMR spectrum D. ^{13}C-NMR spectrum

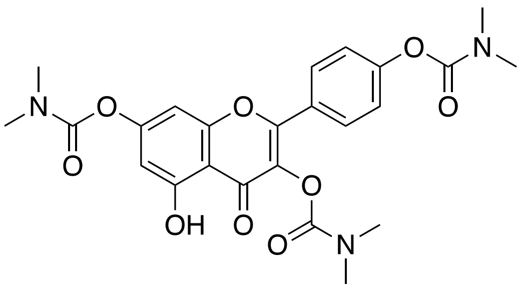

(A) UV spectrum of K1

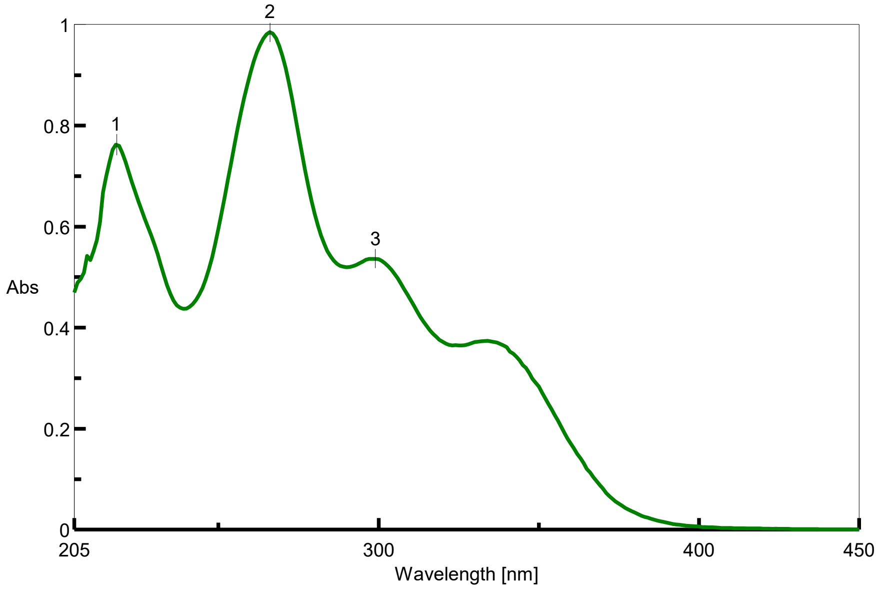

(B) HRMS spectrum of K1

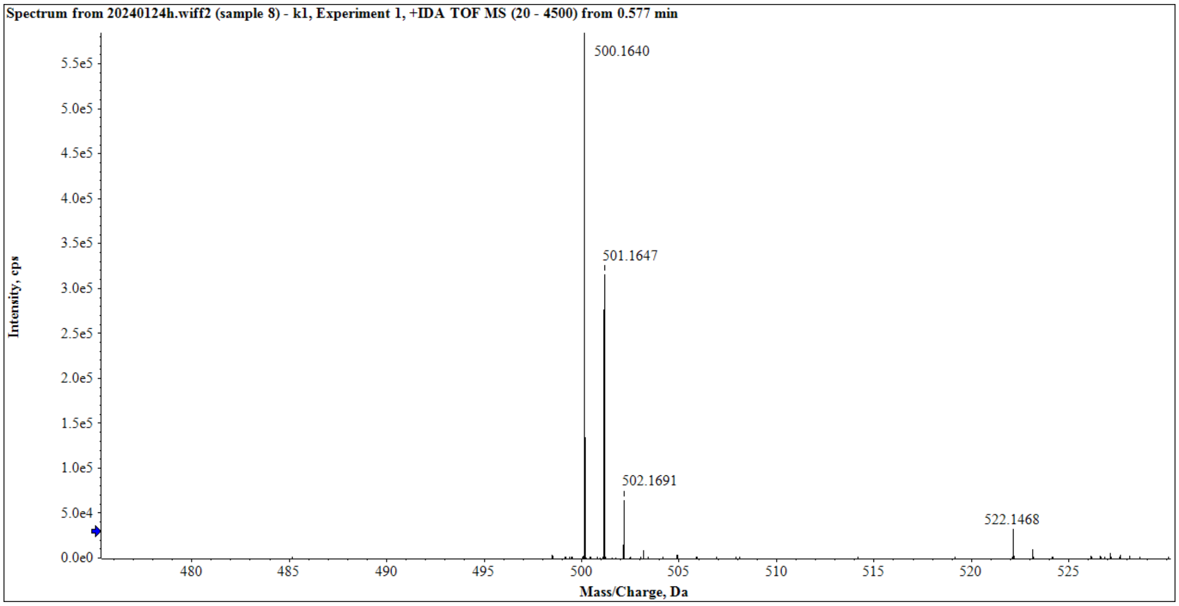

(C) ^{1}H-NMR spectrum of K1

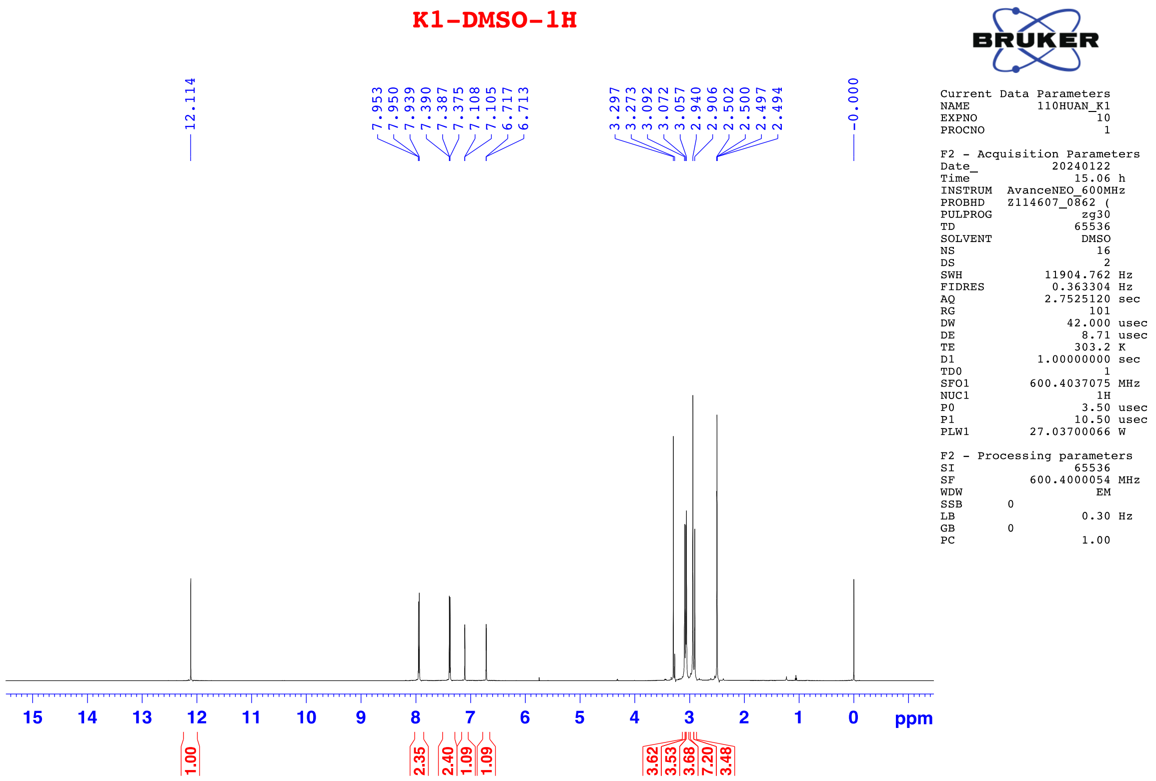

(D) ^{13}C-NMR spectrum of K1

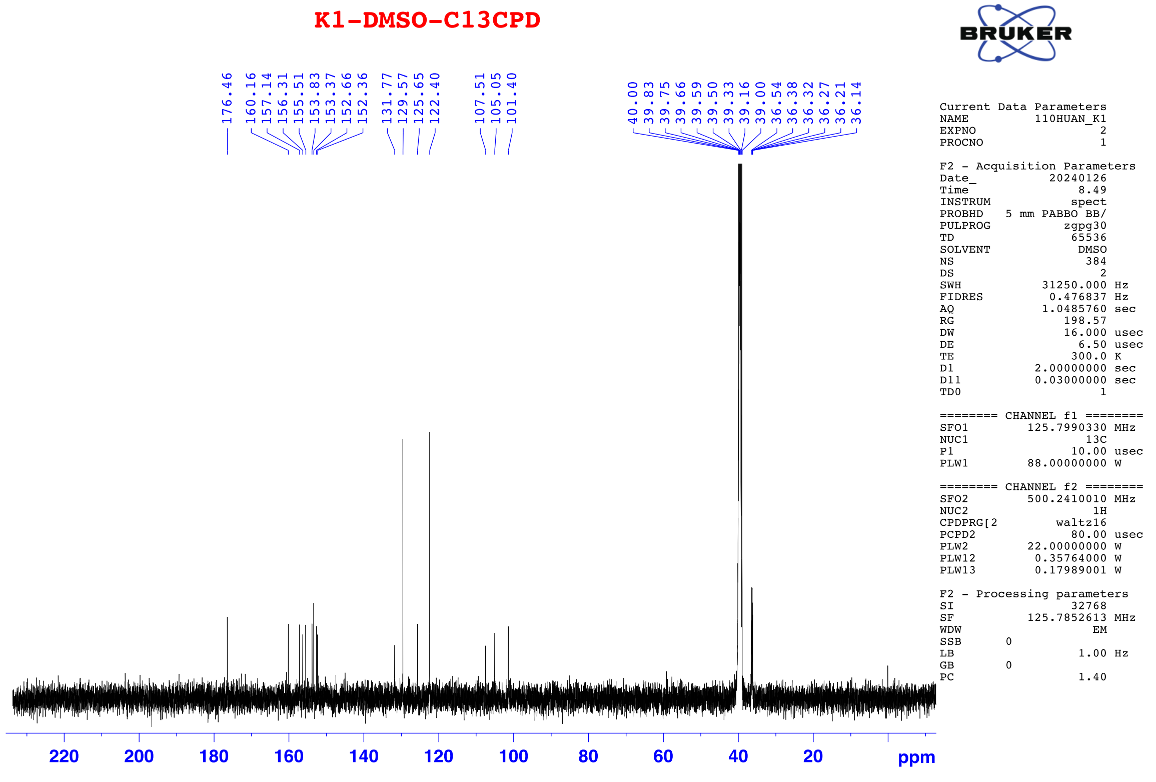

Fig. S7. Spectral data of compound **K1**

A. UV spectrum B. HRMS spectrum C. ^{1}H-NMR spectrum D. ^{13}C-NMR spectrum

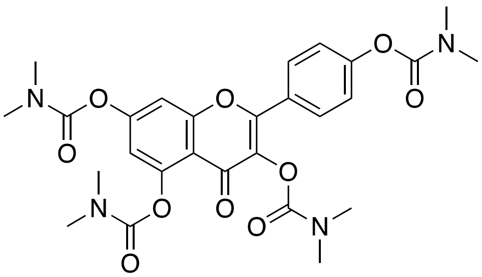

(A) UV spectrum of K2

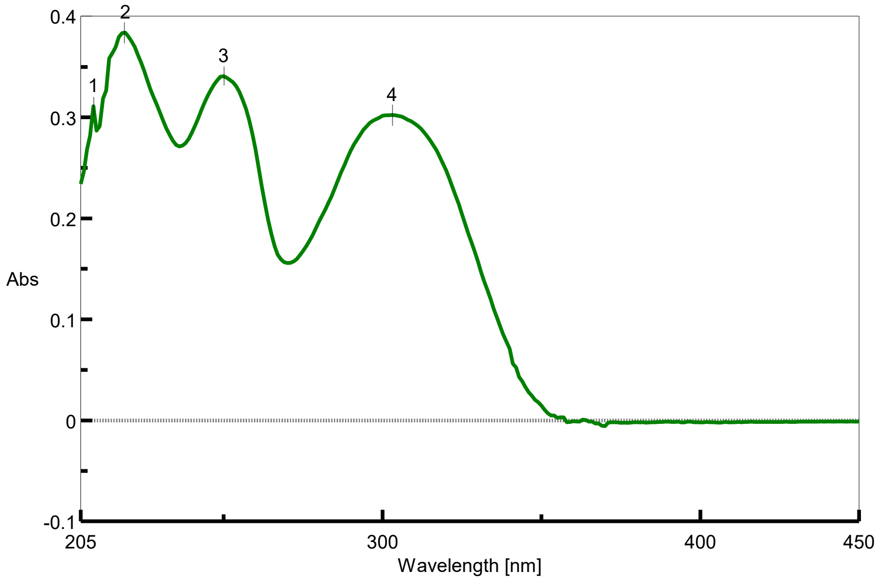

(B) HRMS spectrum of K2

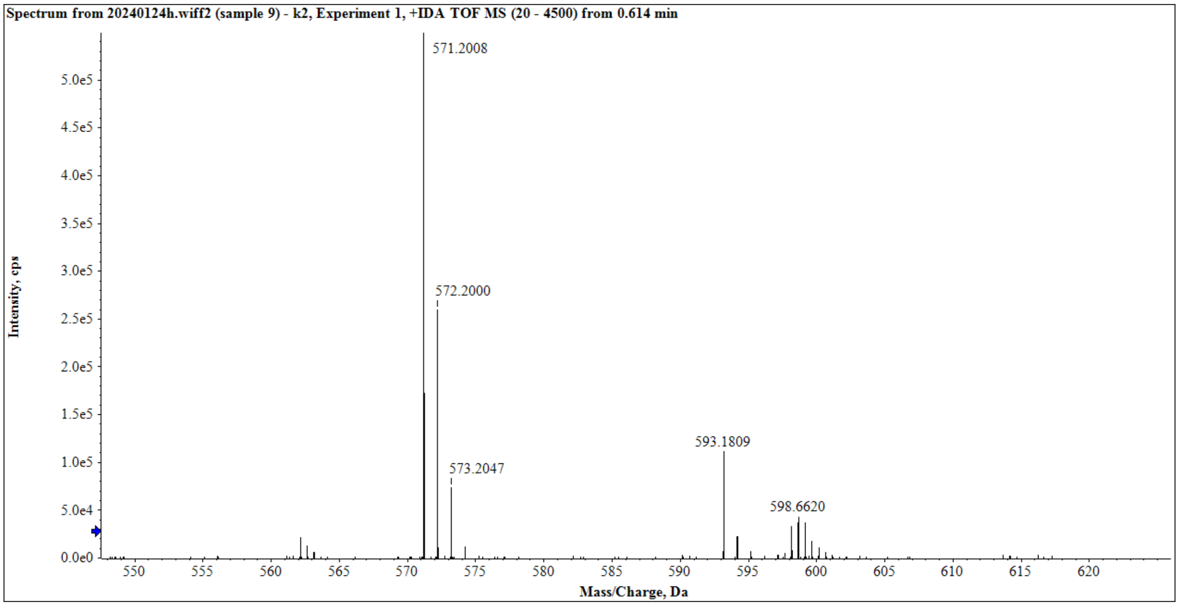

(C) ^{1}H-NMR spectrum of K2

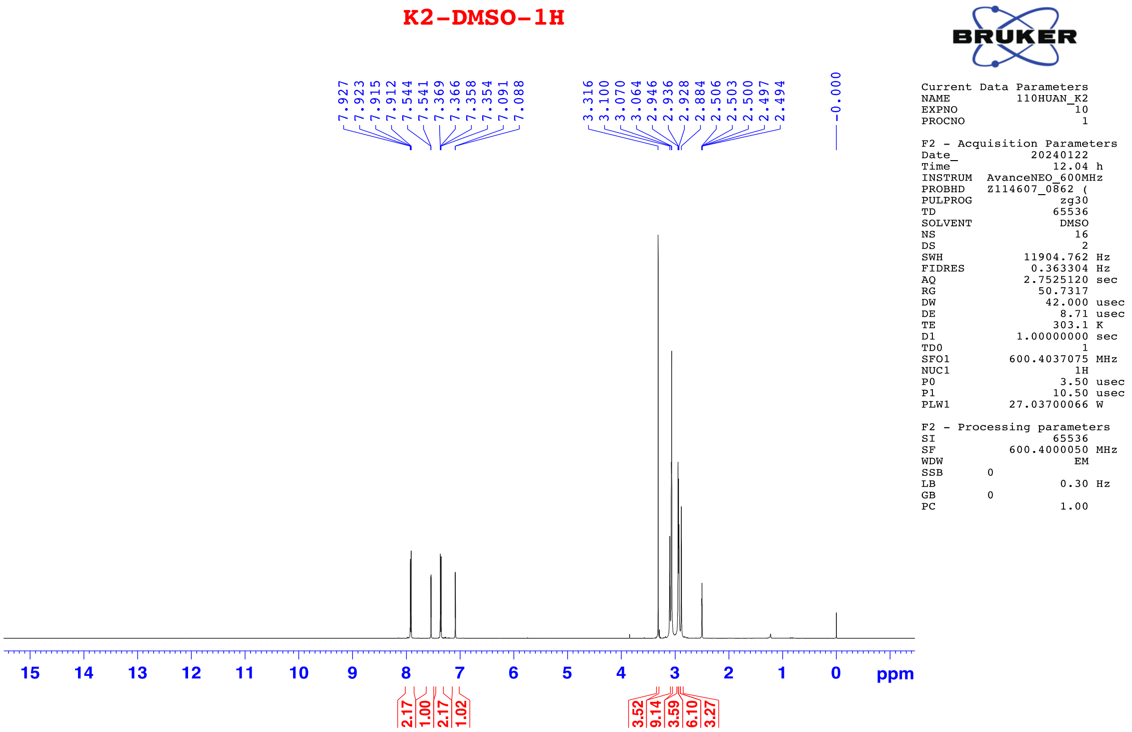

(D) ^{13}C-NMR spectrum of K2

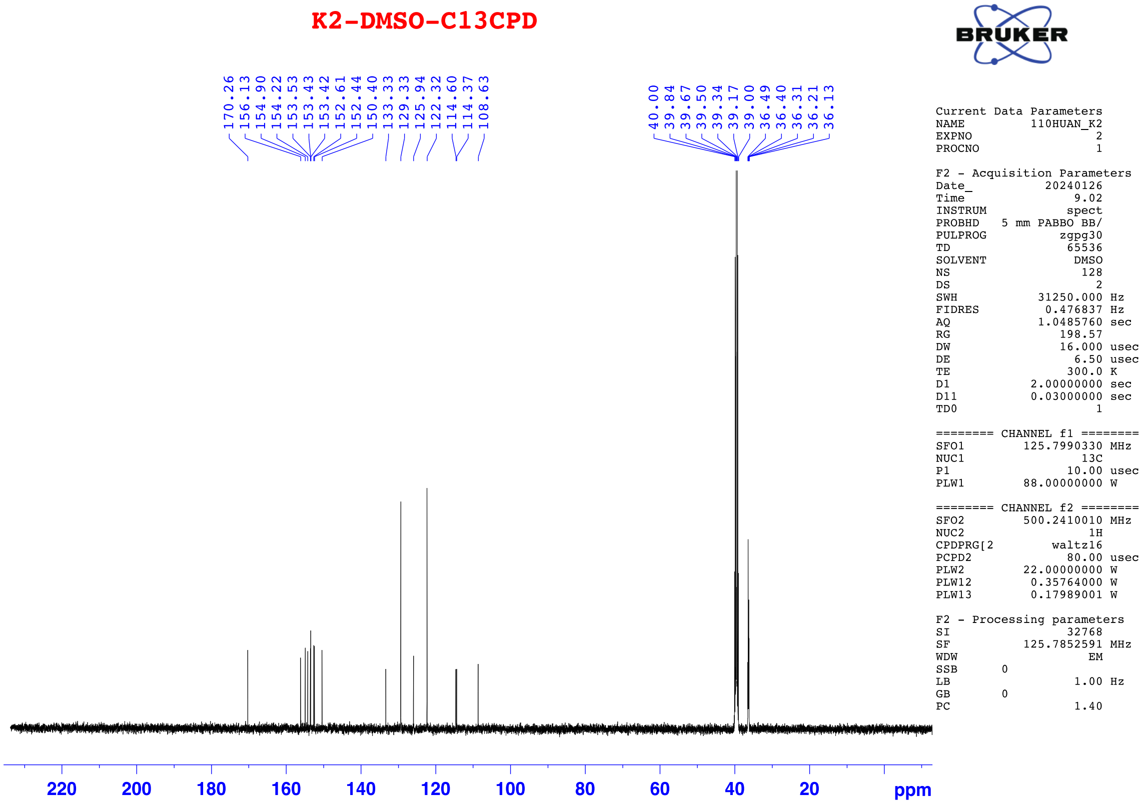

Fig. S8. Spectral data of compound **K2**

A. UV spectrum B. HRMS spectrum C. ^{1}H-NMR spectrum D. ^{13}C-NMR spectrum

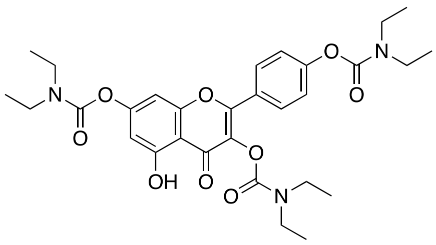

(A) UV spectrum of K3

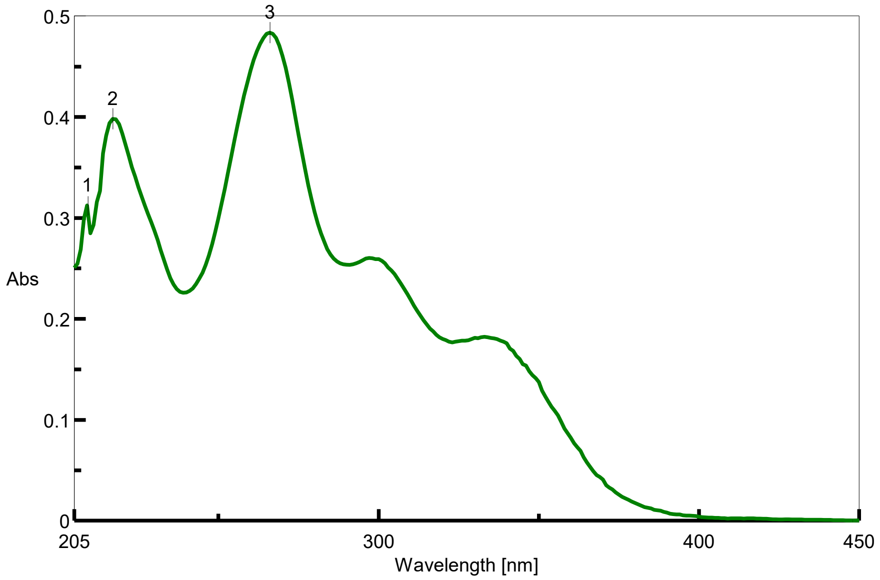

(B) HRMS spectrum of K3

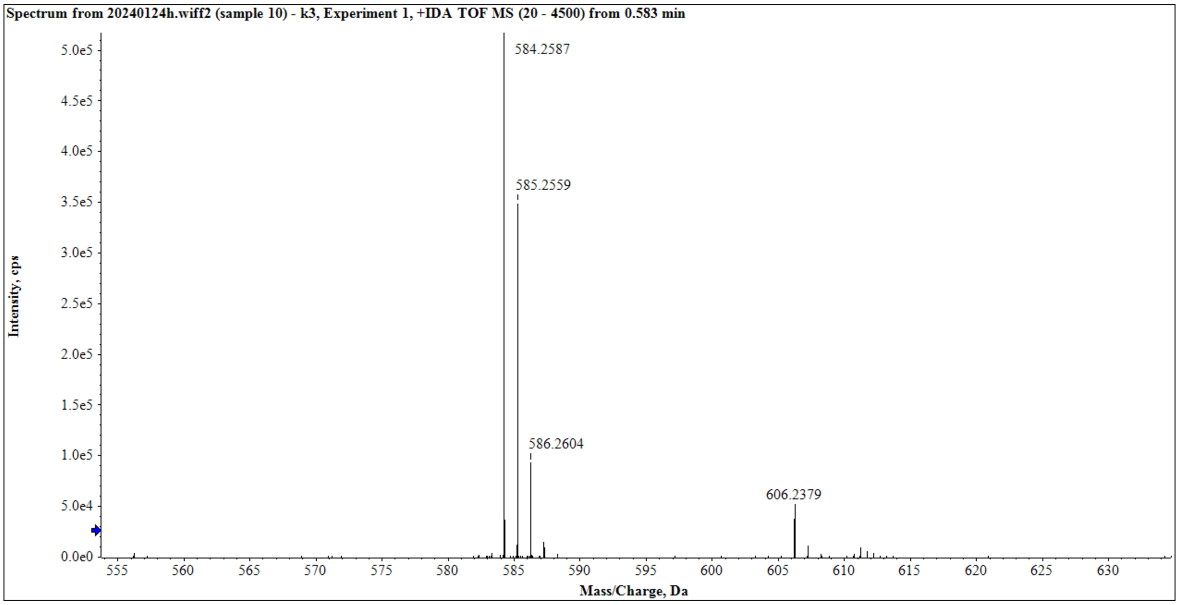

(C) ^{1}H-NMR spectrum of K3

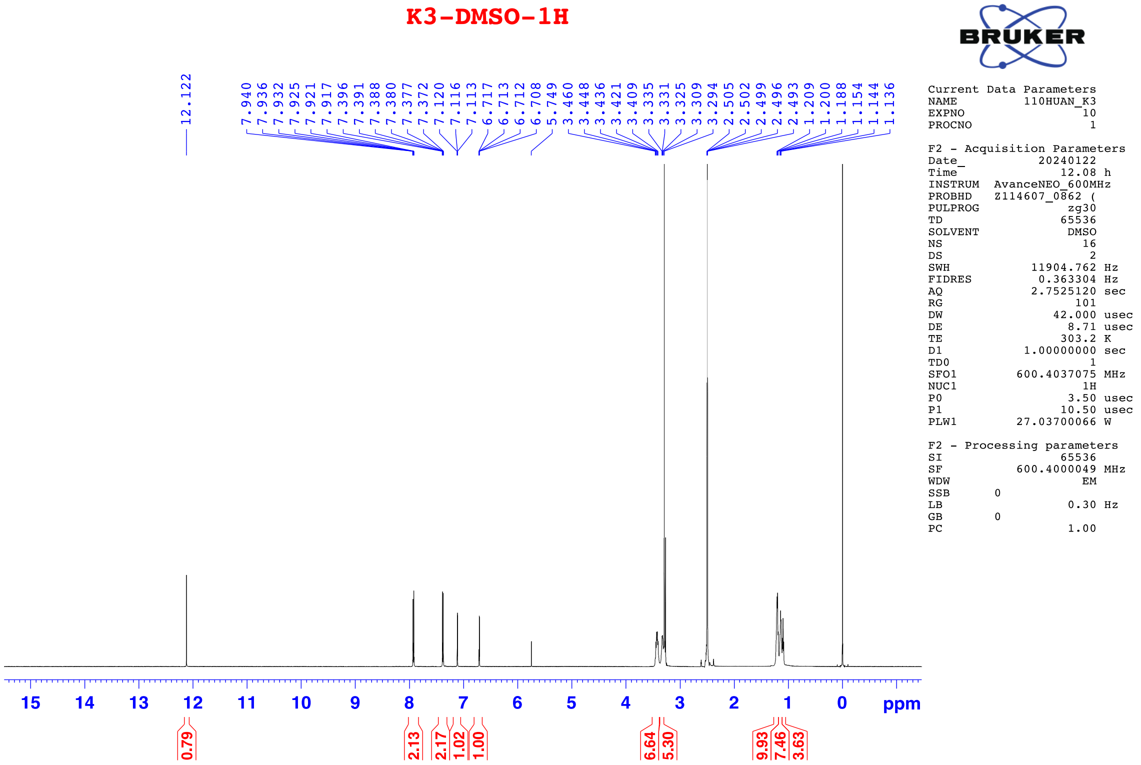

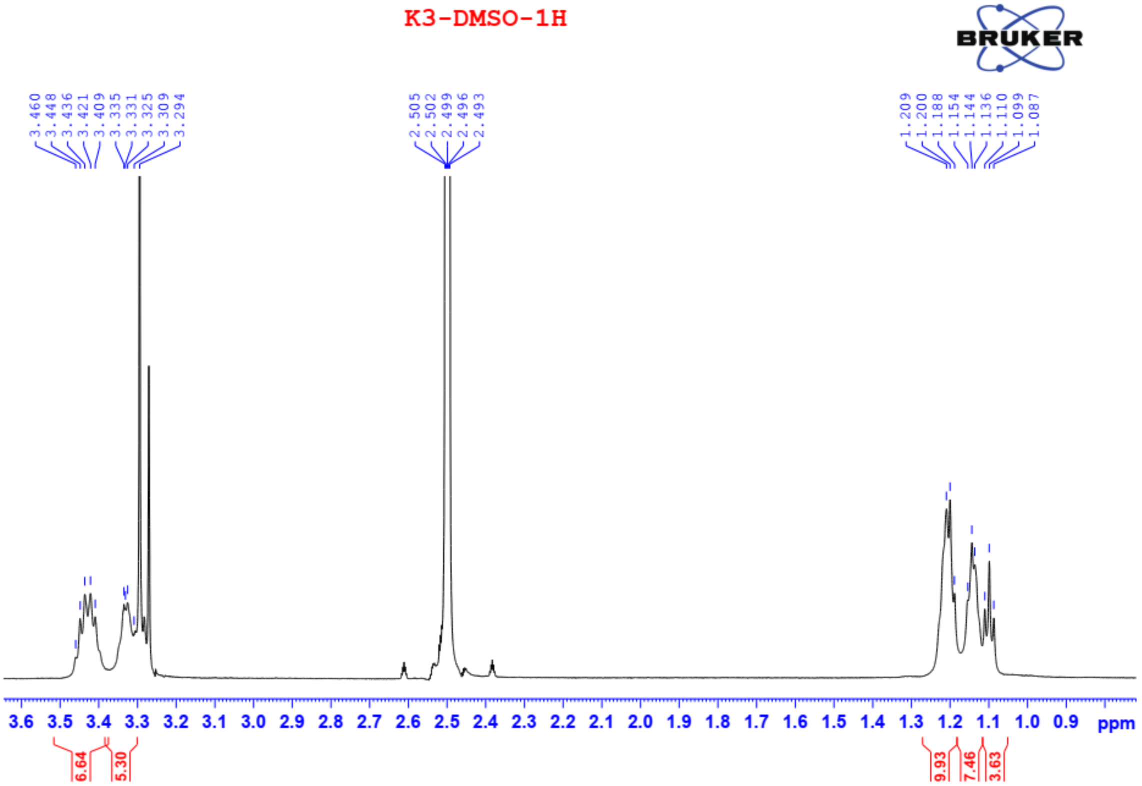

(D) ^{13}C-NMR spectrum of K3

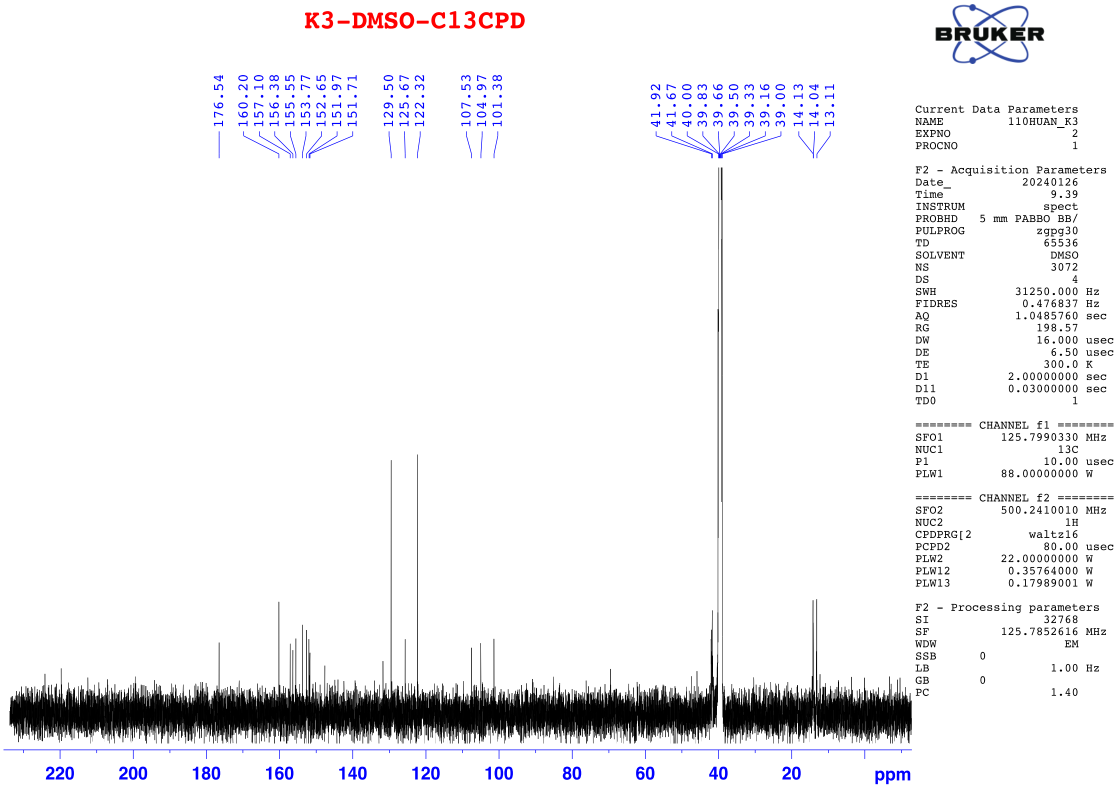

Fig. S9. Spectral data of compound **K3**

A. UV spectrum B. HRMS spectrum C. ^{1}H-NMR spectrum D. ^{13}C-NMR spectrum

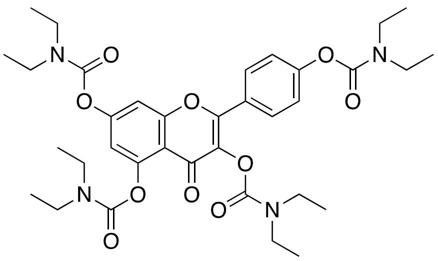

(A) UV spectrum of K4

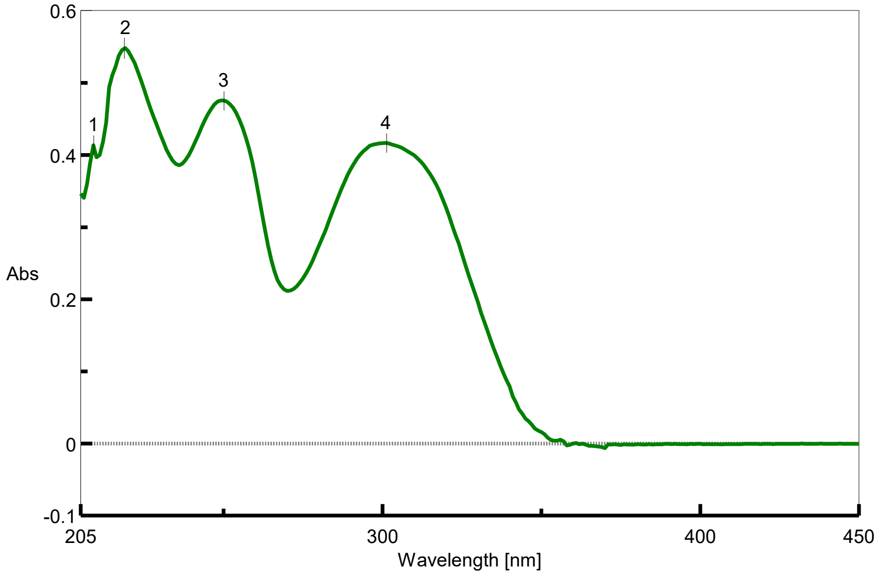

(B) HRMS spectrum of K4

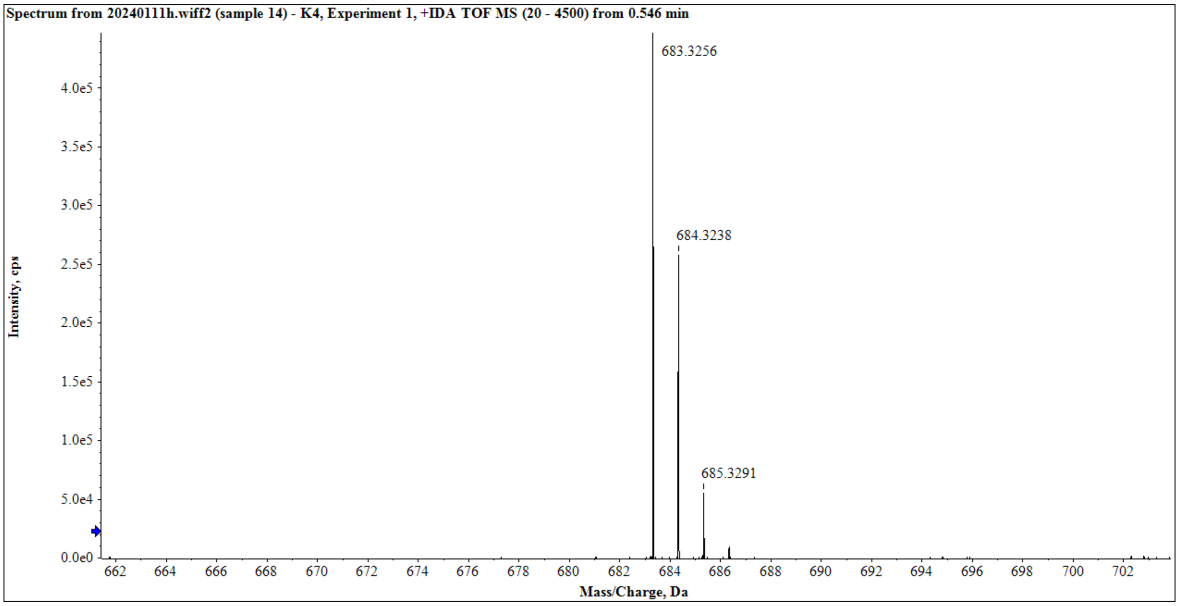

(C) ^{1}H-NMR spectrum of K4

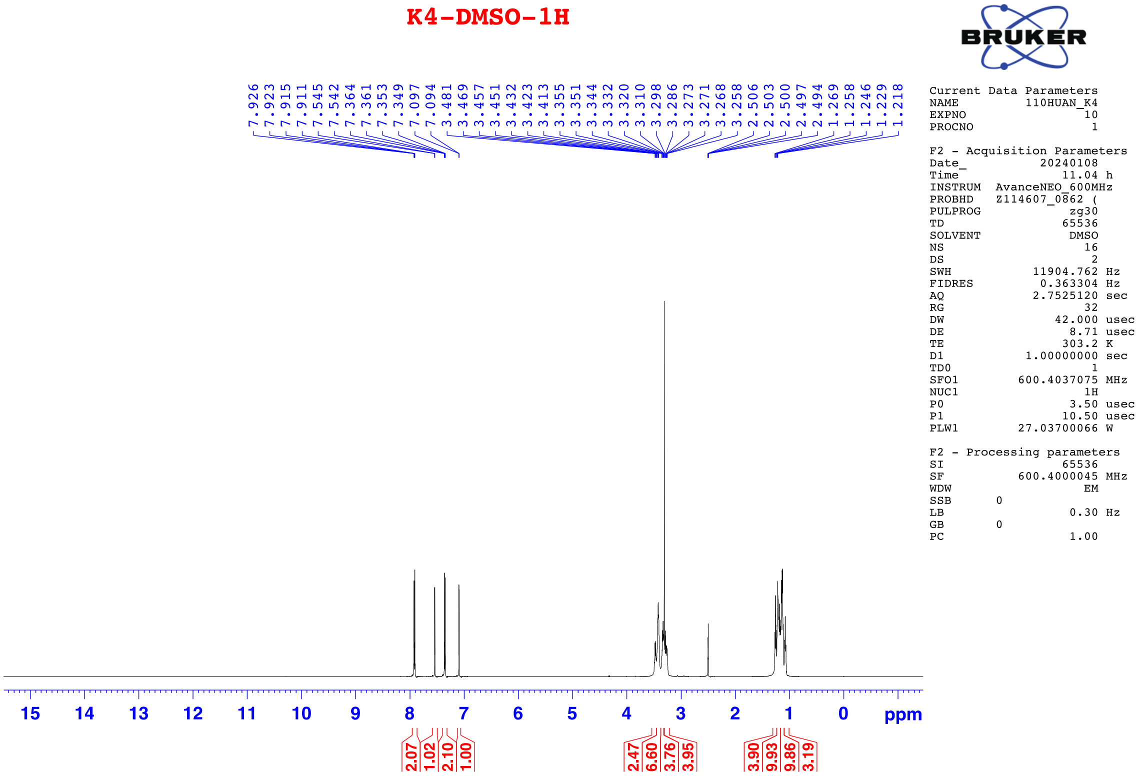

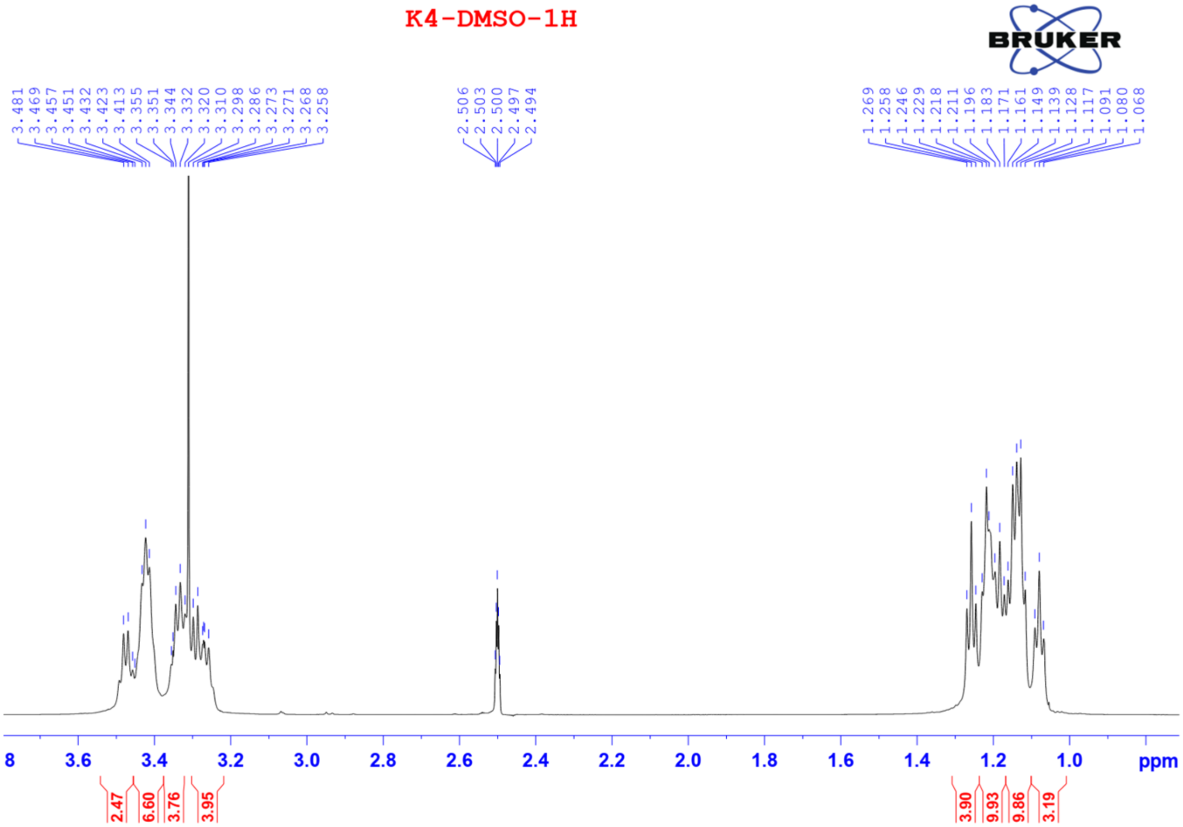

(D) ^{13}C-NMR spectrum of K4

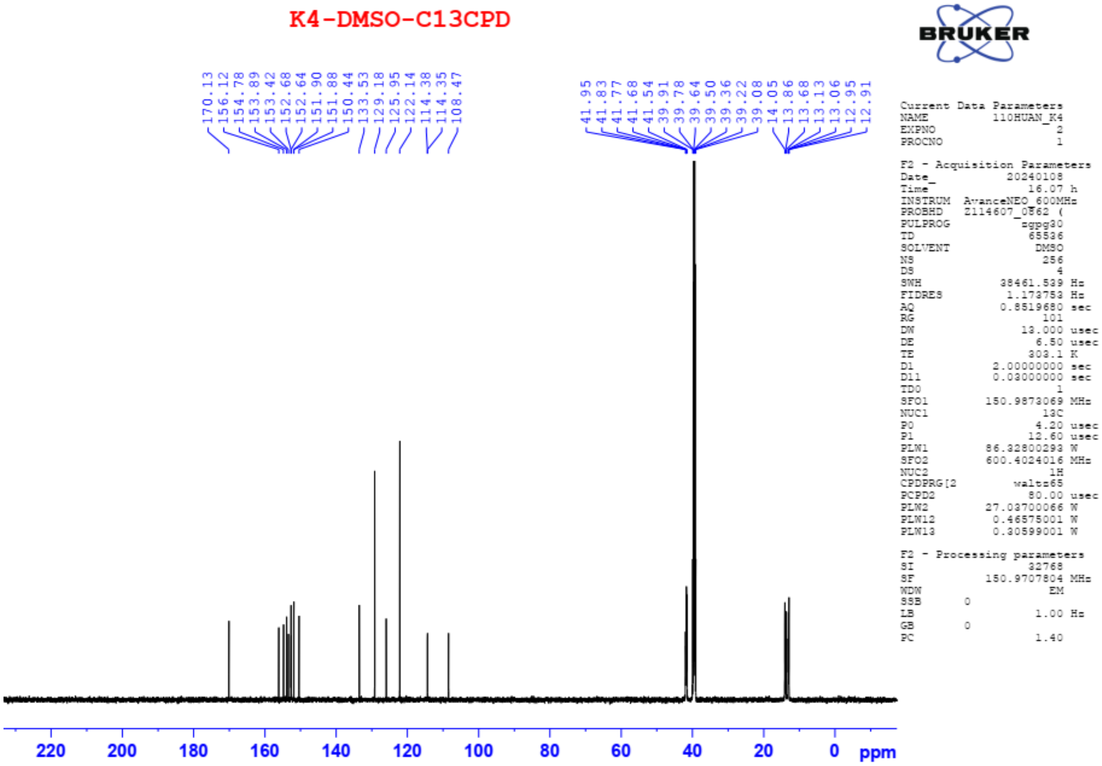

Fig. S10. Spectral data of compound **K4**

A. UV spectrum B. HRMS spectrum C. ^{1}H-NMR spectrum D. ^{13}C-NMR spectrum

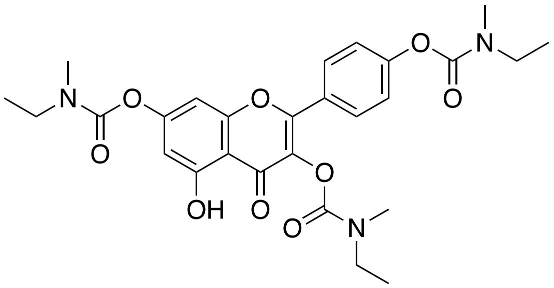

(A) UV spectrum of K5

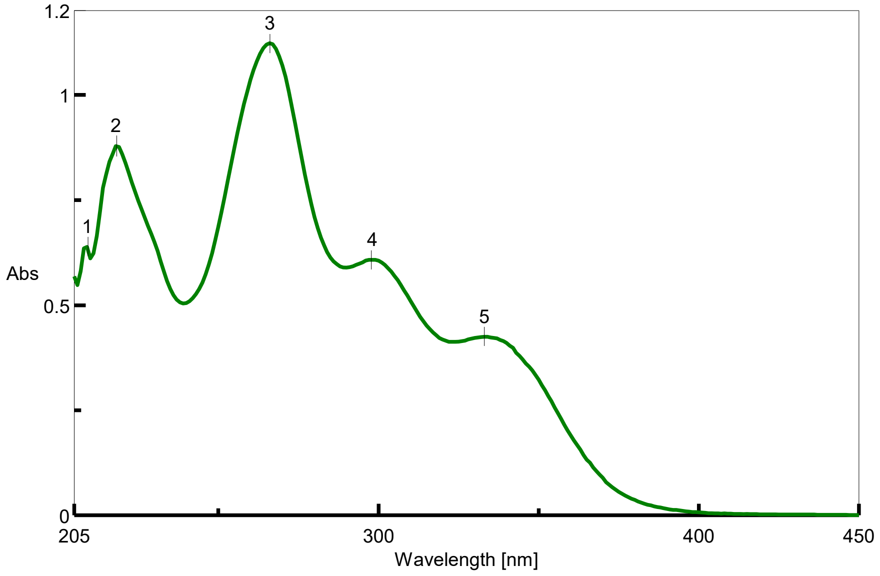

(B) HRMS spectrum of K5

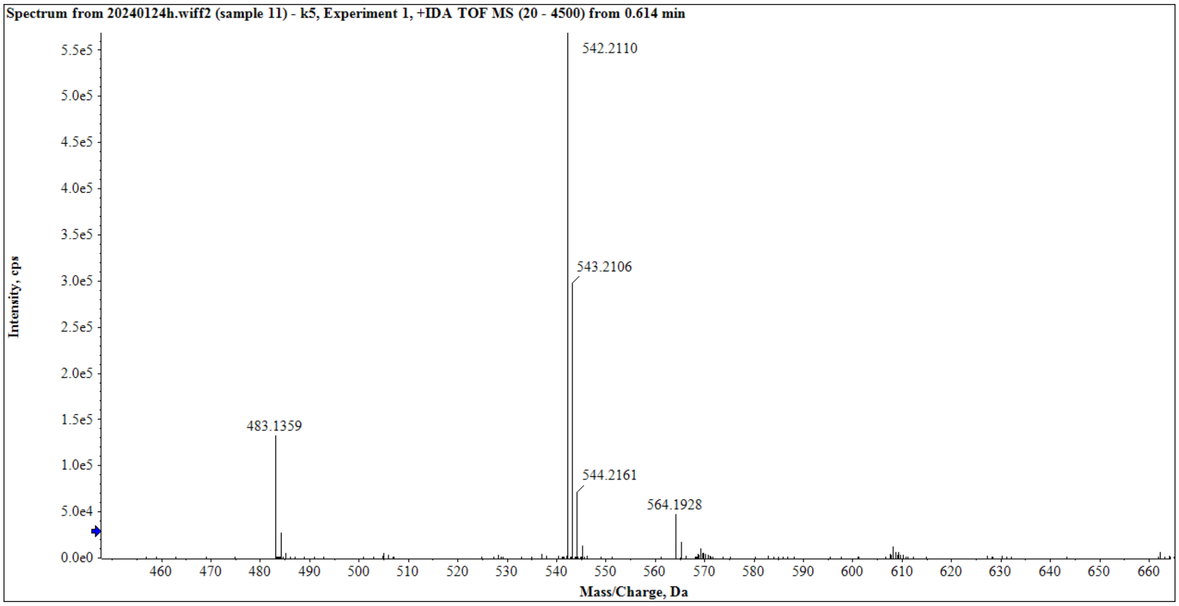

(C) ^{1}H-NMR spectrum of K5

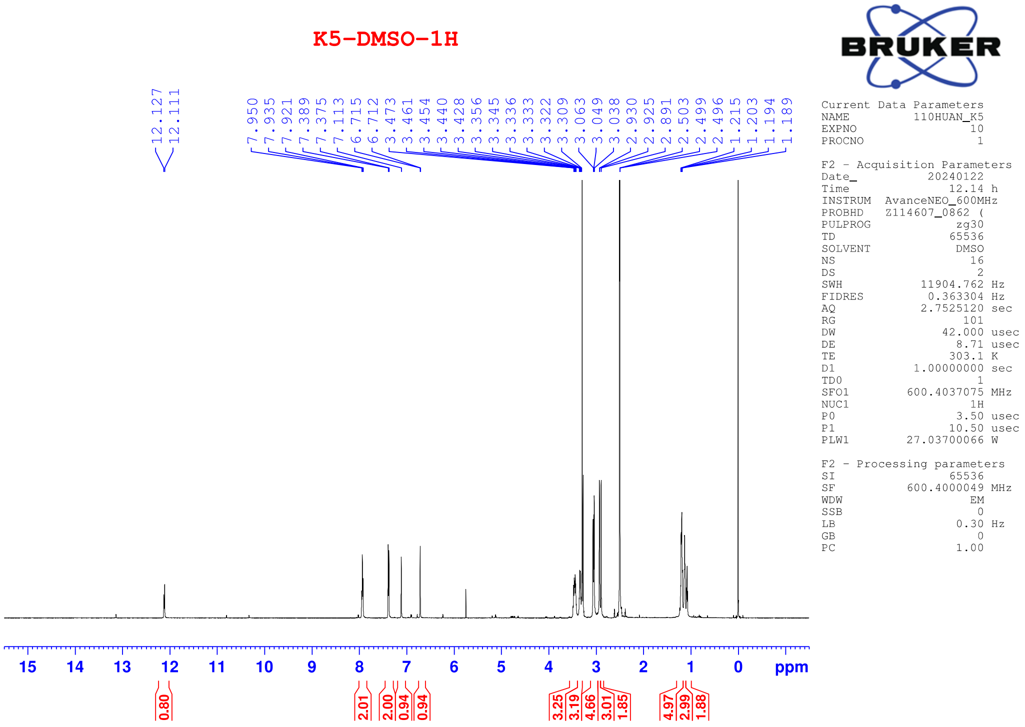

(D) ^{13}C-NMR spectrum of K5

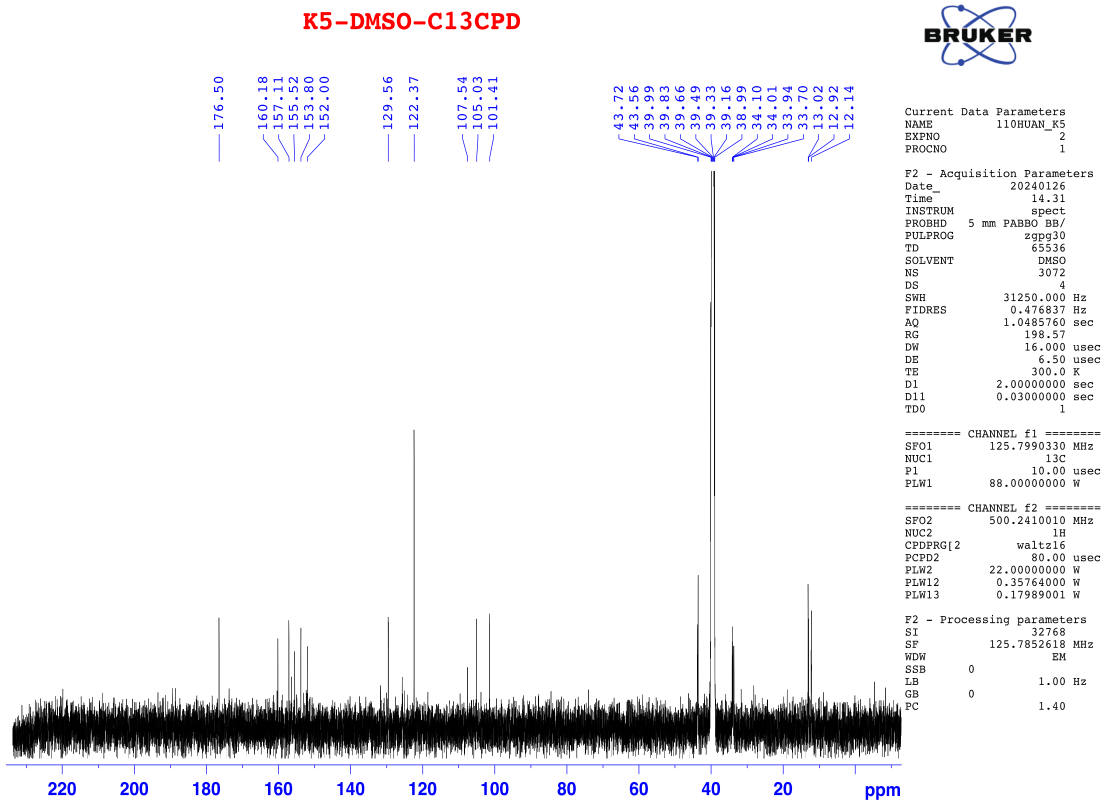

Fig. S11. Spectral data of compound **K5**

A. UV spectrum B. HRMS spectrum C. ^{1}H-NMR spectrum D. ^{13}C-NMR spectrum

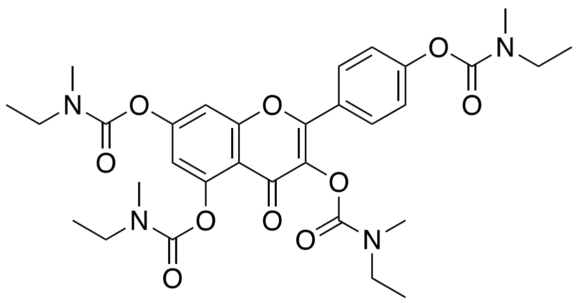

(A) UV spectrum of K6

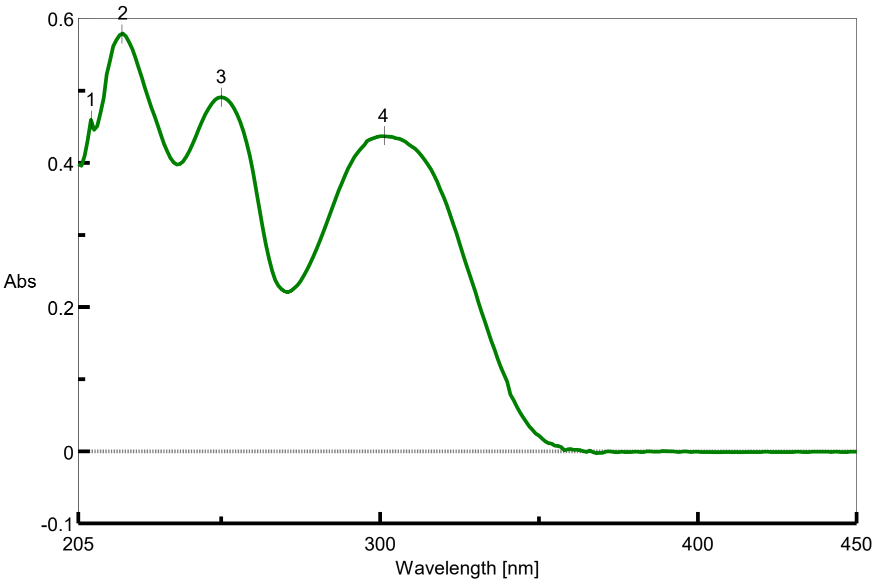

(B) HRMS spectrum of K6

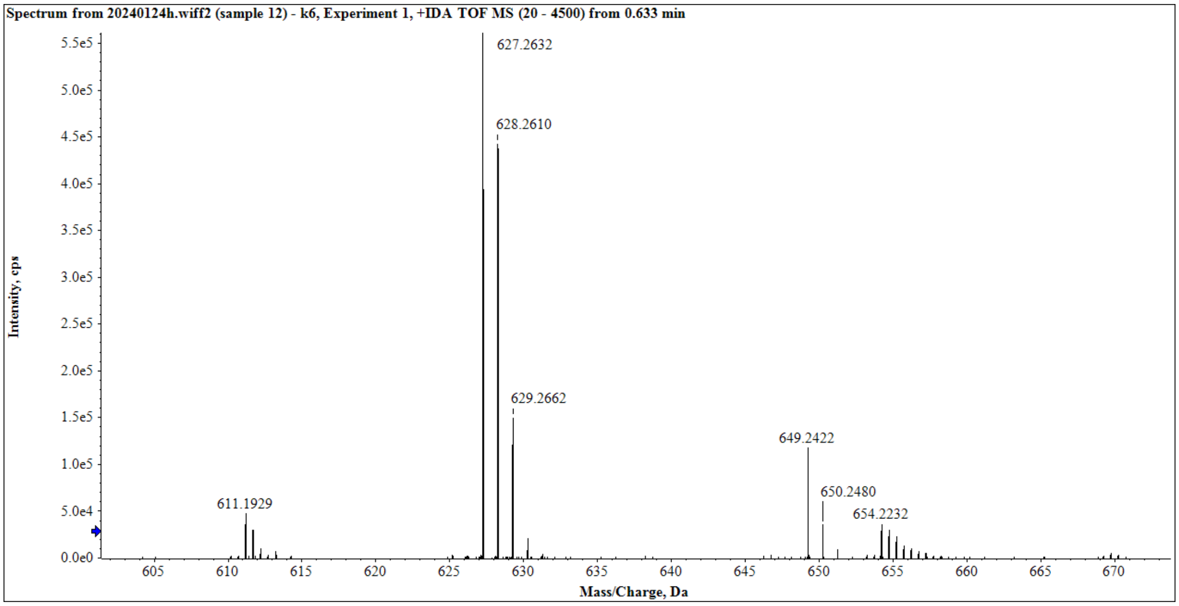

(C) ^{1}H-NMR spectrum of K6

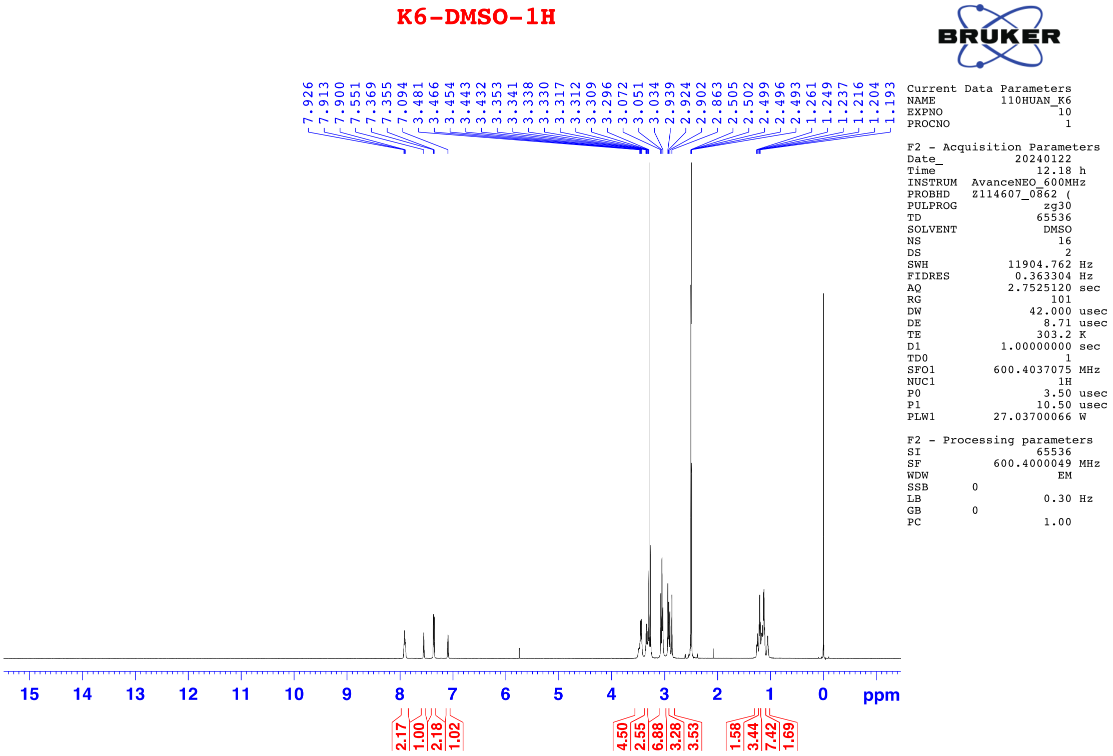

(D) ^{13}C-NMR spectrum of K6

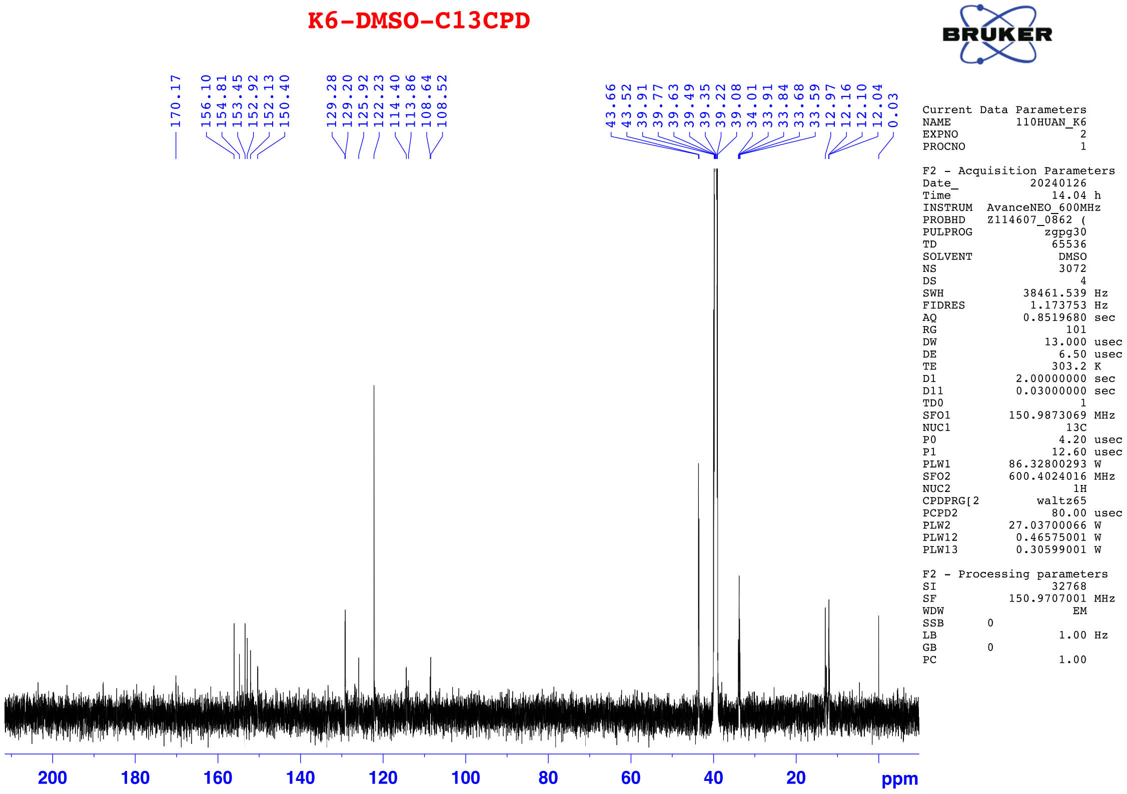

Fig. S12. Spectral data of compound **K6**

A. UV spectrum B. HRMS spectrum C. ^{1}H-NMR spectrum D. ^{13}C-NMR spectrum
